# Revealing the Nature of Non‐Covalent Interactions in Ionic Liquids by Combined Pulse EPR and ^19^F NMR Spectroscopy

**DOI:** 10.1002/anie.202504882

**Published:** 2025-05-10

**Authors:** Ciarán J. Rogers, Spyridon Koutsoukos, Jana Eisermann, Luke Wylie, Gavin J. Smith, Tom Welton, Maxie M. Roessler

**Affiliations:** ^1^ Department of Chemistry Imperial College London Molecular Sciences Research Hub London W12 0BZ UK; ^2^ Centre for Pulse EPR Spectroscopy (PEPR) Imperial College London Molecular Sciences Research Hub London W12 0BZ UK; ^3^ Mulliken Center for Theoretical Chemistry University of Bonn, Institute for Physical and Theoretical Chemistry Beringstr. 4 D‐53115 Bonn Germany; ^4^ Present address: Department of Chemistry, University of Stuttgart Institute of Physical Chemistry Pfaffenwaldring 55 70569 Stuttgart Germany

**Keywords:** Ionic liquids • Non‐covalent interactions• Paramagnetic relaxation enhancement • Pulse electron paramagnetic resonance • Solute‐solvent interactions

## Abstract

Ionic liquids (ILs) are a unique class of compounds that have attracted interest for numerous and diverse applications, ranging from solvents for sustainable synthesis to sustainable electrolytes. Understanding their nanostructure and solute‐solvent interactions is a prerequisite to harnessing the full potential of ILs. It has been proposed that ILs solvate non‐polar solutes via their alkyl chains through the formation of nanoscale structures, such as micelles. Here, we determine the non‐covalent interactions responsible for such nanostructuring in ILs. We use pulse electron paramagnetic resonance (EPR), paramagnetic relaxation enhancement (PRE) NMR, molecular dynamics (MD), and density functional theory (DFT) calculations in combination with ILs tailored to probe specific interactions through spin and isotopic labelling. Inter‐ and intramolecular cation–anion interactions are probed by electron‐nuclear double resonance (ENDOR) and ^19^F PRE experiments and show that nitroxide solutes associate with the polar domains of the imidazolium cation through weak hydrogen bonding with the imidazolium ring protons, as supported by MD simulations. Thus, this study reveals a less structured nanostructure than a micellar picture might suggest, but with clear IL cation‐solute interactions. Our methodology to reveal nanostructure not only has implications for ILs but is also applicable to other soft matter systems.

## Graphical abstract

Ionic liquids have been proposed as alternatives to molecular solvents. Here, spin probes and spin‐labelled ILs are studied by pulse electron paramagnetic resonance, paramagnetic relaxation enhancement NMR, molecular dynamics, and density functional theory, revealing the IL interaction sphere. The results challenge previous interpretations of nitroxide‐solvent nanostructuring in ILs.

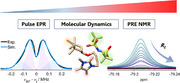

## Introduction

Ionic liquids (ILs) are systems composed entirely of ions that are liquid at the temperature of interest; they are solvents with properties distinct from those of conventional molecular solvents.^[^ ^1^, ^2^ ^]^ In recent years, the study of ILs has expanded to almost every aspect of chemical research, ranging from non‐aqueous electrolytes^[^ ^3^ ^]^ and carbon capture^[^ ^4^ ^]^ to biological applications such as protein crystallisation^[^ ^5^, ^6^ ^]^ and drug delivery.^[^ ^7^, ^8^ ^]^

Much attention has focused on the structure and dynamics of imidazolium‐based ILs.^[^ ^9^, ^10^ ^]^ These ILs feature an organic or inorganic anion, and an organic amphiphilic cation, consisting of a polar imidazolium core and a non‐polar alkyl chain. The diverse choice of anionic components and the degree of cationic alkylation imparts tunability and distinct physicochemical properties to these ILs. Hence, ILs are often called “designer solvents”,^[^ ^11^ ^]^ particularly with regard to their use in organic chemistry.^[^ ^12^ ^]^ Imidazolium‐based ILs are typically non‐volatile and non‐flammable, facilitating their recovery and reuse.^[^ ^13^, ^14^ ^]^ ILs have therefore also been demonstrated to enhance the sustainability of many chemical processes.^[^ ^14^, ^15^, ^16^ ^]^ Their application as alternatives to aqueous electrolytes arises from their non‐flammability, their large electrochemical windows, and favorable conductance properties.^[^ ^17^ ^]^ Indeed, recent computational and experimental studies demonstrated enhanced electrochemical control by judicious choice of IL‐based electrolytes.^[^ ^18^, ^19^ ^]^

The unique physicochemical properties of ILs arise because of their structures and the solvent environments they present.^[^ ^20^ ^]^ Although ILs share some similarities with other complex liquid systems, such as molten salts, liquid crystals, and glasses, the combination of physicochemical properties are unique to ILs.^[^ ^21^, ^22^, ^23^, ^24^ ^]^ It is well known that ILs have significant nanostructuring in the liquid state, with segregation of the imidazolium cation into polar and non‐polar domains, as determined primarily by molecular dynamics (MD) simulations and small‐angle X‐ray scattering techniques.^[^ ^25^, ^26^ ^]^ Solute–solvent interactions in ILs have also attracted much interest.^[^ ^27^ ^]^ In addition to the ubiquitous Coulombic interactions, hydrogen bonding, π–π interactions and dispersion interactions have all been found to be significant.^[^ ^28^ ^]^ These interactions are dependent upon the complementarity of the solute and the ILs’ ions, e.g. hydrogen bond donor solute with hydrogen bond acceptor anion, hydrogen bond acceptor solute with hydrogen bond donor cation, π–π interactions between aromatic solutes and imidazolium ions, and dispersion interactions between non‐polar solutes and long alkyl chain cations.^[^ ^29^ ^]^ Despite the fact that ILs have been intensively studied since the early 1990's, widespread industrial applications are still limited.^[^ ^30^ ^]^ One bottleneck is the lack in understanding of how inter‐ and intra‐ molecular interactions lead to macroscopic properties of ILs, making process optimisation difficult.^[^ ^31^ ^]^ Therefore studies on the structure and dynamics of ILs are more relevant than ever, as understanding the fundamental properties of ILs is key to their large‐scale applications. In addition to deepening understanding of ILs, findings from their chemistry can be transferred to other soft matter systems such as polymers, bio‐membranes, gels, liquid crystals, and nanocolloids. Despite the intense research activity aimed at understanding IL nanostructuring, solute dynamics, and solute–solvent interactions, these have largely been parallel activities and have not yet been brought together in a single study. Strikingly, the nature of the dynamic equilibrium between the nanodomains of solutes that experience both the ionic and the non‐polar nanodomains, and interfacial phenomena between these, remains unclear.

Magnetic resonance techniques are ideally suited to report on the heterogeneity of the IL nanoenvironment, nanostructure, equilibria dynamics and solute–solvent interactions therein. To date, continuous wave (CW) electron paramagnetic resonance (EPR) and magnetic relaxation measurements have predominantly been employed to probe the environment and dynamics of imidazolium‐based IL nanodomains using paramagnetic spin probes, primarily nitroxide‐based radicals,^[^ ^32^, ^33^, ^34^, ^35^ ^]^ but also photoexcited triplet states.^[^ ^36^, ^37^ ^]^ CW studies have given insight to the microviscosity of the IL nanoenvironment,^[^ ^38^ ^]^ while field‐dependent relaxation measurements showed that the librational motions of various spin probes undergo an anomalous suppression around the ILs’ glass transition temperature (*T*_g_). This feature is ubiquitous in imidazolium‐based ILs and alkyl chain‐bearing non‐IL glasses, and is found to be spin probe independent, but dependent on the alkyl chain length.^[^ ^39^, ^40^, ^41^, ^42^ ^]^ Together with MD results it has therefore been suggested that the studied spin probes are generally located in a micelle‐like environment and solvated at the interface of the non‐polar alkyl chains of the ILs.^[^ ^43^ ^]^ EPR studies of IL self‐probes, i.e., paramagnetic ILs, reported directly on the heterogeneity of an all‐IL nanoenvironment and show close to ideal mixing, minimising disruptions from using non‐IL probes.^[^ ^44^, ^45^ ^]^ Notably, organic nitroxide‐based ILs show equal promise for spectroscopic investigations of ILs and as efficient electrolytes and redox mediators,^[^ ^46^ ^]^ or recoverable solvent‐free catalysts.^[^ ^47^ ^]^

Pulse hyperfine electron EPR spectroscopy is a suite of techniques that offers unparalleled sensitivity in revealing the local structure around unpaired electrons through their interactions with surrounding magnetically active nuclei.^[^ ^48^ ^]^ While pulse hyperfine methods have found great success in fields ranging from surface catalysis^[^ ^49^, ^50^ ^]^ to structural biology,^[^ ^51^, ^52^ ^]^ they have not yet been applied to IL systems. Of particular relevance are ^19^F Mims electron‐nuclear double resonance (ENDOR)‐based distance measurements in the 5–20 Å range that exploit the dipolar interaction between an unpaired electron and ^19^F nuclear spins. This has been recently demonstrated at W‐band (ca. 94 GHz) in model systems,^[^ ^53^, ^54^ ^]^ proteins,^[^ ^55^ ^–‐^ ^57^ ^]^ in‐cell,^[^ ^58^ ^]^ and also at more accessible Q‐band frequencies (ca. 34 GHz).^[^ ^59^ ^]^ Further, applications of ^2^H Mims ENDOR are attractive for elucidation of the H‐bonding environment in (per)deuterated samples.^[^ ^60^, ^61^ ^]^ This is facilitated by the hyperfine coupling values, and the dependence of the nuclear quadrupole coupling constant on the hybridisation of the ^2^H nuclear spin centre.^[^ ^62^ ^]^ We reasoned that in ILs it should thus be possible to employ Mims ENDOR methods to detect coupled nuclei to a solvated paramagnetic centre, thereby reporting on the structure of the local nuclear environment. Paramagnetic relaxation enhancement (PRE) NMR can also access electron‐nuclear distances, with the advantage that samples are measured in the liquid state (in contrast to the ENDOR measurements that are typically performed in frozen solutions <100 K).^[^ ^63^, ^64^ ^]^ Although not yet applied to ILs, PRE NMR of ^19^F nuclear spins has been demonstrated as a sensitive method for room temperature biomolecular structural determination in the 9–30 Å distance range.^[^ ^65^, ^66^, ^67^ ^]^ PRE NMR has also served as a validation tool for solid‐state ENDOR distance measurements.^[^ ^58^, ^68^ ^]^

**Figure 1:**
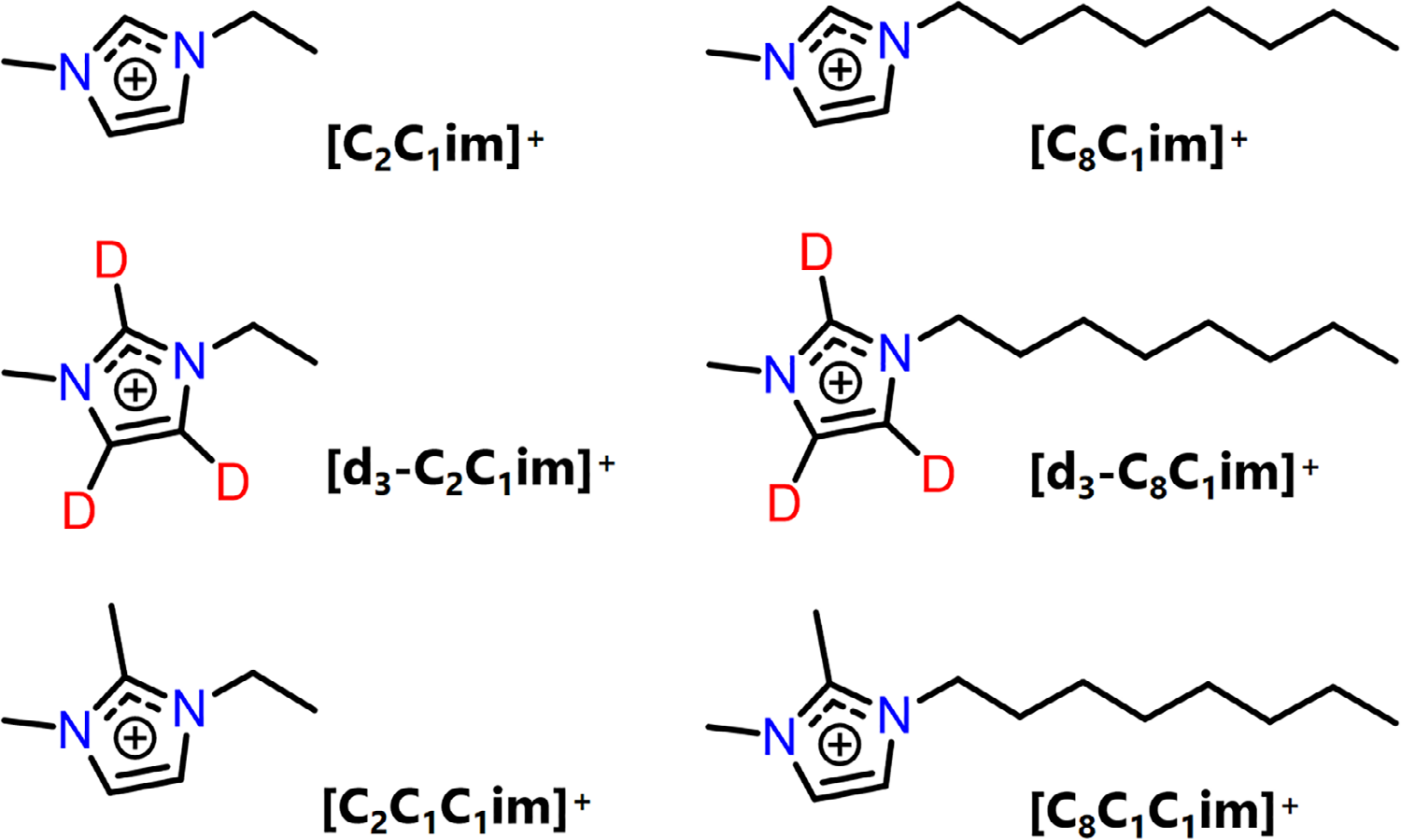
Ionic liquid cations synthesised and investigated in this work. Full synthetic details are available in Supporting Information S1.

In this work, we prepared nitroxide‐based spin probes dissolved in imidazolium‐based ILs (Figure 1) alongside a family of nitroxide spin‐labelled ILs dissolved in conventional molecular solvents, or in imidazolium‐based ILs. We apply pulse hyperfine EPR spectroscopy in combination with PRE NMR to investigate the non‐covalent interactions in ILs using both spin probes and spin‐labelled ILs. We determine the cation‐anion interaction strengths and the solute–solvent H‐bonding environment, providing detailed insight into the IL solvation nanostructure in the solid and liquid state. Density functional theory (DFT) calculations and MD simulations complement our magnetic resonance results. The toolkit of pulse hyperfine EPR, PRE NMR and computational techniques, shown here to elucidate IL solvation nanostructure and dynamics, thus paves the way to determine nanostructural organisation, equilibria dynamics and heterogeneity in similar semi‐structured soft matter systems.

## Results and Discussion

### Interactions of Spin Probes Dissolved in ILs

**Figure 2:**
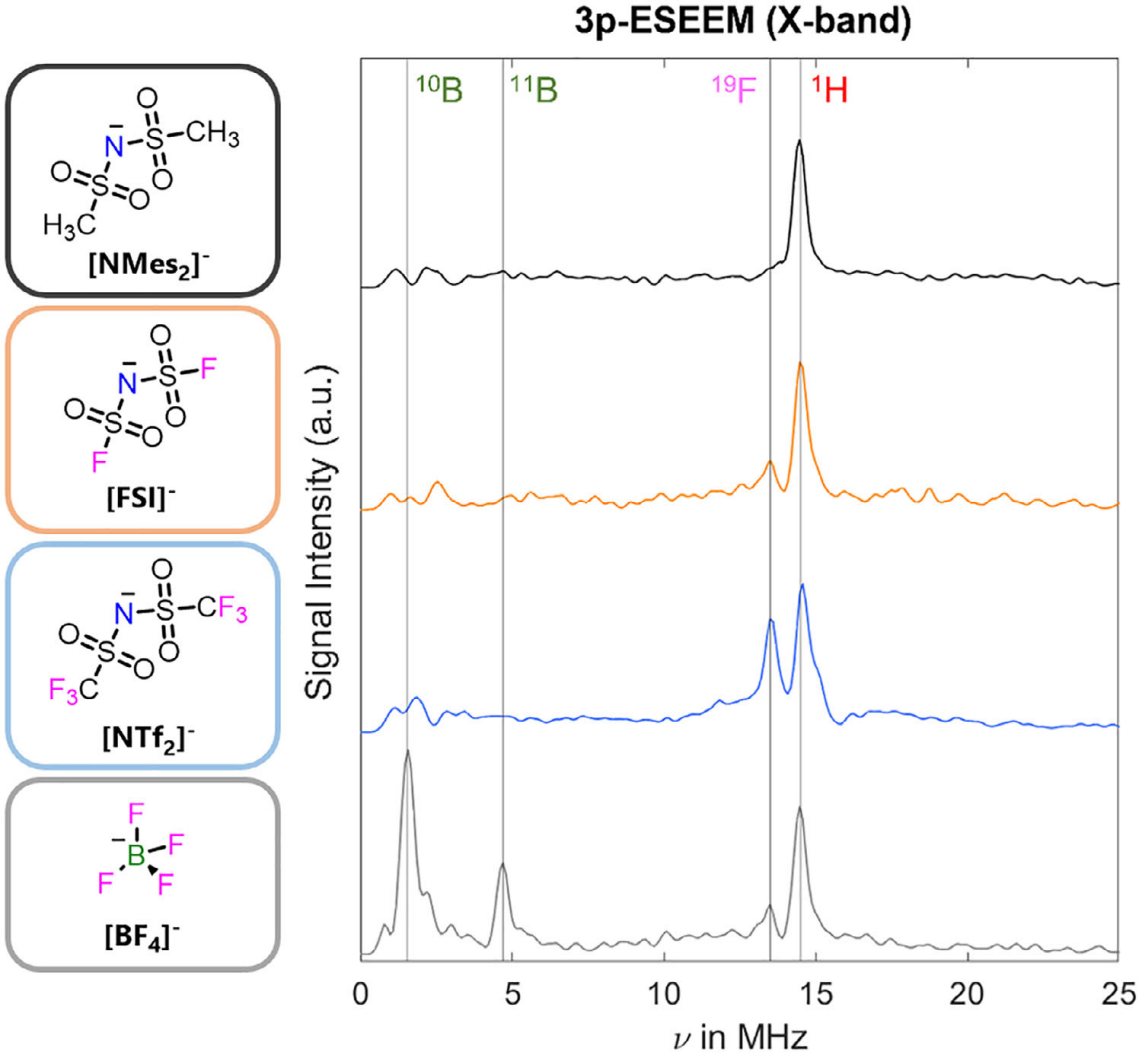
Identification of the most suitable anion through 3p‐ESEEM measurements. [C_2_C_1_im]^+^‐based ILs containing 2 mM of **TEMPOL** as a spin probe. Frequency peaks are shown from the Fourier transformed, *τ*‐summed, 3p‐ESEEM measurements (measured at 50 K), with the Larmor frequency of the corresponding nuclear isotope at X‐band shown for each anion studied and normalised to the ^1^H peak. All data sets were recorded at the maximum of the **TEMPOL** EDFS spectrum (Figure S1).

Much of the current literature focuses on the interaction of spin probes with the cationic component of ILs. However, it is well known that the anionic component is highly relevant in affording different physicochemical properties, including reactivity, acidity/basicity, hydrophobicity, and viscosity.^[^ ^20^, ^69^, ^70^ ^]^ Thus, we first determined the IL anionic environment of 4‐hydroxy‐2,2,6,6‐tetramethylpiperidin‐1‐oxyl (**TEMPOL**) as a spin probe dissolved in [C_2_C_1_im]^+^‐based ILs with different anions, namely [BF_4_]^−^, [NMes_2_]^−^, [NTf_2_]^−^ and [FSI]^−^ (Figure 2 and Supporting Information S.1 for synthetic details). Anions were selected based on the following criteria: a) they are each “weakly‐coordinating”,^[^ ^71^ ^]^ making a proof‐of‐concept study more feasible due to fewer possible interactions; b) they have large electrochemical windows, thermal and chemical stability, and thus present great interest for electrochemical and battery applications;^[^ ^72^, ^73^ ^]^ c) the synthetic methodology is well‐developed and results in high purity ILs, an important consideration for pulse EPR measurements, where signals from impurities could be detrimental.^[^ ^74^ ^]^

The echo‐detected field sweep (EDFS) spectra of **TEMPOL** dissolved in each IL were similar for all anions studied (Supporting Information S.2 and Figure S1). In order to gather information on the **TEMPOL** environment, 3‐pulse electron spin echo envelope modulation (3p‐ESEEM) experiments at X‐band were performed, detecting on the maximum of the **TEMPOL** signal at 50 K (Figures 2, S.2 and S.3 for details). The processed ESEEM spectrum showed distinct ^1^H peaks centred around their nuclear Larmor frequency for all systems, corresponding to the hydrogen atoms of both the cationic and anionic components of the studied ILs. For the [FSI]^−^ and [NTf_2_]^−^ anions, a ^19^F peak is observed which increases in intensity for the latter, due to an increasing number of ^19^F nuclei interacting with the **TEMPOL**. For the [BF_4_]^−^ sample the most pronounced peak seen is centred about the ^10^B Larmor frequency, while the ^11^B nuclear isotope is lower in intensity, and the ^19^F peak is suppressed. In all cases, it is evident that the spin probe has a hyperfine interaction with both the IL cation and anion. Based on the 3p‐ESEEM investigation, we decided to focus on the [NTf_2_]^−^ anion. First, the terminal trifluoromethyl (‐CF_3_) groups give rise to intense signals. Second, they offer the possibility to determine the cation‐anion interaction strength based on distances between the nitroxide spin centre and the [NTf_2_]^−^ anion using ^19^F Mims ENDOR.^[^ ^53^ ^]^

### Spin Probes and Spin‐labelled ILs

**Figure 3:**
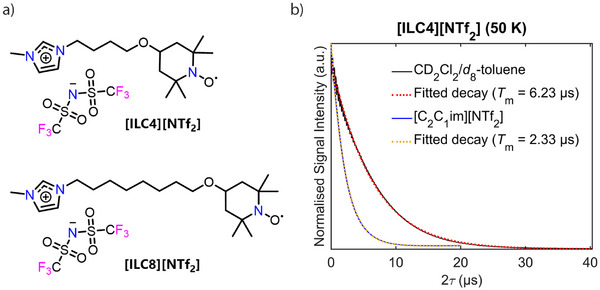
Spin‐labelled ILs and their relaxation properties. a) Chemical structures and abbreviations of the synthesised spin‐labelled ILs **[ILC4][NTf_2_]** and **[ILC8][NTf_2_]**. b) Representative experimental echo decay curves of a 0.2 mM solution of the spin‐labelled IL, **[ILC4][NTf_2_]**, in CD_2_Cl_2_/*d*_8_‐toluene (black) and [C_2_C_1_im][NTf_2_] (blue), measured at the maximum of the nitroxide EDFS at Q‐band, and the corresponding fitted stretched exponential decays (Supporting Information S2 for details, and S3 for further relaxation data).

Much of the structural research on ILs has been based on indirect measurements of model solutes, using a variety of techniques including infrared,^[^ ^75^ ^]^ Raman^[^ ^76^ ^]^ and ^1^H NMR.^[^ ^77^ ^]^ Welton et al. have studied the UV–vis spectra of organic dyes in ILs as a measure of their polarity extensively.^[^ ^78^, ^79^ ^]^ However, the selection of the dye can influence the measured polarity values,^[^ ^70^, ^80^ ^]^ indicating that the functionality of the solute can cause preferential solvation by the IL ions around it and hence influence the perceived polarity. Most existing IL‐related EPR studies focus on nitroxide‐based spin‐probes, such as **TEMPOL**. Although spin probes are extremely useful reporters of the IL nanoenvironment, it is not clear to what extent the use of a non‐IL probe impacts the nanostructuring of ILs. Spin‐labelling methods, akin to those used in the structural determination of biomolecules,^[^ ^81^ ^]^ applied to IL systems have the potential to allow direct measurements of an all‐IL nanoenvironment. There is only one prior EPR study employing spin‐labelled ILs, which demonstrated that non‐IL spin probe host‐guest type interactions with ILs are not responsible for the nanostructuring related to the anomalous suppression of molecular mobility around *T*_g_.^[^ ^45^ ^]^ Thus, in order to determine the non‐covalent interactions governing heterogeneity in an all‐IL system, the spin‐labelled ILs, **[ILC4][NTf_2_]** and **[ILC8][NTf_2_]** were synthesised (Figure 3a). The nitroxide spin‐labelled ILs are easily accessible, metal‐free, room temperature ILs. They exhibit characteristic nitroxide EDFS spectra (Figure S3) and a relatively long phase memory time (*T*_m_) at 50 K when dissolved in deuterated molecular solvents, as well as in ILs (Figure 3b).

### Cation‐Anion Associations Within Spin‐labelled ILs

Ionic compounds in solution experience different degrees of ionic association, depending on the polarity and the dielectric constant of the solvent. For molecular solvents this phenomenon is generally well understood, with high polarity solvents showing a low degree of ion association (free solvated ions and/or solvent‐separated ion pairs), while low polarity solvents show a high degree of ion association (penetrated and/or contact ion pairs). Ion pairing in ILs is more complex and has been widely studied both computationally and experimentally.^[^ ^82^, ^83^, ^84^ ^]^

**Figure 4:**
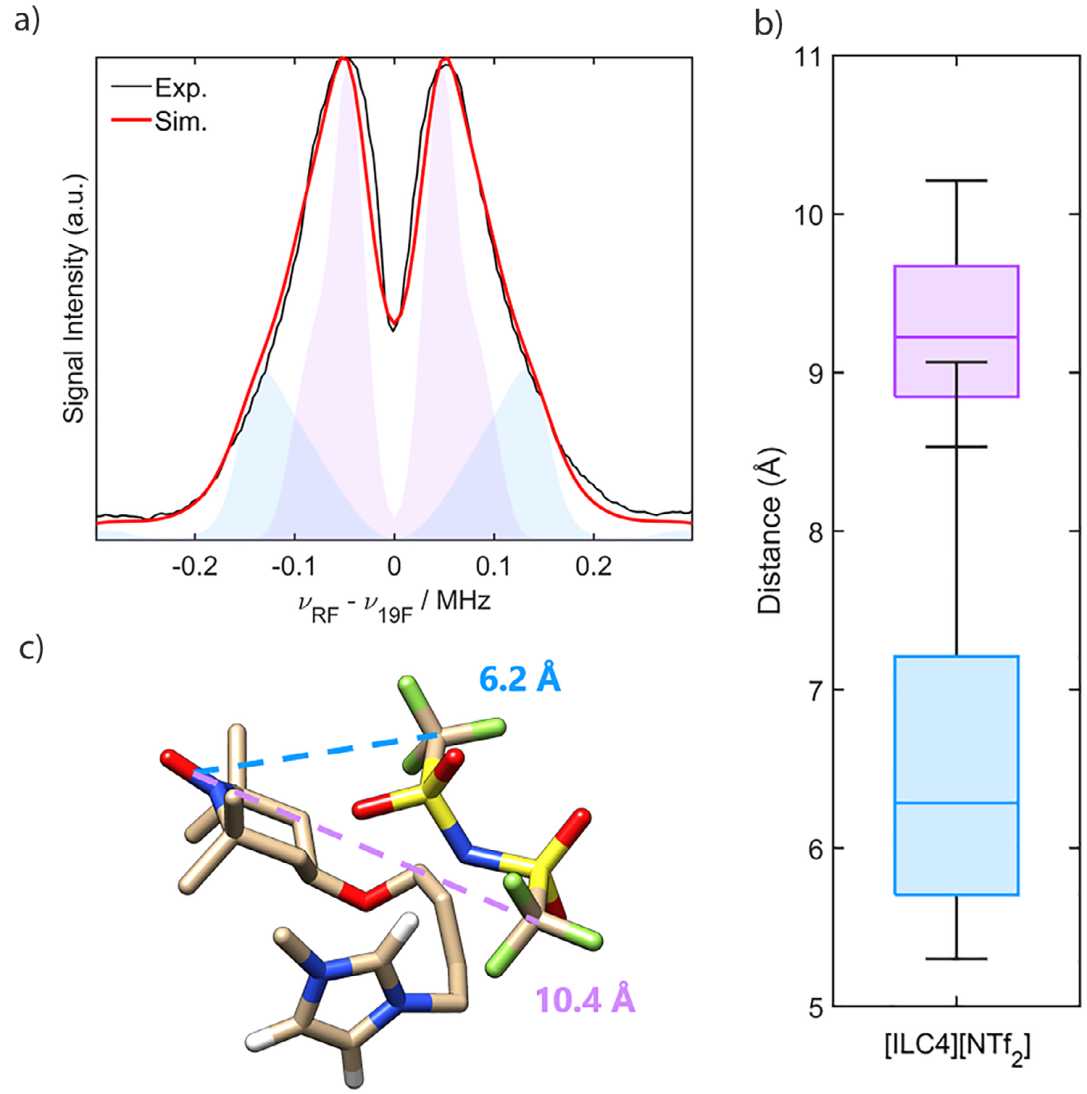
^19^F ENDOR‐derived nitroxide‐anion distances within spin‐labelled ILs in the solid‐state. a) Experimental ^19^F Mims ENDOR spectrum averaged over *τ* = 2 and 2.4 µs (black) of a flash‐frozen solution of **[ILC4][NTf_2_]** in CD_2_Cl_2_/*d*_8_‐toluene (0.2 mM) measured at Q‐band (50 K), overlaid with the corresponding summed simulations (red) considering two skewed, and weighted, normal distributions of *T*_⊥_ (purple and blue). b) Box and whisker plot of the distances determined by simulation of the experimental spectrum according to Equation 1, where the centre line of each box corresponds to the median nitroxide‐^19^F distance, *r̃*, the edges of the box correspond to the upper and lower quartiles, and the whiskers correspond to the maximum and minimum distances included in the simulation. c) DFT optimised conformer of **[ILC4][NTf_2_]** as described in the main text and in Supporting Information S4, showing the calculated distances between the centre of the N─O bond of the nitroxide and the centre of the CF_3_ groups. Non‐imidazolium ring hydrogens have been removed for clarity (red: O, green: F, blue: N, yellow: S, beige: C, white: H).

We can report on the cation‐anion interactions within the spin‐labelled ILs provided that the spin‐labelled IL ion pair is isolated (i.e., solvated in a molecular solvent matrix) and the spin‐label's interaction with the ^19^F nuclei of the [NTf_2_]^−^ anion can be probed. To determine the strength of the intramolecular ionic associations, ^19^F Mims ENDOR experiments were performed in dilute flash‐frozen solutions of the spin‐labelled ILs. This allows the dipolar interaction between the nitroxide labelled alkyl chain of the cation, and the terminal trifluoromethyl groups of the [NTf_2_]^−^ anion to be determined. Assuming a point‐dipole model, the principal axis of the dipolar part of the hyperfine interaction tensor, *T*_⊥_, as a function of the distance, *r*, between the centre of the nitroxide N‐O bond and the center of the trifluoromethyl group is then given by:

(1) $$\;T_{⊥}=\frac{\mu_{0}}{4\pi h}\;\frac{g_{e}g_{N}\mu_{e}\mu_{N}}{r^{3}}=\frac{74.52}{r^{3}}$$

where *g*_e_ and *g*_N_ are the *g* factors of the nitroxide centre and ^19^F nucleus, respectively, and *µ*_e_ and *µ*_N_ are the Bohr and nuclear magnetons. The parameters determined from the best‐fitting simulations of the experimentally recorded ^19^F Mims ENDOR spectra are summarised in Table 1. The observation of a clear interaction with ^19^F indicates, as expected, that the anion preferentially associates with the polar moiety of the cation (i.e., the imidazolium ring), rather than the non‐polar alkyl chain, in both **[ILC4][NTf_2_]** and **[ILC8][NTf_2_]**. As previously shown for non‐rigid unpaired electron‐^19^F dipolar vectors,^[^ ^54^ ^]^ the lineshape of the Mims ENDOR spectrum is dominated by a distribution of hyperfine couplings between the ^19^F nuclear spins of the two trifluoromethyl groups and the nitroxide, rather than appearing as a complete or partial Pake pattern. Therefore, the data was simulated considering two skewed and weighted normal distributions that consider a mean value of *T*_⊥_ for each of the two trifluoromethyl groups, and a distribution of *T*_⊥_ around the mean value (full simulation details available in Supporting Information). The experimental data could not be satisfactorily fitted to a single coupling distribution at a given *T*_⊥_ (Figure S8). This suggests that, in the solid‐state, the anion adopts a preferential conformation relative to the cation with a stronger and a weaker hyperfine interaction tensor (Figure 4a). The experiment was repeated under the same conditions for the longer alkyl chain sample, **[ILC8][NTf_2_],** and a strong and weak hyperfine interaction tensor was observed that could also only be fitted to two distinct coupling distributions, albeit with a larger lineshape broadening, suggesting higher conformational flexibility in this spin‐labelled IL (Figure S9). Due to the non‐linear relationship between *T*_⊥_ and the calculation of a skewed distribution of distances based on Equation 1, the distance analysis of the experimental data is best represented by a box and whisker plot (Figure 4b). No orientation‐selection was observed when changing the magnetic field position of the stimulated echo observer sequence (Figure S10). This is likely due to the variety of conformers contributing to the ENDOR signal, the orthogonality of the *g* and hyperfine tensor frames, and the fact that each ‐CF_3_ group consists of three non‐orthogonal nitroxide‐^19^F dipolar vectors.^[^ ^54^ ^]^

**Table 1:** Simulation parameters for the Q‐band ^19^F Mims ENDOR experiments on the spin‐labelled ILs isolated in a glassy deuterated solvent matrix.

Solvent matrix	IL	*τ* (µs)	lw^a)^ (KHz)	*T*_sim_^1^ ± σ (KHz)	*T*_sim_^2^ ± σ (KHz)
CD_2_Cl_2_/ *d*_8_‐toluene	**[ILC4] [NTf_2_]**	2.2	20	105 ± 40	300 ± 28
CD_2_Cl_2_/ *d*_8_‐toluene	**[ILC8] [NTf_2_]**	2.2	45	50 ± 35	220 ± 38

^a)^ Full‐width half maximum (FWHM) of a convoluted Lorentzian lineshape, where the abscissa step was set to four.

To assess to what extent the distances obtained by ^19^F Mims ENDOR translate to the liquid state, geometry optimisations of the spin‐labelled ILs **[ILC4][NTf_2_]** and **[ILC8][NTf_2_]** by DFT methods were performed (see Supporting Information S4 for details). The calculated structure in Figure 4c shows a representative low‐energy conformer of the IL solution state structure. The distances determined by DFT agree well with those obtained experimentally by the ^19^F Mims ENDOR experiments and support the existence of the spin‐labelled ILs in a globular and compact nanodomain. The isolated ion pair cation–anion interaction within the spin‐labelled ILs was further probed at room temperature by ^19^F PRE NMR, thereby providing a method of observing ionic association in the liquid state. Enhanced nuclear relaxation rates are observed for all magnetically active nuclei in the vicinity of an unpaired electron due to through‐space dipolar, Fermi contact, and Curie‐spin relaxation contributions. For the ^19^F nuclei of the spin‐labelled ILs, where the *g*‐anisotropy and Fermi contact contribution are negligible, and the distance between the unpaired electron and the ^19^F nucleus is greater than 3 Å, the enhanced spin–spin relaxation rate (*Γ*_2_) can be described by the Solomon‐Bloembergen equation:

(2) $$\;\Gamma_{2}=\frac{1}{15}\;{\left(\frac{\mu_{0}}{4\pi }\right)}^{2}{\gamma_{F}}^{2}g^{2}{\mu_{B}}^{2}S\left(S+1\right)r^{-6}\left(4\tau_{c}+\frac{3\tau_{c}}{1+{\left(\omega_{F}\tau_{c}\right)}^{2}\;}\right)$$

where *µ_0_* is the vacuum permeability, *γ*_F_ is the ^19^F gyromagnetic ratio, *g* is the isotropic electronic *g*‐factor, *µ_B_* is the Bohr magneton and *S* is the electronic spin quantum number.^[^ ^67^, ^85^, ^86^ ^]^ *Γ*_2_ is then dependent on the Larmor frequency of the ^19^F nucleus (ω_F_), the overall molecular rotational correlation time (*τ*_c_), containing contributions from the electronic relaxation time (*τ*_s_) and the rotational correlation time (*τ*_r_), and crucially the distance (*r*) between the unpaired electron and the ^19^F nucleus. For unpaired electronic spin centres with long relaxation times at room temperature (e.g., nitroxides), *τ*_s_ can be neglected and *τ*_c_ = *τ*_r_.^[^ ^68^ ^]^

Samples of the diamagnetic reference, [C_8_C_1_im][NTf_2_], and the spin‐labelled ILs, **[ILC4][NTf_2_]** and **[ILC8][NTf_2_]**, in both CD_2_Cl_2_ and CD_3_CN solvent matrices each exhibited a single ^19^F resonance. This corresponds to the averaged trifluoromethyl moieties of the [NTf_2_]^−^ anion, with remarkably similar chemical shift values (Table 2). The values of *τ*_r_ were approximated via simulation of the room temperature CW EPR spectra of the spin‐labelled ionic liquids **[ILC4][NTf_2_]** and **[ILC8][NTf_2_]** in both CD_2_Cl_2_ and CD_3_CN (Figure S47). The determined values, *τ*_r _= 0.1–0.12 ns, correspond to the expected sub‐nanosecond fast‐motion regime, demonstrating that there are no local viscosity effects owing to the spin‐labelled ILs. Moreover, a small value of *τ*_r_ is expected for dilute solutes of low molecular weight systems in molecular solvents of low viscosity (at room temperature the measured viscosity of CD_2_Cl_2_ and CD_3_CN is 0.413 and 0.369 mPa s, respectively). The *Γ*_2_ rates were then determined from the Carr—Purcell—Meiboom–Gill (CPMG) spin‐spin relaxation data to obtain the solution averaged nitroxide‐^19^F distances for the spin‐labelled ILs, shown in Table 2 (see Supporting Information S6 for details).^[^ ^64^ ^]^ For both spin‐labelled ILs, the PRE‐derived values in CD_2_Cl_2_ corresponded approximately to the longer nitroxide‐^19^F distances determined from ^19^F Mims ENDOR. This shows that flash‐freezing of the spin‐labelled ILs prior to pulse EPR measurements captures a representative picture of the cation–anion interactions in the solution state. The drawback of PRE NMR compared to ENDOR is that only one solution‐averaged distance is obtained if only one ^19^F resonance is present at room temperature, and thus the calculated value represents the sum of each dynamic nitroxide‐^19^F intramolecular interaction per spin‐labelled IL. Samples measured in CD_3_CN exhibited considerably longer PRE‐derived distances (Table 2). Noting that CD_3_CN and CD_2_Cl_2_ have room‐temperature dielectric constants of 37.5 and 8.93, respectively, the longer distances in CD_3_CN demonstrate that solvents with a higher dielectric constant have a greater capacity to separate the cation–anion interactions, as previously suggested by DFT calculations^[^ ^87^ ^]^ and observed by conductivity measurements and vibrational spectroscopy.^[^ ^88^, ^89^, ^90^ ^]^ PRE NMR therefore represents an alternative approach to determining the room temperature strength of long‐lived ionic associations by NMR, where nuclear Overhauser effect (NOE) experiments, or careful examination of chemical shifts under titration, or of pseudocontact shifts in the case of lanthanide‐based ILs, have thus far been employed exclusively.^[^ ^88^, ^91^, ^92^, ^93^ ^]^

**Table 2:** PRE NMR‐derived nitroxide‐anion distances within spin‐labelled ILs in solution. Measured chemical shifts, relaxation enhancement rates, *Γ*_2_, by ^19^F PRE‐NMR and corresponding solution averaged nitroxide‐^19^F distances determined using Equation 2 for the spin‐labelled ILs in CD_2_Cl_2_ and CD_3_CN.

Solvent matrix	IL	Chemical shift (ppm)	*Γ*_2_ (s^−1^)	r (Å)
CD_2_Cl_2_	[C_8_C_1_im][NTf_2_]	−79.55	–	–
CD_2_Cl_2_	**[ILC4][NTf_2_]**	−79.21	19.4	8.8
CD_2_Cl_2_	**[ILC8][NTf_2_]**	−79.38	7.9	10.3
CD_3_CN	[C_8_C_1_im][NTf_2_]	−80.20	–	–
CD_3_CN	**[ILC4][NTf_2_]**	−80.11	1.4	13.5
CD_3_CN	**[ILC8][NTf_2_]**	−80.16	1.4	13.5

### Nitroxide‐Anion Interactions are Independent of the Cation

**Figure 5:**
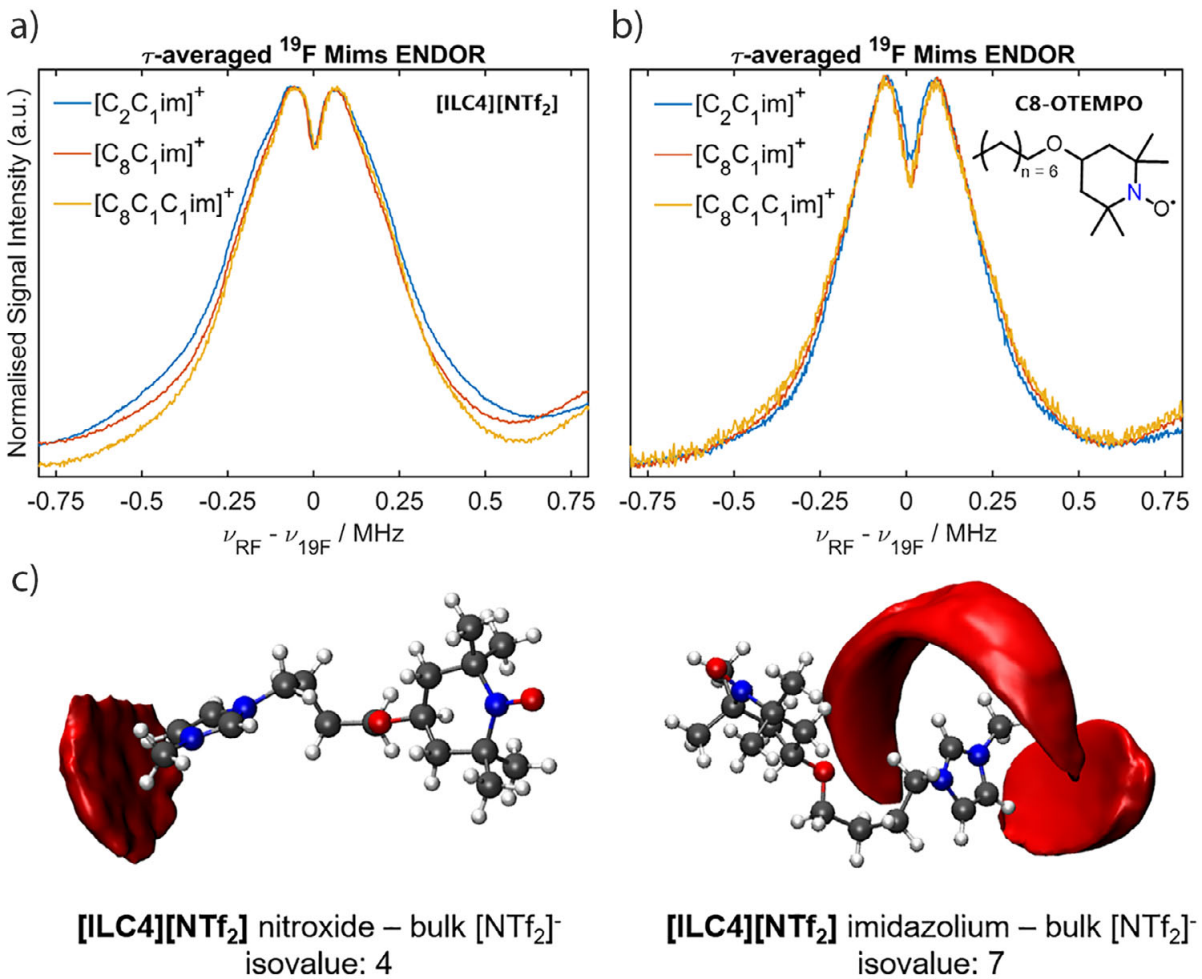
^19^F ENDOR measurements of spin‐labelled ILs and spin probes in ILs with cations of different chain lengths. Q‐band, *τ*‐averaged, ^19^F Mims ENDOR experiments of a) **[ILC4][NTf_2_]** (0.2 mM) dissolved in [C_2_C_1_im][NTf_2_] (blue), [C_8_C_1_im][NTf_2_] (orange), and [C_8_C_1_C_1_im][NTf_2_] (yellow) b) **C8‐OTEMPO** (0.2 mM, chemical structure, and abbreviation, inset) dissolved in [C_2_C_1_im][NTf_2_] (blue), [C_8_C_1_im][NTf_2_] (orange), and [C_8_C_1_C_1_im][NTf_2_] (yellow). c) SDFs (red) and corresponding isovalues showing the directionality of the Molecular Dynamics (MD) calculated bulk [NTf_2_]^−^ anion interactions in **[ILC4][NTf_2_]** with the (*left*) nitroxide spin centre, (*right*) imidazolium ring (red: O, blue: N, gray: C, white: H).

With a detailed understanding of the molecular interactions that occur within isolated spin‐labelled IL ion pairs, we turned to investigating the solute–solvent interaction sphere of **[ILC4][NTf_2_]** and **[ILC8][NTf_2_]** dissolved in different IL systems (rather than traditional solvents), namely [C_2_C_1_im][NTf_2_], [C_8_C_1_im][NTf_2_], and [C_8_C_1_C_1_im][NTf_2_]. While measurements with [C_2_C_1_C_1_im][NTf_2_] were attempted, this IL undergoes a phase transition from liquid to solid around room temperature and was therefore not investigated further (see Supporting Information S3). By monitoring the dipolar interaction of the spin‐labelled IL with the ^19^F nuclei of the [NTf_2_]^−^ anion, we can monitor changes in the IL solvation nanostructure as the IL environment is altered. The ^19^F Mims ENDOR experiments of **[ILC4][NTf_2_]** dissolved in these ILs showed a broad distribution of dipolar coupling interactions. However, there were no major changes in these interactions between the different IL matrices (Figure 5a), which were broadened compared to the isolated spin‐labelled IL ion pairs in molecular solvents (Figure 4a). A similar set of results was obtained for **[ILC8][NTf_2_]** (Figure S14).

Determination of the experimental hyperfine coupling value from the spectral splitting, *T*_read_, allows an upper distance limit for the anionic solvation shell to be obtained. The anionic solvation shell surrounding the spin centree is corroborated by MD results taking 50 [ILC4]^+^ spin‐labelled cations in 450 [C_8_C_1_im]^+^ cations and 500 [NTf_2_]^−^ anions (details in Supporting Information S4). The spatial distribution functions (SDFs) show a preferential interaction of the bulk [NTf_2_]^−^ anions with both the [C_8_C_1_im]^+^ and the spin‐labelled IL imidazolium ring via an electrostatic interaction. No short‐range interactions between the [NTf_2_]^−^ anion and the nitroxide spin centre are observed (Figure 5c). Therefore, from both the MD simulations and the ENDOR spectra, we conclude that the anionic solvation shell is independent of the IL cation structure, with an anionic solvation shell size with respect to the electron spin of ca. 9.5 nm. Having shown that the [NTf_2_]^−^ anion's interaction with the spin‐labelled cations is not dependent on the IL cation structure, we sought to determine if the same results are observed for uncharged spin probes. We therefore repeated the ^19^F Mims ENDOR measurements using the synthesised uncharged spin probe **C8‐OTEMPO** (synthetic details available in Supporting Information S1), dissolved in the same ILs (Figure 5b). The anionic interactions with the spin probe are again the same for all IL cations. The observed couplings are less broad compared to the spin‐labelled ILs, due to the lack of an intramolecular ^19^F coupling from an associated [NTf_2_]^−^ anion with the spin probe. Experiments in the same IL systems using the **TEMPOL** spin probe gave similar results (Figure S16). Thus, the spin centre of each solute (spin‐labelled ILs, **C8‐OTEMPO** and **TEMPOL**) exhibits primarily long‐range interactions with the IL anion.

### Nitroxides Preferentially Associate with the Imidazolium Ring

**Figure 6:**
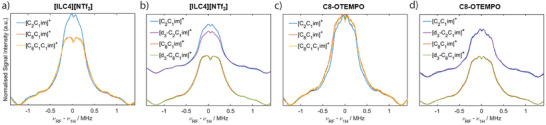
^1^H ENDOR measurements of spin‐labelled ILs and spin probes in ILs with cations of different chain lengths and perdeuteration of the imidazolium ring. Q‐band, *τ*‐averaged, ^1^H Mims ENDOR experiments of [ILC4][NTf_2_] (0.2 mM) in a) [C_2_C_1_im][NTf_2_] (blue), [C_8_C_1_im][NTf_2_] (orange), and [C_8_C_1_C_1_im][NTf_2_] (yellow) ILs; b) [C_2_C_1_im][NTf_2_] (blue), [d_3_‐C_2_C_1_im][NTf_2_] (purple), [C_8_C_1_im][NTf_2_] (orange), and [d_3_‐C_8_C_1_im][NTf_2_] (green). Q‐band, *τ*‐averaged, ^1^H Mims ENDOR experiments of C8‐OTEMPO (0.2 mM) in c) [C_2_C_1_im][NTf_2_] (blue), [C_8_C_1_im][NTf_2_] (orange), and [C_8_C_1_C_1_im][NTf_2_] (yellow); d) [C_2_C_1_im][NTf_2_] (blue), [d_3_‐C_2_C_1_im][NTf_2_] (purple), [C_8_C_1_im][NTf_2_] (orange), and [d_3_‐C_8_C_1_im][NTf_2_] (green).

Q‐band ^1^H Mims ENDOR spectra were measured to understand the local ^1^H spin density qualitatively. Considering **[ILC4][NTf_2_]** dissolved in [C_2_C_1_im][NTf_2_], [C_8_C_1_im][NTf_2_] and [C_8_C_1_C_1_im][NTf_2_], an additional weak coupling distribution is observed in the [C_2_C_1_im][NTf_2_] ^1^H Mims ENDOR spectrum, compared to the [C_8_C_1_im][NTf_2_] and [C_8_C_1_C_1_im][NTf_2_] spectra (Figure 6a). This is postulated to be due to the nitroxide spin‐centre coupling to hydrogens located in the 2^nd^ solvation shell of [C_2_C_1_im][NTf_2_], whose smaller molecular volume can pack closer to the spin centre compared to ILs with long alkyl chains. To probe interactions with the cations further, we perdeuterated the cations’ imidazolium ring to give [d_3_‐C_2_C_1_im][NTf_2_] and [d_3_‐C_8_C_1_im][NTf_2_] ILs (synthetic details in Supporting Information S1). **[ILC4][NTf_2_]** dissolved in the perdeuterated ILs showed a marginally weaker distribution of ^1^H couplings compared to the non‐deuterated samples, most evident in the [d_3_‐C_2_C_1_im][NTf_2_] sample (Figure 6b).

The same results are observed for the longer chain spin‐labelled IL **[ILC8][NTf_2_]** (Figure S19). For the spin probe **C8‐OTEMPO** dissolved in [C_2_C_1_im][NTf_2_], a similar ^1^H ENDOR spectrum is recorded as for the spin‐labelled ILs, however the [C_8_C_1_im][NTf_2_] and [C_8_C_1_C_1_im][NTf_2_] samples show a change in the ^1^H environment that is different to the change observed in the spin‐labelled ILs (Figure 6c). **C8‐OTEMPO** dissolved in perdeuterated ILs showed a less evident change in ^1^H couplings compared to the non‐deuterated samples (Figure 6d). These results suggest that both the spin‐labelled ILs and spin probes experience small changes in the ^1^H solvation environment between ILs beyond the first coordination sphere where the interactions are expected to be weak (>1 MHz). Recently reported intermolecular hyperfine relaxation‐induced dipolar modulation enhancement (ih‐RIDME) experiments may be a useful tool to quantitatively describe the ^1^H environment of different spin probes dissolved in ILs.^[^ ^94^ ^]^

**Figure 7:**
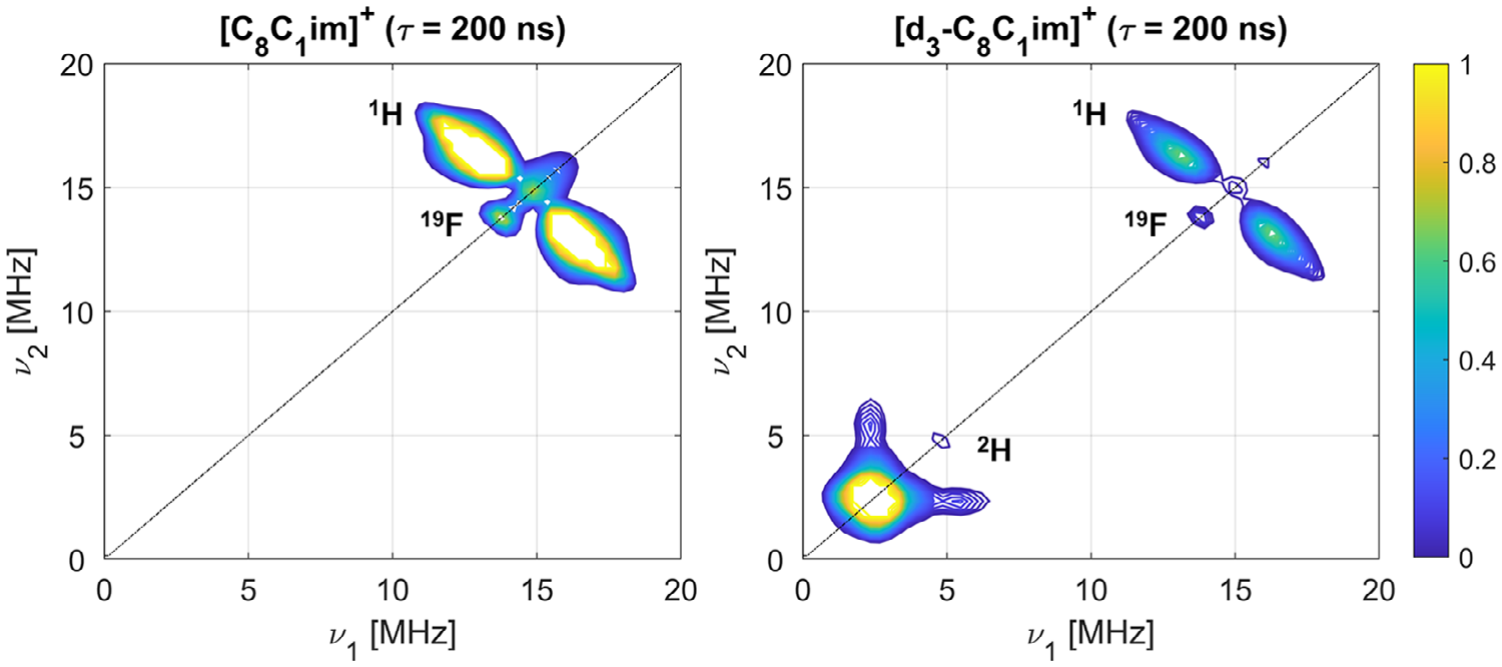
X‐band 4‐pulse HYSCORE measurements. Spectra recorded with *τ* = 200 ns for **[ILC4][NTf_2_]** (0.2 mM) in [C_8_C_1_][NTf_2_] (left) and [d_3_‐C_8_C_1_][NTf_2_] (right) showing the weak‐coupling (+,+) quadrant (details in Supporting Information S3).

X‐band 4‐pulse hyperfine sublevel correlation (HYSCORE) experiments (experimental details in Supporting Information S2) also showed only weak couplings at the ^1^H Larmor frequency, with no appreciable difference recorded between the ILs. A broad ^1^H coupling distribution of ca. |2*T* + a_iso_| = 8 MHz can be assigned from the HYSCORE spectrum in all cases. HYSCORE measurements of the spin‐labelled ILs dissolved in perdeuterated ILs revealed an additional feature, corresponding to a weakly coupled single quantum (SQ) peak centred at the ^2^H Larmor frequency alongside a weaker double quantum (DQ) peak, stemming from the small, positive quadrupolar moment of ^2^H (Figure 7). 6‐pulse HYSCORE measurements, which suffer less from multi‐nuclear suppression effects,^[^ ^95^ ^]^ were almost indistinguishable (Figure S22). 4‐pulse HYSCORE measurements under the same conditions using the spin‐probe **C8‐OTEMPO** showed similar ^1^H coupling distributions and the appearance of ^2^H signals in the perdeuterated samples (Figure S23). Together with the ENDOR measurements, these results point to the nitroxide spin centre in all samples preferentially interacting with the imidazolium ring hydrogens rather than the alkyl chain hydrogens.

### Weak Hydrogen Bonding is Observed with Nitroxide Solutes

**Figure 8:**
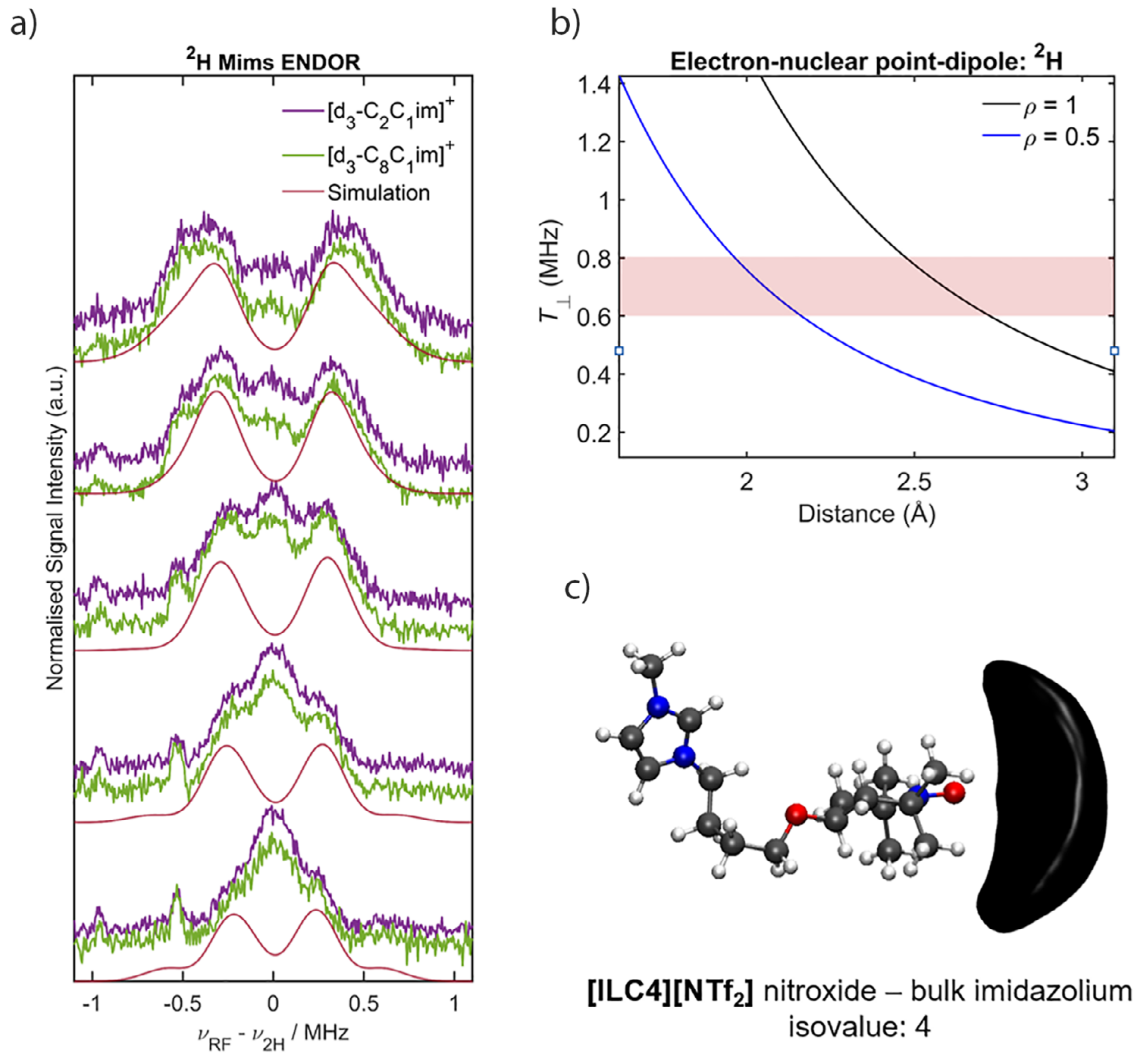
^2^H ENDOR spectra suggest a weak hydrogen‐bonding interaction with the imidazolium ring. a) Q‐band ^2^H Mims ENDOR experiments of **[ILC4][NTf_2_]** (0.2 mM) in [d_3_‐C_2_C_1_im][NTf_2_] (purple) and [d_3_‐C_8_C_1_im][NTf_2_] (green), and corresponding simulations for different values of *τ* (see also Figure S24) for a summed simulation with *T* = 0.6, 0.7 and 0.8 MHz (see also Figure S24), assuming purely dipolar coupling (details in Supporting Information S3). b) Calculated electron‐^2^H distances under a point dipole model for different values of the spin density (*ρ*) on the oxygen atom of the nitroxide moiety for the range of *T* values that gave reasonable fits to the experimental data (red shading, see also Figure S26). c) SDFs (black) and corresponding isovalue showing the directionality of the MD calculated bulk imidazolium ring interactions in **[ILC4][NTf_2_]** with the nitroxide spin centre (red: O, blue: N, gray: C, white: H).

To assign the ^2^H peaks stemming from the perdeuterated imidazolium ring observed in the X‐band HYSCORE measurements, we probed the ^2^H nuclei directly using Mims ENDOR (Supporting Information S2 for details). Nitroxide spin centres are known hydrogen bond acceptors.^[^ ^96^, ^97^, ^98^ ^]^ The hydrogen atoms of the imidazolium ring have been shown to be the most accessible protons for hydrogen bonding in imidazolium‐based ILs.^[^ ^70^, ^88^, ^99^ ^]^ Therefore with ^2^H Mims ENDOR, we aimed to determine if the nitroxide spin centre is interacting with the hydrogen atoms of the imidazolium ring, and if the cation structure has any effect on this interaction. For the spin‐labelled IL **[ILC4][NTf_2_]**, a coupling of the nitroxide spin centre to ^2^H nuclei is observed that does not change in magnitude between [d_3_‐C_2_C_1_im][NTf_2_] and [d_3_‐C_8_C_1_im][NTf_2_] (Figure 8a). Simulations of the individual ^2^H ENDOR data sets were performed excluding the matrix peak at the ^2^H Larmor frequency and reasonable fits to the data were obtained with *T* = 0.6–0.8 MHz (Figure S24), assuming a purely dipolar interaction (i.e., ***A*** = [‐*T*, ‐*T*, 2*T*]).^[^ ^100^ ^]^ The inclusion of nuclear quadrupole parameters had no effect on the fitting of the ENDOR simulations. This is likely due to the small magnitude of both the quadrupole coupling constant, *e* ^2^ *Qq*/*h* ≈ 150 – 200 KHz, and asymmetry parameter, *η* < 0.1, expected for rigid sp^2^ hybridised C─D bonds.^[^ ^61^ ^]^ In conjunction with Q‐band ^1^H Davies ENDOR difference spectra of **TEMPOL** in protonated and deuterated ILs (Figure S25), the observed hyperfine coupling interactions can be attributed to weak hydrogen bonding of the nitroxide spin centre with all three of the ^2^H atoms of the imidazolium ring. The inter‐atomic D⋯O• lengths (2.0–2.7 Å) derived from the hyperfine couplings are approximate as they depend on the delocalisation of the electronic spin density *ρ* (see Figure 8b, and further details in Figure S26). Similar ^2^H Mims ENDOR spectra were observed for the spin labelled IL **[ILC8][NTf_2_]**, and the spin probe samples, **C8‐OTEMPO** and **TEMPOL** (Figure S28 and S29).

**Figure 9:**
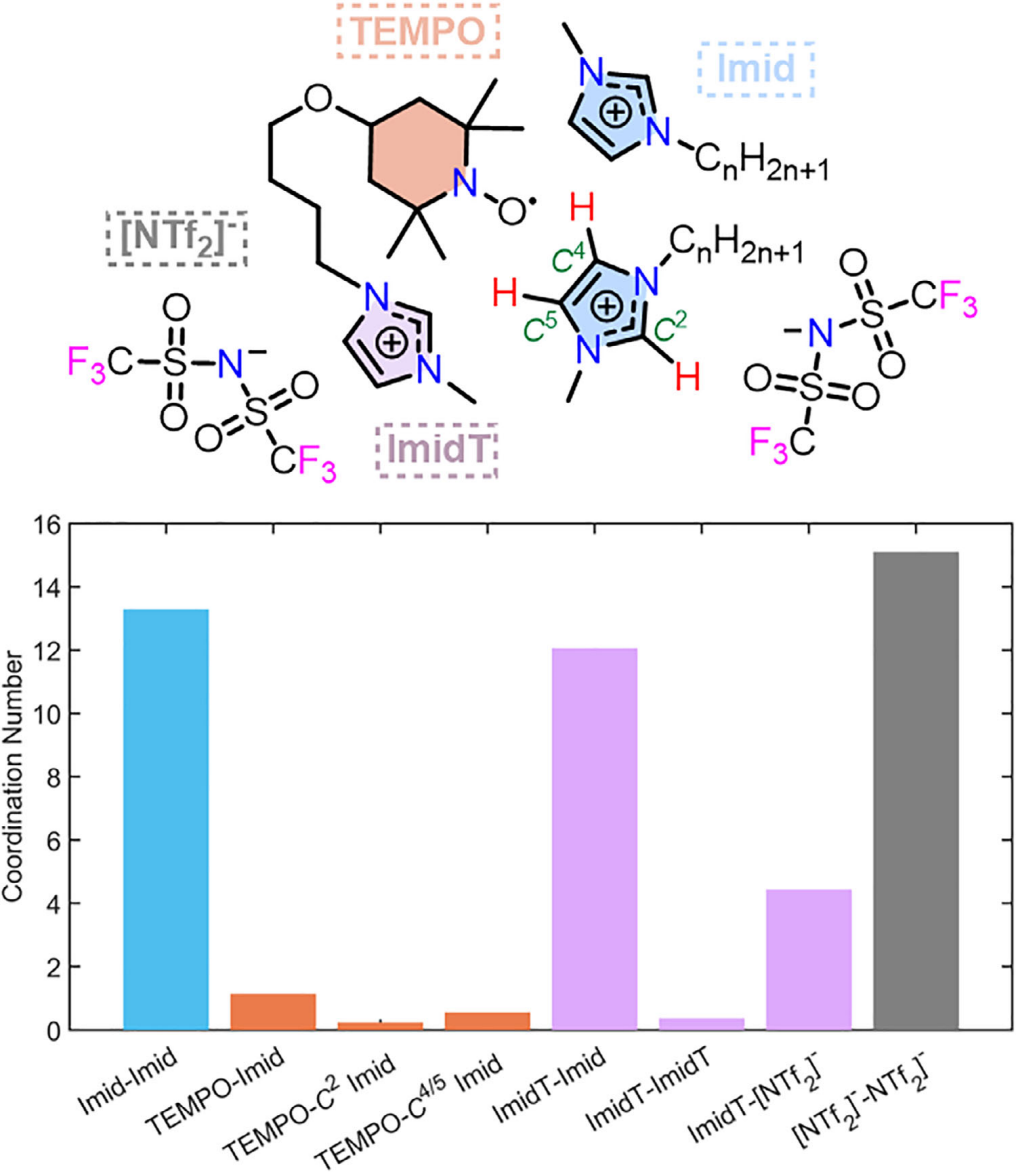
Proposed nanostructuring in ILs with nitroxide solutes from pulse EPR measurements and MD simulations. **(Top)** Schematic of suggested favourable interactions of nitroxide solutes (**[ILC4][NTf_2_]** shown) with imidazolium‐based cations and [NTf_2_]^−^ anions. **(Bottom)** Bar chart of select interactions between different sites from the MD simulations, showing the coordination number determined from the integral before the 1^st^ minima of the individual radial distribution functions (RDFs) (see Supporting Information S4 for details).

The hydrogen bonding interaction determined from the ENDOR experiments is further supported by MD simulations of **[ILC4][NTf_2_]** dissolved in [C_8_C_1_im][NTf_2_] (Figure 8c). Our MD results (Figure 9) show that the highest coordination numbers are present in systems with interactions predominantly driven by dispersion forces and clustering, as shown previously for nitroxide‐based solutes.^[^ ^101^ ^]^ This may be partially due to these solvation shells being slightly larger (Figure S35), however this does not explain the entirety of the relatively large coordination numbers. Previous results have demonstrated that the most energetically favorable ion pair orientations are those where the cations are stacked on top of each other and the anion lies off‐plane from the cations, thus stabilising the charge.^[^ ^29^ ^]^ Interestingly, the MD simulations suggest a preferential interaction of the nitroxide spin centre with the *C* ^4^/*C* ^5^ protons of the imidazolium ring over the *C* ^2^ proton, possibly due to a cooperative bonding effect, as well as the higher surface area for the interaction.

Crucially, the micelle‐like IL solvation structure previously reported,^[^ ^43^ ^]^ where nitroxide solutes have been proposed to preferentially associate with the non‐polar alkyl chains of the IL cation, is not supported in this study, neither experimentally nor computationally. Rather, it is the polar imidazolium ring that is found to associate favorably with the solute, for both nitroxide spin probes and the spin‐labelled ILs (Figure 9). Further, dynamic light scattering (DLS) experiments of **[ILC4][NTf_2_]** dissolved in [d_3_‐C_8_C_1_im][NTf_2_] did not show any evidence of micellar nanostructuring around the nitroxide solutes at the concentrations used for pulse EPR measurements (see Supporting Information S7). Thus, the picture that emerges is a less ordered, or “softer”, solute–solvent nanostructure (Figure 9) than previously hypothesised.

## Conclusion

We have applied a combination of advanced pulse hyperfine EPR techniques, ^19^F PRE NMR, DFT calculations and MD simulations in order to determine the solute–solvent interaction sphere and molecular level nanostructuring of imidazolium‐based ILs. A family of spin probes and nitroxide spin‐labelled ILs were synthesised, featuring targeted isotopic labelling, such that both the intra‐ and intermolecular non‐covalent interactions could be measured by magnetic resonance techniques. The solvation of nitroxide radicals has previously been associated with the formation of micelle‐like structures around the solute. Instead, our ENDOR results, supported by MD simulations, reveal that the solute–solvent nanostructures in imidazolium‐based ILs do not form a micelle‐like structure around nitroxide solutes, but rather are found to associate with the polar domains of the imidazolium cation through weak hydrogen bonding with the ring protons. The consistent long‐range solvation of the nitroxide‐based spin centres by the ^19^F nuclei of the anionic constituent of the ILs, independent of the cationic component, further support this revised picture of IL nanostructuring. Our results thus challenge current interpretations of nitroxide solute–solvent interactions in ILs and could have implications for the design and use of next‐generation, task‐specific IL systems. Further, the methodologies presented herein could be extended to characterise non‐covalent interactions in similar soft matter systems.

## Supporting Information

The authors have cited additional references within the Supporting Information.^[^ ^18^, ^45^, ^74^, ^102^, ^103^, ^104^, ^105^, ^106^, ^107^, ^108^, ^109^, ^110^, ^111^, ^112^, ^113^, ^114^, ^115^, ^116^, ^117^, ^118^, ^119^, ^120^, ^121^ ^]^ The raw data supporting this study, and the MATLAB‐based data analysis and simulation routines, are available from the Imperial Research Data repository DOI: 10.14469/hpc/14504.

## Conflict of Interests

The authors declare no conflict of interest.

## Supporting information



Supporting Information

## Data Availability

The data that support the findings of this study are available from the corresponding author upon reasonable request.
