# Supplementary material for: Revealing the Nature of Non‐Covalent Interactions in Ionic Liquids by Combined Pulse EPR and 19F NMR Spectroscopy
Source: Angew Chem Int Ed Engl. 2025 May 10;64(28):e202504882. doi: 10.1002/anie.202504882 (PMC12232894; doi:10.1002/anie.202504882)
Supplement: Supplementary file 1 — Supporting Information [file ANIE-64-e202504882-s001.pdf]

Supporting Information for

Revealing the Nature of Non-Covalent Interactions in Ionic Liquids by Combined Pulsed EPR and ^{19}F NMR Spectroscopy

Ciarán J. Rogers,[a,b]† Spyridon Koutsoukos,[a]† Jana Eisermann,[a,b,c] Luke Wylie,[d] Gavin J. Smith,[a] Tom Welton*[a] and Maxie M. Roessler*[a,b]

[a] Department of Chemistry, Imperial College London, Molecular Sciences Research Hub, London W12 0BZ, United Kingdom.

[b] Centre for Pulse EPR Spectroscopy (PEPR), Imperial College London, Molecular Sciences Research Hub, London W12 0BZ, United Kingdom.

[c] Department of Chemistry, University of Stuttgart, Institute of Physical Chemistry, Pfaffenwaldring 55, 70569 Stuttgart, Germany.

[d] Mulliken Center for Theoretical Chemistry, Institute for Physical and Theoretical Chemistry, University of Bonn, Beringstr. 4, D-53115 Bonn, Germany.

E-mail: m.roessler@imperial.ac.uk, t.welton@imperial.ac.uk

† These authors contributed equally to this work.

Contents

S.1 Synthetic procedures 3
S.2 Experimental methods 14
S.3 Pulse EPR data 16
S.4 Computational methods 40
S.5 Room temperature continuous wave EPR spectra 47
S.6 ^{19}F paramagnetic relaxation enhancement (PRE) measurements 48
S.7 Dynamic Light Scattering (DLS) 50
S.8 Optimised DFT coordinates 51
References 56

S.1 Synthetic procedures

All chemicals were purchased from VWR or Sigma Aldrich, unless stated otherwise, and were purified using standard techniques. The solvents used for the synthesis were HPLC grade and used without further purification. All glassware was washed with absolute ethanol, followed by washes with decon-90 and de-ionised water before use.

Synthesis of Ionic Liquids (ILs)

Synthesis of non-deuterated [BF_4], [NTf_2] and [FSI] based imidazolium ILs

The synthesis of the ILs with [BF_4], [NTf_2] and [FSI] anions consists of two steps, the synthesis of the alkyl-imidazolium halide salt, followed by the anion metathesis. These procedures have been described in detail in previous publications.[1,2]

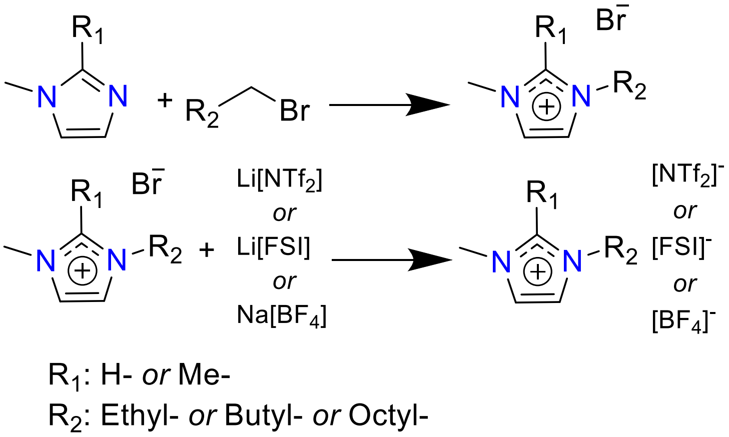

Scheme S1. Synthetic route for the formation of [BF_4], [NTf_2] and [FSI] based imidazolium ILs.

The bromoalkanes were washed with conc. sulphuric acid until the acid layer appeared colourless, followed by washes with saturated sodium bicarbonate solution and then de-ionised water, until the aqueous phase appeared neutral. The bromoalkene phase was dried over magnesium sulphate and distilled. 1-methylimidazole was stirred overnight over potassium hydroxide and distilled *in vacuo*. 1,2-dimethylimidazole was recrystallised from toluene and dried *in vacuo*. 1.2 eq. of bromoalkene was added dropwise to a stirring solution of 1 eq. of 1-methylimidazole or 1,2-dimethylimidazole in ethyl acetate in an ice bath. Once addition was complete, the mixture was allowed to reach room temperature and then was heated for up to a week at 40 °C, monitored by ^{1}H NMR. Upon reaction completion, the solvent was removed with a cannula and the halide salt was dried *in vacuo* followed by recrystallisation with acetonitrile – ethyl acetate and then further drying *in vacuo*. All halide salts were stored under dry nitrogen atmosphere and were further dried before use.

[C_2C_1im]Br. 1-bromoethane (20 mL, 268 mmol, 1.2 eq.) was added to a stirring solution of purified 1-methylimidazole (17.8 mL, 223.3 mmol, 1 eq.) in ethyl acetate (100 mL). Upon completion of the

reaction, the salt was dried overnight on the Schlenk line to yield a white solid (37 g, 194.3 mmol, 87% yield).

^{1}H NMR (400 MHz, $CDCl_3$, ppm): *δ* = 10.21 (s, 1H, imC(2)**H**), 7.59-7.54 (m, 2H, imC(4,5)**H**), 4.34 (q, J = 7.36 Hz, 2H, ethyl – C(1)$\mathbf{H_2}$), 4.04 (s, 3H, methyl – C$\mathbf{H_3}$), 1.53 (t, J=7.37 Hz, 3H, ethyl – C$\mathbf{H_3}$). ^{13}C NMR (101 MHz, $CDCl_3$, ppm): *δ* = 136.88 (imC(2)), 123.7 (imC(4)), 121.98 (imC(5)), 45.24 (ethyl – C(1)), 36.67 (methyl – C(1)), 15.67 (ethyl – C(2)). MS (ES): *m/z* calcd for M+ 111.1; found: 111.1 (100%).

[C_4C_1im]Br. 1-bromobutane (10 mL, 92.7 mmol, 1.2 eq.) was added to a stirring solution of purified 1-methylimidazole (6.9 mL, 84.3 mmol, 1 eq.) in ethyl acetate (50 mL). Upon completion of the reaction the salt was dried on the Schlenk line overnight to yield a white solid (14.6 g, 66.6 mmol, 79% yield).

^{1}H NMR (400 MHz, $CDCl_3$, ppm): *δ* = 10.38 (s, 1H, imC(2)**H**), 7.56 (t, J = 1.8 Hz, 1H, imC(4)**H**), 7.44 (t, J = 1.8 Hz, 1H, imC(5)**H**), 4.31 (t, J = 7.4 Hz, 2H, butyl-C(1)$\mathbf{H_2}$), 4.1 (s, 1H, methyl-C(1)$\mathbf{H_3}$), 1.88 (dt, J = 8.2, 6.7 Hz, 2H, butyl-C(2)$\mathbf{H_2}$), 1.35 (m, 2H, butyl-C(3)$\mathbf{H_2}$), 0.93 (t, J = 7.4 Hz, 3H, butyl-C(4)$\mathbf{H_3}$). ^{13}C NMR (101 MHz, $CDCl_3$, ppm): *δ* = 136.0 (imC(2)), 123.7 & 121.4 (imC(4,5)), 49.3 (methylC(1)), 36.9 (butyl-C(1)), 33.0 (butyl-C(2)), 20.4 (butyl-C(3)), 13.5 (butyl-(C(4)). MS (ES): *m/z* calcd for M+ 139.1; found: 139.1 (100%). MS (ES): *m/z* calcd for M- 80.9; found: 80.9 (100%).

[C_8C_1im]Br. 1-bromooctane (10 mL, 57.5 mmol, 1.2 eq.) was added to a stirring solution of purified 1-methylimidazole (3.9 mL, 47.9 mmol, 1 eq.) in ethyl acetate (50 mL). Upor completion of the reaction the salt was dried overnight on the Schlenk line to yield a viscous colourless liquid (10.9 g, 39.8 mmol, 83% yield).

^{1}H NMR (400 MHz, $CDCl_3$, ppm): *δ* = 9.21 (s, 1H, imC(2)**H**), 7.81 (t, J = 1.8 Hz, 1H, imC(4)**H**), 7.73 (t, J = 1.8 Hz, 1H, imC(5)**H**), 4.16 (t, J = 7.2 Hz, 2H, octyl-C(1)$\mathbf{H_2}$), 3.86 (s, 1H, methyl-C(1)$\mathbf{H_3}$), 1.77 (m, 2H, octyl-C(2)$\mathbf{H_2}$), 1.25 (m, 12H, octyl-C(3-7)$\mathbf{H_2}$), 0.85 (t, J = 7.4 Hz, 3H, octyl-C(8)$\mathbf{H_3}$). ^{13}C NMR (101 MHz, $CDCl_3$, ppm): *δ* = 136.5 (imC(2)), 123.6 & 122.2 (imC(4,5)), 48.7 (methyl-C(1)), 35.7 (octyl-C(1)), 31.1 (octyl-C(2)), 29.4 (octyl-C(3)), 28.5 (octyl-(C(4)), 28.3 (octyl-C(5)), 25.5 (octyl-C(6)), 22.0 (octyl-C(7)), 13.9 (octyl-C(8)). MS (ES): *m/z* calcd for M+ 195.2; found: 195.2 (100%). MS (ES): *m/z* calcd for M- 80.9; found: 80.9 (100%).

[$C_8C_1C_1$im]Br. 1-bromooctane (10 mL, 57.5 mmol, 1.2 eq.) was added to a stirring solution of purified 1,2-dimethylimidazole (4.6 g, 47.9 mmol, 1 eq.) in ethyl acetate (50 mL). Upon completion of the reaction the salt was dried overnight on the Schlenk line to yield a white solid (11.4 g, 39.2 mmol, 82% yield).

^{1}H NMR (400 MHz, $CDCl_3$, ppm): *δ* = 7.72 (d, J = 2.1 Hz, 1H, imC(4)**H**), 7.43 (t, J = 2.1 Hz, 1H, imC(5)**H**), 4.17 (t, J = 7.3 Hz, 2H, octyl-C(1)$\mathbf{H_2}$), 4.01 (s, 1H, methyl-C(1)$\mathbf{H_3}$), 2.79 (s, 1H, im-C$\mathbf{H_3}$), 1.80 (m, 2H, octyl-C(2)$\mathbf{H_2}$), 1.26 (m, 12H, octyl-C(3-7)$\mathbf{H_2}$), 0.84 (t, J = 7.4 Hz, 3H, octyl-C(8)$\mathbf{H_3}$). ^{13}C NMR (101 MHz, $CDCl_3$, ppm): *δ* = 143.9 (imC(2)), 123.2 & 121.1 (imC(4,5)), 49.2 (methyl-C(1)), 36.4 (octyl-C(1)), 31.8 (octyl-C(2)), 29.9 (octyl-C(3)), 29.1 (octyl-(C(4,5)), 26.5 (octyl-C(6)), 22.7 (octyl-C(7)), 14.1 (octyl-C(8)), 11.2 (imC(2')). MS (ES): *m/z* calcd for M+ 209.2; found: 209.2 (100%). MS (ES): *m/z* calcd for M- 80.9; found: 80.9 (100%).

For the $[NTf_2]$ and [FSI] and $[BF_4]$ ionic liquids, 1.1 eq. of lithium bis(trifluoromethylsulfonyl)imide or lithium bis(fluorosulfonyl)imide or sodium tetrafluoroborate were added to a stirring solution of 1 eq. of the corresponding alkylimidazolium bromide in dichloromethane and was left stirring for 24 h ambient conditions. The resulting dispersion was filtered to remove the precipitated lithium or sodium bromide salt. The organic phase was washed with de-ionised water until the aqueous phase was negative by silver nitrate test. Dichloromethane was dried over magnesium sulphate and evaporated *in vacuo*. The IL was further dried overnight *in vacuo*.

$[C_2C_1im][NTf_2]$. Lithium bis(trifluoromethylsulfonyl)imide (16.5 g, 57.5 mmol, 1.1 eq.) was added to a stirring solution of $[C_2C_1im]Br$ (10 g, 52.3 mmol, 1 eq.) in DCM (50 mL). After the purification steps, the IL was dried overnight on the Schlenk line to yield a viscous colourless liquid (19.6 g, 50.2 mmol, 96% yield).

^1H NMR (400 MHz, DMSO-d_6, ppm): δ = 9.1 (s, 1H, imC(2)**H**), 7.76 (t, J = 1.78 Hz, 1H, imC(4)**H**)), 7.69 (t, J = 1.87 Hz, 1H, imC(5)**H**), 4.18 (q, J = 7.31 Hz, 2H, ethyl – C(1)$\mathbf{H_2}$), 3.84 (s, 3H, methyl – C$\mathbf{H_3}$), 1.41 (t, J=7.32 Hz, 3H, ethyl – C$\mathbf{H_3}$); ^{13}C NMR (101 MHz, DMSO-d_6, ppm): δ = 136.24 (imC(2)), 124.29 (imC(4)), 121.95 (imC(5)), 119.5 (q, J_{CF} = 321.9 Hz, 2C, NTf_2), 44.14 (ethyl – C(1)), 35.67 (methyl-C(1)), 15.04 (ethyl – C(2)). MS (ES): *m/z* calcd for M+ 111.1; found: 111.1 (100%). MS (ES): *m/z* calcd for M- 279.8; found: 279.8 (100%).

$[C_2C_1im]$[FSI]. Lithium bis(fluorosulfonyl)imide (10.8 g, 57.5 mmol, 1.1 eq.) was added to a stirring solution of $[C_2C_1im]Br$ (10 g, 52.3 mmol, 1 eq.) in DCM (50 mL). After the purification steps, the IL was dried overnight on the Schlenk line to yield a viscous colourless liquid (13.9 g, 47.6 mmol, 91% yield).

^1H NMR (400 MHz, DMSO-d_6, ppm): δ = 9.1 (s, 1H, imC(2)**H**), 7.77 (t, J = 1.91 Hz, 1H, imC(4)**H**)), 7.68 (t, J = 1.85 Hz, 1H, imC(5)**H**), 4.18 (q, J = 7.31 Hz, 2H, ethyl – C(1)$\mathbf{H_2}$), 3.84 (s, 3H, methyl – C$\mathbf{H_3}$), 1.41 (t, J=7.29 Hz, 3H, ethyl – C$\mathbf{H_3}$); ^{13}C NMR (101 MHz, DMSO-d_6, ppm): δ = 136.22 (imC(2)), 123.57 (imC(4)), 121.97 (imC(5)), 44.14 (ethyl – C(1)), 35.69 (methyl – C(1)), 15.08 (ethyl – C(2)); HRMS (ES): *m/z* calcd for M+ 111.0922; found: 111.0924 (100%). HRMS (ES): *m/z* calcd for M- 179.9242; found: 179.9265 (100%).

$[C_2C_1im][BF_4]$. Sodium tetrafluoroborate (6.3 g, 57.5 mmol, 1.1 eq.) was added to a stirring solution of $[C_2C_1im]Br$ (10 g, 52.3 mmol, 1 eq.) in DCM (50 mL). After the purification steps the IL, was dried overnight on the Schlenk line to yield a viscous colourless liquid (8.9 g, 45 mmol, 86% yield).

^1H NMR (400 MHz, DMSO-d_6, ppm): δ = 9.14 (s, 1H, imC(2)**H**), 7.79 (t, J = 1.8 Hz, 1H, imC(4)**H**)), 7.70 (t, J = 1.79 Hz, 1H, imC(5)**H**), 4.19 (q, J = 6.9 Hz, 2H, ethyl – C(1)$\mathbf{H_2}$), 3.84 (s, 3H, methyl – C$\mathbf{H_3}$), 1.42 (t, J=7.90 Hz, 3H, ethyl – C$\mathbf{H_3}$); ^{13}C NMR (101 MHz, DMSO-d_6, ppm): δ = 136.23 (imC(2)), 123.56 (imC(4)), 121.97 (imC(5)), 44.11 (ethyl – C(1)), 35.70 (methyl - C(1)), 15.11 (ethyl – C(2)); HRMS (ES): *m/z* calcd for M+ 111.0922; found: 111.0918 (100%). MS (ES): *m/z* calcd for M- 87.00; found: 86.99 (100%).

$[C_4C_1im][NTf_2]$. Lithium bis(trifluoromethylsulfonyl)imide (5 g, 17.4 mmol, 1.1 eq.) was added to a stirring solution of $[C_4C_1im]Br$ (3.5 g, 15.8 mmol, 1 eq.) in DCM (25 mL). After the purification steps, the

IL was dried overnight on the Schlenk line to yield a viscous colourless liquid (6.1 g, 14.4 mmol, 91% yield).

^{1}H NMR (400 MHz, DMSO-d$_6$, ppm): δ = 9.09 (s, 1H, imC(2)**H**), 7.76 (s, 1H, imC(4)**H**), 7.69 (s, 1H, imC(5)**H**), 4.16 (t, J = 7.2 Hz, 2H, butyl-C(1)**H$_2$**), 3.84 (s, 1H, methyl-C(1)**H$_3$**), 1.76 (dt, J = 9.3, 7.3 Hz, 2H, butyl-C(2)**H$_2$**), 1.26 (m, 2H, butyl-C(3)**H$_2$**), 0.90 (t, J = 7.3 Hz, 3H, butyl-C(4)**H$_3$**); ^{13}C NMR (101 MHz, DMSO-d$_6$, ppm): δ = 136.51 (imC(2)), 123.6 & 122.3 (imC(4,5)), 119.5 (q, J_{CF} = 322 Hz, 2C, NTf$_2$), 48.5 (methyl-C(1)), 35.7 (butyl-C(1)), 31.4 (butyl-C(2)), 18.8 (butyl-C(3)), 13.2 (butyl-(C(4)); MS (ES): *m/z* calcd for M+ 139.1; found: 139.1 (100%). MS (ES): *m/z* calcd for M- 279.8; found: 279.8 (100%).

[C$_8$C$_1$im][NTf$_2$]. Lithium bis(trifluoromethylsulfonyl)imide (10 g, 34.8 mmol, 1.1 eq.) was added to a stirring solution of [C$_8$C$_1$im]Br (8.7 g, 31.6 mmol, 1 eq.) in DCM (50 mL). After the purification steps, the IL was dried overnight on the Schlenk line to yield a viscous colourless liquid.

^{1}H NMR (400 MHz, DMSO-d$_6$, ppm): δ = 9.09 (s, 1H, imC(2)**H**), 7.61 (t, J = 1.8 Hz, 1H, imC(4)**H**), 7.53 (t, J = 1.8 Hz, 1H, imC(5)**H**), 4.10 (t, J = 7.2 Hz, 2H, octyl-C(1)**H$_2$**), 3.79 (s, 1H, methyl-C(1)**H$_3$**), 1.73 (m, 2H, octyl-C(2)**H$_2$**), 1.25 (m, 12H, octyl-C(3-7)**H$_2$**), 0.81 (t, J = 7.4 Hz, 3H, octyl-C(8)**H$_3$**); ^{13}C NMR (101 MHz, DMSO-d$_6$, ppm): δ = 136.4 (imC(2)), 123.3 & 122.0 (imC(4,5)), 119.5 (q, J_{CF} = 322 Hz, 2C, NTf$_2$), 48.5 (methyl-C(1)), 35.3 (octyl-C(1)), 31.4 (octyl-C(2)), 29.2 (octyl-C(3)), 28.5 (octyl-(C(4)), 28.3 (octyl-C(5)), 25.2 (octyl-C(6)), 19.8 (octyl-C(7)), 13.6 (octyl-C(8)); MS (ES): *m/z* calcd for M+ 195.2; found: 195.2 (100%). MS (ES): *m/z* calcd for M- 279.8; found: 279.8 (100%).

[C$_8$C$_1$im][FSI]. Lithium bis(fluorosulfonyl)imide (3.7 g, 19.6 mmol, 1.1 eq.) was added to a stirring solution of [C8C1im]Br (4.9 g, 17.8 mmol, 1 eq.) in DCM (50 mL). After the purification steps the ionic liquid was dried overnight on the Schlenk line to yield a viscous colourless liquid (6.4 g, 17.1 mmol, 96% yield).

^{1}H NMR (400 MHz, DMSO-d$_6$, ppm): δ = 9.09 (s, 1H, imC(2)**H**), 7.76 (t, J = 1.79 Hz, 1H, imC(4)**H**), 7.69 (t, J = 1.77 Hz, 1H, imC(5)**H**), 4.14 (t, J = 7.2 Hz, 2H, octyl-C(1)**H$_2$**), 3.84 (s, 1H, methyl-C(1)**H$_3$**), 1.77 (m, 2H, octyl-C(2)**H$_2$**), 1.25 (m, 12H, octyl-C(3-7)**H$_2$**), 0.86 (t, J = 7.4 Hz, 3H, octyl-C(8)**H$_3$**); ^{13}C NMR (101 MHz, DMSO-d$_6$, ppm): δ = 136.5 (imC(2)), 123.6 & 122.3 (imC(4,5)), 48.8 (methyl-C(1)), 35.7 (octyl-C(1)), 31.1 (octyl-C(2)), 29.4 (octyl-C(3)), 28.5 (octyl-(C(4)), 28.3 (octyl-C(5)), 25.5 (octyl-C(6)), 22.0 (octyl-C(7)), 13.9 (octyl-C(8)); MS (ES): *m/z* calcd for M+ 195.19; found: 195.18 (100%). MS (ES): *m/z* calcd for M- 179.92; found: 179.48 (100%).

[C$_8$C$_1$C$_1$im][NTf$_2$]. Lithium bis(trifluoromethylsulfonyl)imide (10 g, 34.8 mmol, 1.1 eq.) was added to a stirring solution of [C$_8$C$_1$C$_1$im]Br (9.1 g, 31.6 mmol, 1 eq.) in DCM (50 mL). After the purification steps, the IL was dried overnight on the Schlenk line to yield a viscous colourless liquid (14.5 g, 30.6 mmol, 97% yield).

^{1}H NMR (400 MHz, DMSO-d$_6$, ppm): δ = 7.64 (d, J = 2.1 Hz, 1H, imC(4)**H**), 7.60 (t, J = 2.1 Hz, 1H, imC(5)**H**), 4.09 (t, J = 7.3 Hz, 2H, octyl-C(1)**H$_2$**), 3.74 (s, 1H, methyl-C(1)**H$_3$**), 2.57 (s, 1H, im-C**H$_3$**), 1.69 (m, 2H, octyl-C(2)**H$_2$**), 1.26 (m, 12H, octyl-C(3-7)H2), 0.86 (t, J = 7.4 Hz, 3H, octyl-C(8)**H$_3$**); ^{13}C NMR (101 MHz, DMSO-d$_6$, ppm): δ = 144.2 (imC(2)), 122.3 & 120.8 (imC(4,5)), 119.4 (q, J_{CF} = 322 Hz, 2C,

NTf_2), 47.5 (methyl-C(1)), 34.6 (octyl-C(1)), 31.1 (octyl-C(2)), 29.2 (octyl-C(3)), 28.5 (octyl-(C(4)), 28.4 (octyl-C(5)), 25.6 (octyl-C(6)), 22.0 (octyl-C(7)), 13.9 (octyl-C(8)), 9.1 (imC(2')); MS (ES): *m/z* calcd for M+ 209.2; found: 209.2 (100%). MS (ES): *m/z* calcd for M- 279.8; found: 279.8 (100%).

Synthesis of [C_2C_1im][$NMes_2$]

The synthesis of [C_2C_1im][$NMes_2$] requires the synthesis of the precursor acid H[$NMes_2$] (optimised by Philippi *et al.*).[3] Then [C_2C_1im][$NMes_2$] is synthesised *via* the high-pressure route with dimethyl carbonate according to Scheme 2. The high-pressure reaction is discussed in detail by Koutsoukos *et al.*[1]

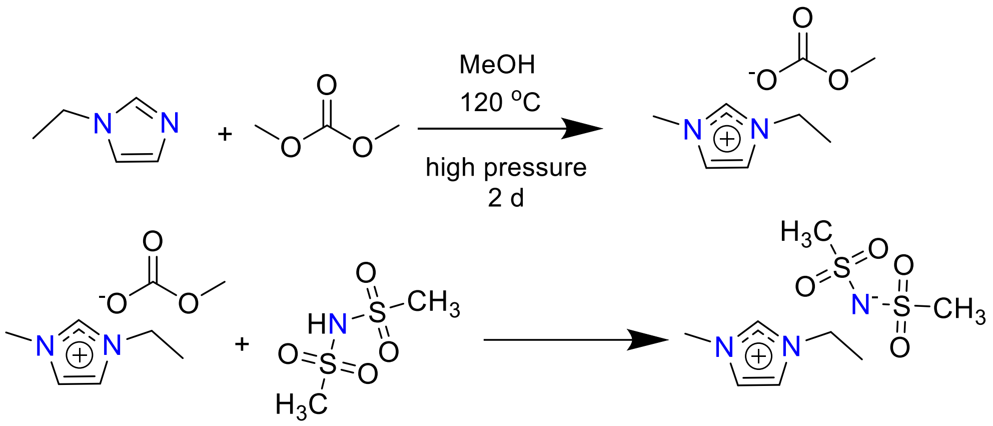

Scheme S2. Synthetic route for the synthesis of [C_2C_1im][$NMes_2$].

H[$NMes_2$]. NH_4Cl (26.3 g, 492 mmol, 1 eq.) was dissolved in 130 mL de-ionised water in a round bottom flask connected with two dropping funnels. One dropping funnels contained methanesulfonyl chloride (114 g, 995 mmol, 2.02 eq.) and the other a 200 mL aqueous solution of sodium hydroxide (79 g NaOH, 1.98 mol, 4.02 eq.). Both liquids were added simultaneously dropwise, over 3 h, while cooling in an ice bath. After addition was completed additional NaOH solution was added until the pH > 7 as tested by pH strips. The solution was transferred to a Kutcher-Steudel apparatus and was extracted with DCM for 24 h. The DCM solution was disposed, the collection flask was replaced with fresh DCM and 60 mL concentrated HCl were added to the aqueous phase. The aqueous phase was extracted for another 24 h. The organic phase was dried over $MgSO_4$ and then DCM was removed *in vacuo*. The solid product was recrystallised twice from acetone and dried on the Schlenk line overnight to yield a white solid (13.6 g, 78.5 mmol, 15.9% yield)

^1H NMR (400 MHz, acetone-d_6, ppm): δ = 6.13 (s, 1H, N-**H**), 3.31 (s, 6H, C**H₃**); ^{13}C NMR (101 MHz, DMSO-d_6, ppm): δ = 43.53 (s, CH_3); HRMS (ES): *m/z* calcd for M- 171.9738; found: 171.9719 (100%).

[C_2C_1im][$NMes_2$]. 1-ethylimidazole was pre-purified by stirring overnight over KOH pellets, followed by vacuum distillation. Purified 1-ethylimidazole (1.03 g, 10.71 mmol, 1 eq.), dimethyl carbonate (2.17 g, 24.11 mmol, 2.25 eq.) and 15 mL of methanol were mixed in a 25 mL thick-walled high-pressure tube and were heated at 120 °C for 2 days. The reaction was monitored by ^1H NMR showing full consumption of the starting 1-ethylimidazole. The solution was transferred to a 50 mL round bottom flask and to that

H[NMes$_2$] (1.86 g, 10.71 mmol, 1 eq.) dissolved in 10 mL methanol was added dropwise. The solution was left stirring at room temperature until no more CO_2 release was observed. The methanolic solution was treated with activated charcoal for 24 h, followed by gravity filtration with filter paper, filtration with 0.25 um PTFE filter and C-18 reversed phase HPLC column. The solvent was removed *in vacuo* and the IL was dried overnight on the Schlenk line to yield a colourless viscous liquid (2.78 g, 9.81 mmol, 91.6% yield).

^{1}H NMR (400 MHz, DMSO-d$_6$, ppm): δ = 9.11 (s, 1H, imC(2)**H**), 7.78 (t, J = 1.75 Hz, 1H, imC(4)**H**)), 7.69 (t, J = 1.73 Hz, 1H, imC(5)**H**), 4.21 (q, J = 7.30 Hz, 2H, ethyl – C(1)**H$_2$**), 3.84 (s, 3H, methyl – C**H$_3$**), 2.72 (s, 6H, NMes$_2$), 1.41 (t, J=7.28 Hz, 3H, ethyl – C**H$_3$**); ^{13}C NMR (101 MHz, DMSO-d$_6$, ppm): δ = 136.25 (imC(2)), 123.59 (imC(4)), 121.99 (imC(5)), 44.12 (ethyl – C(1)), 42.15 (NMes$_2$), 35.71 (methyl – C(1)), 15.13 (ethyl – C(2)); MS (ES): *m/z* calcd for M+ 111.09; found: 111.09 (100%). MS (ES): *m/z* calcd for M- 171.97; found: 171.97 (100%).

Synthesis of deuterated ILs

For the deuteration of the imidazolium hydrogens of [C_2C_1im]Br and [C_8C_1im]Br, the procedure of Giernoth and Bankmann was followed with slight modifications.[4]

[d$_3$-C_2C_1im]Br. Pre-dried [C_2C_1im]Br (10 g, 52.3 mmol, 1 eq.) was mixed with D_2O (3 g, 150 mmol, 2.9 eq.) and Na_2CO_3 (1.1 g, 10.5 mmol, 0.2 eq.) and was left stirring for 24 h at 60 °C. The progress of the deuteration was monitored by ^{1}H NMR and then additional 0.5 mL of D_2O were added. Upon completion of the reaction, the D_2O was removed *in vacuo*, the solid was mixed with DCM and filtered with filter paper and then 0.25 um PTFE filter to remove the Na_2CO_3. DCM was removed *in vacuo* and the resulting [d$_3$-C_2C_1im]Br was dried overnight on the Schlenk line. The product showed 97% deuteration yield on the imidazolium hydrogens, based on the integration of the ^{1}H NMR signals corresponding to the residual imidazolium ring hydrogens.

^{1}H NMR (400 MHz, DMSO-d$_6$, ppm): δ = 9.23 (s, 0.03 H, imC(2)**H,** residual non-deuterated)), 7.82 (s, 0.02H, imC(4)**H**, residual non-deuterated)), 7.69 (s, 0.02H, imC(5)**H**, residual non-deuterated), 4.20 (q, J = 7.32 Hz, 2H, ethyl – C(1)**H$_2$**), 3.85 (s, 3H, methyl – C**H$_3$**), 1.41 (t, J=7.34 Hz, 3H, ethyl – C**H$_3$**); ^{13}C NMR (101 MHz, $CDCl_3$, ppm): δ = 136.70 (imC(2)), 123.32 (imC(4)), 121.59 (imC(5)), 45.15 (ethyl - C(1)), 36.62 (methyl – C(1)), 15.62 (ethyl – C(2)); MS (ES): *m/z* calcd for M+ 114.11; found: 114.09 (100%). MS (ES): *m/z* calcd for M- 80.9; found: 80.9 (100%).

[d$_3$-C_2C_1im][NTf2]. Lithium bis(trifluoromethylsulfonyl)imide (8.1 g, 28.3 mmol, 1.1 eq.) was added to a stirring solution of [d$_3$-C_2C_1im]Br (5 g, 25.8 mmol, 1 eq.) in DCM (30 mL). After the purification steps, the IL was dried overnight on the Schlenk line to yield a viscous colourless liquid (9.6 g, 24.3 mmol, 94% yield).

^{1}H NMR (400 MHz, DMSO-d$_6$, ppm): δ = 9.10 (s, 0.03 H, imC(2)**H,** residual non-deuterated)), 7.76 (s, 0.02H, imC(4)**H**, residual non-deuterated)), 7.68 (s, 0.02H, imC(5)**H**, residual non-deuterated), 4.18 (q, J = 7.34 Hz, 2H, ethyl – C(1)**H$_2$**), 3.84 (s, 3H, methyl – C**H$_3$**), 1.41 (t, J=7.30 Hz, 3H, ethyl – C**H$_3$**); ^{13}C NMR (101 MHz, DMSO-d$_6$, ppm): δ = 136.91 (t, J = 34 Hz, imC(2)), 123.22 (t, J = 31 Hz, imC(4)),

121.66 (t, J = 31 Hz, imC(5)), 119.49 (q, J_{CF} = 322 Hz, 2C, NTf_2), 44.18 (ethyl - C(1)), 35.67 (methyl – C(1)), 15.02 (ethyl – C(2)); MS (ES): *m/z* calcd for M+ 114.11; found: 114.09 (100%). MS (ES): *m/z* calcd for M- 279.8; found: 279.8 (100%).

[d_3-C_8C_1im]Br. Pre-dried [C_8C_1im]Br (15 g, 76.8 mmol, 1 eq.) was mixed with D_2O (4.5 g, 222.7 mmol, 2.9 eq.) and Na_2CO_3 (1.6 g, 15.4 mmol, 0.2 eq.) and was left stirring for 24 h at 60 °C. The progress of the deuteration was monitored by ^1H NMR and then additional 1 mL of D_2O were added. Upon completion of the reaction, the D_2O was removed *in vacuo*, the solid was mixed with DCM and filtered with filter paper and then 0.25 um PTFE filter to remove the Na_2CO_3. DCM was removed *in vacuo* and the resulting [d_3-C_2C_1im]Br was dried overnight on the Schlenk line. The product showed 96% deuteration yield on the C^2 imidazolium hydrogens and 99% yield on the $C^{3/4}$ ring hydrogens, based on the integration of the ^1H NMR signals corresponding to the residual imidazolium ring hydrogens.

^1H NMR (400 MHz, $CDCl_3$, ppm): δ = 10.35 (s, 0.04H, imC(2)**H**, residual non-deuterated), 7.58 (s, 0.01H, imC(4)**H**, residual non-deuterated), 7.41 (s, 1H, imC(5)**H**, residual non-deuterated), 4.28 (t, J = 7.45 Hz, 2H, octyl-C(1)**H_2**), 4.09 (s, 1H, methyl-C(1)**H_3**), 1.87 (m, 2H, octyl-C(2)**H_2**), 1.25 (m, 12H, octyl-C(3-7)**H_2**), 0.82 (t, J = 6.53 Hz, 3H, octyl-C(8)**H_3**); ^{13}C NMR (101 MHz, $CDCl_3$, ppm): δ = 137.4 (imC(2)), 123.4 & 121.6 (imC(4,5)), 50.2 (methyl-C(1)), 36.7 (octyl-C(1)), 31.7 (octyl-C(2)), 30.3 (octyl-C(3)), 29.0 (octyl-(C(4)), 28.9 (octyl-C(5)), 26.3 (octyl-C(6)), 22.6 (octyl-C(7)), 14.1 (octyl-C(8)); MS (ES): *m/z* calcd for M+ 198.20; found: 198.15 (100%). MS (ES): *m/z* calcd for M- 80.9; found: 80.9 (100%).

[d_3-C_8C_1im][NTf2]. Lithium bis(trifluoromethylsulfonyl)imide (5.7 g, 19.8 mmol, 1.1 eq.) was added to a stirring solution of [d_3-C_8C_1im]Br (5 g, 18 mmol, 1 eq.) in DCM (30 mL). After the purification steps, the IL was dried overnight on the Schlenk line to yield a viscous colourless liquid (7.8 g, 16.4 mmol, 91% yield).

^1H NMR (400 MHz, DMSO-d_6, ppm): δ = 9.08 (s, 0.04H, imC(2)**H**, residual non-deuterated), 7.75 (s, 0.01H, imC(4)**H**, residual non-deuterated), 7.65 (s, 1H, imC(5)**H**, residual non-deuterated), 4.14 (t, J = 7.21 Hz, 2H, octyl-C(1)**H_2**), 3.83 (s, 1H, methyl-C(1)**H_3**), 1.77 (m, 2H, octyl-C(2)**H_2**), 1.24 (m, 12H, octyl-C(3-7)**H_2**), 0.85 (t, J = 6.54 Hz, 3H, octyl-C(8)**H_3**); ^{13}C NMR (101 MHz, DMSO-d_6, ppm): δ = 136.4 (imC(2)), 123.6 & 122.2 (imC(4,5)), 48.8 (methyl-C(1)), 35.8 (octyl-C(1)), 31.2 (octyl-C(2)), 29.4 (octyl-C(3)), 28.5 (octyl-(C(4)), 28.4 (octyl-C(5)), 25.5 (octyl-C(6)), 22.2 (octyl-C(7)), 14.0 (octyl-C(8)); MS (ES): *m/z* calcd for M+ 198.20; found: 198.15 (100%). MS (ES): *m/z* calcd for M- 279.8; found: 279.8 (100%).

Synthesis of spin probe C8-OTEMPO

For the synthesis of the alkyloxy functionalised TEMPO derivative, the synthetic procedure that was followed is shown in Scheme **3**, and it is a modified procedure based on the works of Bossmann *et al.*[5]

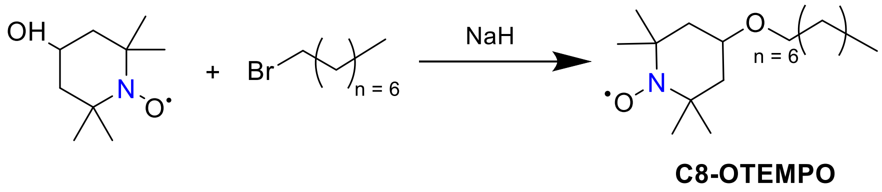

Scheme S3. Synthetic routes for the synthesis of the spin probe **C8-OTEMPO**.

C8-OTEMPO. TEMPOL (1 g, 5.8 mmol, 1 eq.) dissolved in dry THF (10 mL) was added dropwise to a stirring suspension of NaH (60% in mineral oil, 0.17 g, 7.0 mmol {0.28 g as 60% in oil}, 1.2 eq.) in dry THF (10 mL) in a 50 mL round bottom flask, under dry N_2 atmosphere, and was stirred over an ice bath for 1 h. Then 1-bromooctane (6.7 g, 34.8 mmol, 6.0 eq.) was added dropwise to the suspension over a period of 45 min. The system was allowed to reach room temperature overnight and then it was heated to 50 ^{o}C for 1 h. Then the reaction slurry was poured into cold water (250 mL) and extracted with DCM (4 x 100 mL). The combined organics were dried over $MgSO_4$, filtered, and evaporated *in vacuo*. The residue was purified by column chromatography (EtOAc:cyclohexane 20:80) to give an orange oil (0.76 g, 2.7 mmol, 46% yield).

^{1}H NMR (400 MHz, MeCN-d_3 with 1 drop of pentafluorophenyl hydrazine, ppm): δ = 3.59 – 3.33 (m, 3H, octyl-C(1)$\mathbf{H_2}$ & TEMPO-C(3)**H**), 1.89-1.78 & 1.51-1.37 (m, 4H, TEMPO-C(2,2')$\mathbf{H_2}$), 1.28 (m, 12H, TEMPO-4xC$\mathbf{H_3}$), 1.13 (m, 2H, octyl-C(7)$\mathbf{H_2}$), 1.09 (m, 10H, octyl-C(2-6)$\mathbf{H_2}$), 0.88 (t, J = 6.67 Hz, 3H, octyl-C(8)$\mathbf{H_3}$); HRMS (ES): *m/z* calcd for M+ 286.2696; found 286.2714.

Purity was also confirmed by HPLC-MS on a Poroshell HPH-C18 3.0x50mm 2.7um column. Compound was eluted at 7.668 min (98%), ES m/z M+ found 286.30.

Synthesis of spin labelled ILs

For the synthesis of the IL functionalised TEMPO derivatives, the synthetic procedure that was followed is shown in Scheme **4**, and it is a modified procedure based on the works of Bossmann *et al.*,[5] and Ivanov *et al.*[6]

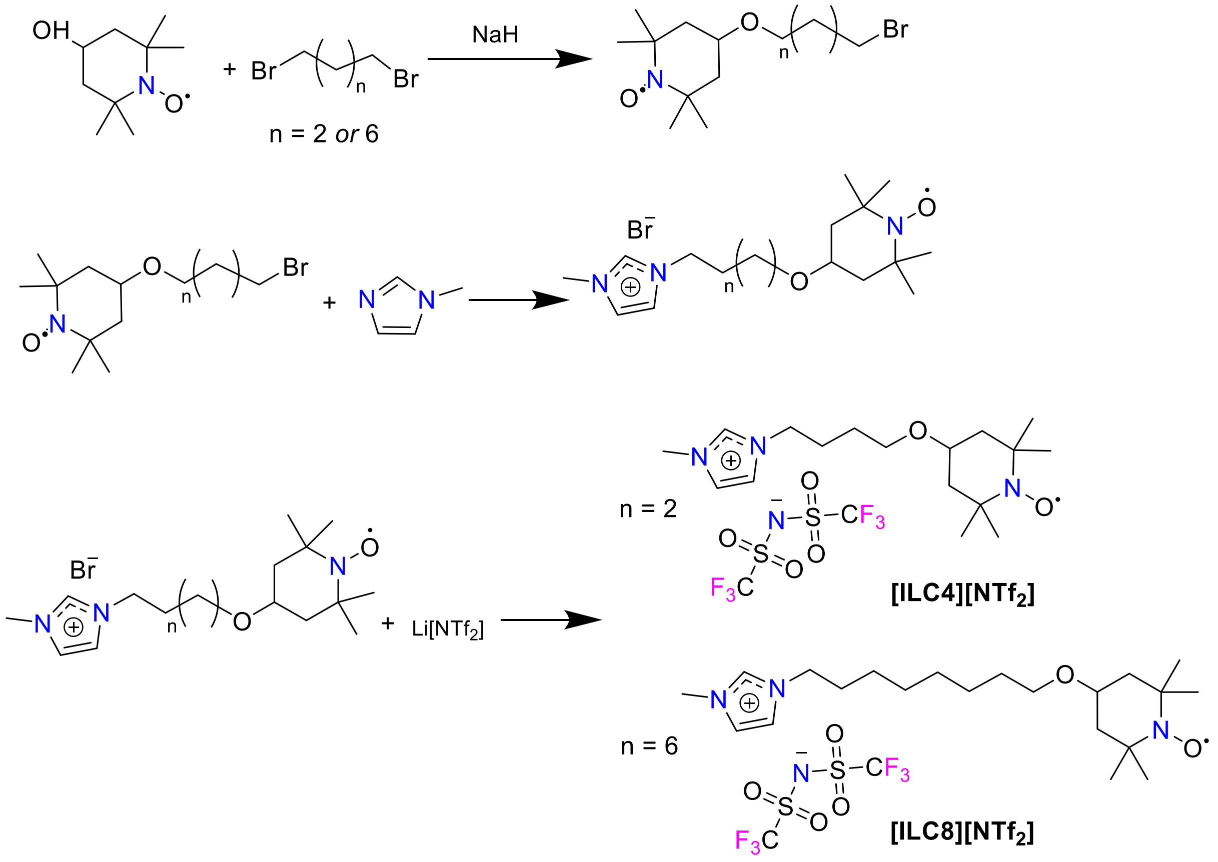

Scheme S4. Synthetic routes for the synthesis of the spin labelled ILs.

BrC4-OTEMPO. TEMPOL (0.77 g, 4.5 mmol, 1 eq.) dissolved in dry THF (10 mL) was added dropwise to a stirring suspension of NaH (60% in mineral oil, 0.13 g, 5.4 mmol {0.22 g as 60% in oil}, 1.2 eq.) in dry THF (10 mL) in a 50 mL round bottom flask, under dry N_2 atmosphere, and was stirred over an ice bath for 1 h. Then 1,4-dibromobutane (5.8 g, 26.9 mmol, 6.0 eq.) was added dropwise to the suspension over a period of 45 min. The system was allowed to reach room temperature overnight and then it was heated to 50 °C for 1 h. Then the reaction slurry was poured into cold water (250 mL) and extracted with DCM (4 x 100 mL). The combined organics were dried over $MgSO_4$, filtered, and evaporated *in vacuo*. The residue was purified by column chromatography (EtOAc:cyclohexane 20:80) to give an orange powder (0.35 g, 1.1 mmol, 2% yield).

^{1}H NMR (400 MHz, $CDCl_3$ with 1 drop of pentafluorophenyl hydrazine, ppm): 3.58 (m, 1H, TEMPO-C(3)**H**), 3.48 (m, 4H, butyl-C(1,4)**H₂**), 1.76 – 1.69 (m, 4H, butyl-C(2,3)**H₂**, 1.76 – 1.43 (m, 4H, TEMPO-C(2,2')**H₂**), 1.29 – 1.14 (m, 12H, TEMPO-4xC**H₃**); HRMS (ES): *m/z* calcd for M+ 308.1220; found 308.1212.

Purity was also confirmed by HPLC-MS on a Poroshell HPH-C18 3.0x50mm 2.7um column. Compound was eluted at 5.685 min (95%), ES *m/z* M+ found 310.10.

[ILC4]Br. BrC4-OTEMPO (0.18 g, 0.6 mmol, 1 eq.) was added to a stirring solution of purified 1-methylimidazole (0.047 g, 0.57 mmol, 0.95 eq.) in acetonitrile (30 mL). The reaction mixture was heated to 40 ^{o}C and the reaction progress was monitored by TLC. Upor completion of the reaction, the solvent was removed *in vacuo* and the salt was purified by column chromatography (elute with dichloromethane:ethyl acetate 8:2 to wash unreacted starting material and then the salt is retrieved by eluting with dichloromethane: ethyl acetate: methanol 6:2:2). The collected organics were dried over $MgSO_4$, and the solvents were removed *in vacuo*. The product was dried overnight on the Schlenk line to yield a light orange solid (0.19 g, 0.50 mmol, 83% yield).

^{1}H NMR (400 MHz, DMSO-d_6 with 1 drop of pentafluorophenyl hydrazine, ppm): δ = 9.14 (s, 1H, imC(2)**H**), 7.78 (s, 1H, imC(4)**H**)), 7.72 (s, 1H, imC(5)**H**), 4.18 (t, J = 7.10 Hz, 2H, butyl – C(4)$\mathbf{H_2}$), 3.85 (s, 3H, methyl – C$\mathbf{H_3}$), 3.50 (m, 1H, TEMPO-C(3)**H**), 3.39 (t, J = 6.20 Hz, 2H, butyl-C(1)$\mathbf{H_2}$), 1.82 (m, 4H, butyl C(2,3)$\mathbf{H_2}$), 1.44 & 1.21 (m, 4H, TEMPO-C(2,2')$\mathbf{H_2}$), 1.25 & 1.17 (s, 12H, TEMPO-4xC$\mathbf{H_3}$); HRMS (ES): *m/z* calcd for M+ 309.2411; found: 309.1131 (100%). MS (ES): *m/z* calcd for M- 80.9; found: 80.92 (100%).

Purity was also confirmed by HPLC-MS on a Poroshell HPH-C18 3.0x50mm 2.7um column. Compound was eluted at 2.811 min (98%), ES *m/z* M+ found 309.20.

[ILC4][NTf2]. Lithium bis(trifluoromethylsulfonyl)imide (0.15 g, 0.54 mmol, 1.1 eq.) was added to a stirring solution of [ILC4]Br (0.19 g, 0.49 mmol, 1 eq.) in DCM (25 mL). After the purification steps, the IL was dried overnight on the Schlenk line to yield a viscous orange liquid (0.26 g, 0.44 mmol, 90% yield).

^{1}H NMR (400 MHz, MeCN-d_3 with 1 drop of pentafluorophenyl hydrazine, ppm): δ = 8.38 (s, 1H, imC(2)**H**), 7.36 (s, 1H, imC(4)**H**)), 7.32 (s, 1H, imC(5)**H**), 4.15 (t, J = 7.22 Hz, 2H, butyl – C(4)$\mathbf{H_2}$), 3.81 (s, 3H, methyl – C$\mathbf{H_3}$), 3.56 (m, 1H, TEMPO-C(3)**H**), 3.44 (t, J = 6.09 Hz, 2H, butyl-C(1)$\mathbf{H_2}$), 1.90-1.82 (m, 4H, butyl C(2,3)$\mathbf{H_2}$), 1.48 & 1.28 (m, 4H, TEMPO-C(2,2')$\mathbf{H_2}$), 1.11 & 1.09 (s, 12H, TEMPO-4xC$\mathbf{H_3}$); MS (ES): *m/z* calcd for M+ 309.24; found: 309.24 (100%); MS (ES): *m/z* calcd for M- 279.92; found: 279.92 (99%).

Purity was also confirmed by HPLC-MS on a Poroshell HPH-C18 3.0x50mm 2.7um column. Compound was eluted at 2.720 min (100%), ES *m/z* M+ found 309.20.

BrC8-OTEMPO. TEMPOL (1 g, 5.8 mmol, 1 eq.) dissolved in dry THF (10 mL) was added dropwise to a stirring suspension of NaH (60% in mineral oil, 0.17 g, 7.0 mmol {0.28 g as 60% in oil}, 1.2 eq.) in dry THF (10 mL) in a 50 mL round bottom flask, under dry N_2 atmosphere, and was stirred over an ice bath for 1 h. Then 1,8-dibromooctane (9.5 g, 34.8 mmol, 6.0 eq.) was added dropwise to the suspension over a period of 45 min. The system was allowed to reach room temperature overnight and then it was heated to 50 ^{o}C for 1 h. Then the reaction slurry was poured into cold water (250 mL) and extracted with DCM (4 x 100 mL). The combined organics were dried over $MgSO_4$, filtered, and evaporated *in*

vacuo. The residue was purified by column chromatography (EtOAc:cyclohexane 20:80) to give an orange powder (0.86 g, 2.4 mmol, 41% yield).

^{1}H NMR (400 MHz, $CDCl_3$ with 1 drop of pentafluorophenyl hydrazine, ppm): δ = 3.58 (m, 1H, TEMPO-C(3)**H**), 3.43 (m, 4H, octyl-C(1,8)$\mathbf{H_2}$), 2.00 – 1.82 (m, 4H, TEMPO-C(2,2')$\mathbf{H_2}$), 1.61 – 1.11 (m, 24H, octyl-C(2-7)$\mathbf{H_2}$ and TEMPO-4xC$\mathbf{H_3}$); HRMS (ES): *m/z* calcd for M+ 366.1747; found 366.1851.

Purity was also confirmed by HPLC-MS on a Poroshell HPH-C18 3.0x50mm 2.7um column. Compound was eluted at 5.292 min (98%), ES *m/z* M+ found 365.20.

[ILC8]Br. BrC8-OTEMPO (0.18 g, 0.5 mmol, 1 eq.) was added to a stirring solution of purified 1-methylimidazole (0.046 g, 0.55 mmol, 1.1 eq.) in acetonitrile (30 mL). The reaction mixture was heated to 40 ^oC and the reaction progress was monitored by TLC. Upor completion of the reaction, the solvent was removed *in vacuo* and the salt was purified by column chromatography (elute with dichloromethane:ethyl acetate 8:2 to wash unreacted starting material and then the salt is retrieved by eluting with dichloromethane: ethyl acetate: methanol 6:2:2). The collected organics were dried over $MgSO_4$, and the solvents were removed *in vacuo*. The product was dried overnight on the Schlenk line to yield a light orange solid (0.16 g, 0.36 mmol, 72% yield).

^{1}H NMR (400 MHz, $CDCl_3$ with 1 drop of pentafluorophenyl hydrazine, ppm): δ = 10.44 (s, 1H, imC(2)**H**), 7.46 (s, 1H, imC(4)**H**)), 7.36 (s, 1H, imC(5)**H**), 4.30 (t, J = 7.45 Hz, 2H, ethyl – C(1)$\mathbf{H_2}$), 4.10 (s, 3H, methyl – C$\mathbf{H_3}$), 3.55 (m, 1H, TEMPO-C(3)**H**), 3.39 (t, J = 6.57 Hz, 2H, octyl-C(1)$\mathbf{H_2}$), 1.91 & 1.51 (m, 8H, TEMPO-C(2,2')$\mathbf{H_2}$ & octyl-C(2,3)$\mathbf{H_2}$), 1.29 (m, 10H, octyl-C(4-8)$\mathbf{H_2}$), 1.25 & 1.17 (s, 12H, TEMPO-4xC$\mathbf{H_3}$); MS (ES): *m/z* calcd for M+ 365.30; found: 365.30 (100%). MS (ES): *m/z* calcd for M- 80.9; found: 80.92 (100%).

Purity was also confirmed by HPLC-MS on a Poroshell HPH-C18 3.0x50mm 2.7um column. Compound was eluted at 3.684 min (100%), ES *m/z* M+ found 365.20.

[ILC8][NTf2]. Lithium bis(trifluoromethylsulfonyl)imide (0.11 g, 0.4 mmol, 1.1 eq.) was added to a stirring solution of [ILC8]Br (0.16 g, 0.36 mmol, 1 eq.) in DCM (25 mL). After the purification steps, the IL was dried overnight on the Schlenk line to yield a viscous orange liquid (0.23 g, 0.35 mmol, 96% yield).

^{1}H NMR (400 MHz, MeCN-d_k with 1 drop of pentafluorophenyl hydrazine, ppm): δ = 8.38 (s, 1H, imC(2)**H**), 7.36 (s, 1H, imC(4)**H**)), 7.32 (s, 1H, imC(5)**H**), 4.10 (t, J = 7.29 Hz, 2H, ethyl – C(1)$\mathbf{H_2}$), 3.8 (s, 3H, methyl – C$\mathbf{H_3}$), 3.5 (m, 1H, TEMPO-C(3)**H**), 3.38 (t, J = 6.52 Hz, 2H, octyl-C(1)$\mathbf{H_2}$), 1.94 (m, 4H, octyl-C(2,3)$\mathbf{H_2}$),1.83 & 1.47 (m, 4H, TEMPO-C(2,2')$\mathbf{H_2}$), 1.30 (m, 10H, octyl-C(4-8)$\mathbf{H_2}$), 1.11 & 1.09 (s, 12H, TEMPO-4xC$\mathbf{H_3}$); MS (ES): *m/z* calcd for M+ 365.30; found: 365.30 (100%). MS (ES): *m/z* calcd for M- 279.92; found: 279.92 (100%).

Purity was also confirmed by HPLC-MS on a Poroshell HPH-C18 3.0x50mm 2.7um column. Compound was eluted at 4.048 min (100%), ES *m/z* M+ found 365.40.

S.2 Experimental methods

EPR sample preparation

All molecular solvents were degassed via freeze-pump-thaw and transferred to a glovebox under inert N_2 atmosphere. Then a stock solution of 4 mM of the desired spin probe or spin labelled IL was prepared, which was further diluted to a final concentration of 0.2 mM. The samples were loaded in 1.6 mm O.D. round end borosilicate glass capillaries (Kimble). The capillaries were sealed with a Hirschmann™ haematocrit wax seal, followed by further sealing with a two-part epoxy resin (Araldite).

All ILs were dried and degassed in high vacuum. For the preparation of the IL samples, an initial 12 mM stock solution of the spin probe or spin labelled IL in DCM was prepared, which was then added to the appropriate amount of IL to make a 0.2 mM final concentration. DCM was removed *in vacuo* and the IL solution was transferred to a glovebox under inert N_2 atmosphere. The solutions were loaded in 1.6 mm O.D. round end borosilicate glass capillaries (Kimble). The capillaries were sealed with a Hirschmann™ haematocrit wax seal, followed by further sealing with a two-part epoxy resin (Araldite).

All samples were stored in the fridge (4 ˚C) and showed no indications of the radical reacting (*e.g.* adduct signals or reduced EPR intensity).

EPR spectroscopy

Continuous-wave X-band EPR measurements were performed at room temperature with a Bruker EMX spectrometer equipped with a high-sensitivity ER 4119HS cylindrical resonator (Bruker), using a microwave power of 0.02 mW, a field modulation of 1 G at 100 kHz, a conversion time of 1.5 ms, a time constant of 10.24 ms and a sweep time of 12.60 s.

All pulse EPR measurements were performed on a Bruker Elexsys E580 spectrometer equipped with an over-coupled EN 5107-D2 ENDOR resonator (Bruker) for Q-band, or an EN 4118X-MD4 (Bruker) for X-band measurements, a Bruker SpinJet Arbitrary Waveform Generator (AWG), a SpecJet-III digitiser (Bruker), a 300 W traveling-wave tube amplifier (Applied Systems Engineering Inc.), and a closed-circuit Helium cryostat (Cryogenic Ltd), equipped with a Lakeshore 350 temperature controller (Lake Shore Cryotronics, Inc.). All measurements were performed at 50 K and employed rectangular AWG microwave pulses. For all measurements, the shot-repetition time employed varied from 3-4 ms depending on the sample, and all spectra were collected with 1 shot-per-point. Radiofrequency (RF) pulses were generated using a DICE-II digital synthesiser (Bruker) and amplified using a 150 W, 0.1 – 250 MHz continuous-wave RF power amplifier (Barthel HF-Technik GmbH).

Echo-Detected Field Sweep (EDFS) measurements were performed using a 2-pulse Hahn echo sequence, $\pi/2$-τ-π-τ-*echo*, using the parameters detailed in S.3. Phase-memory time (T_m) relaxation measurements were performed detecting on the maximum of the nitroxide signal, using the same 2-pulse Hahn echo sequence, where τ was increased in increments of 2 ns.

Electron Spin Echo Envelope Modulation (ESEEM) experiments were performed using the 3-pulse sequence, *π/2-τ-π/2-T-π/2-τ-echo*, detecting on the maximum of the EDFS spectrum, with the parameters set to π/2 = 16 ns, τ = 138 – 148 ns, T = 80 ns, dx = 10 ns, and dy = 6 ns. A 4-step phase cycle was employed.

Electron-Nuclear Double Resonance (ENDOR) experiments were performed using the Mims pulse sequence, $\pi/2\text{-}\tau\text{-}\pi/2\text{-}t_{RF}\text{-}T\text{-}\pi/2\text{-}\tau\text{-}echo$, where the RF π pulse was tuned by a Mims-detected RF nutation experiment on the proton line, where the RF π pulse length was incremented in 100 ns steps. The RF π pulse length was then scaled for optimal excitation of ^{2}H and ^{19}F nuclei based on their gyromagnetic ratios. ENDOR experiments were performed using stochastic excitation of the RF pulse. No phase cycling was applied to the detection sequence. The parameters for each set up are detailed in S.3.

Further Electron-Nuclear Double Resonance (ENDOR) experiments were performed using the Davies pulse sequence $\pi_{inv}\text{-}t_{RF}\text{-}T\text{-}\pi/2\text{-}\tau\text{-}\pi\text{-}echo$, where the π_{inv} pulse was selective (210 to 250 ns) and π/2 = 100 ns, π = 200 ns, τ = 500 ns, with a 1 μs delay before and after the RF π pulse. The RF π pulse length was set to 16 μs and used stochastic excitation, with a sweep width of 20 MHz and a 20 KHz step size. No phase cycling was applied to the detection sequence.

Hyperfine Sublevel Correlation Spectroscopy (HYSCORE) experiments were performed employing either a 4-pulse sequence, $\pi/2\text{-}\tau\text{-}\pi/2\text{-}t_1\text{-}\pi\text{-}\ t_2\text{-}\pi/2\text{-}\tau\text{-}echo$ with a 4-step phase cycle, or a 6-pulse sequence, $\pi/2_y\text{-}\tau\text{-}\pi_y\text{-}\tau\text{-}\pi/2_x\text{-}t_1\text{-}\pi_x\text{-}t_2\text{-}\pi/2_x\text{-}\tau\text{-}\pi_x\text{-}\tau\text{-}echo$ with an 8-step phase cycle. The parameters for each set up are detailed in S.3.

NMR spectroscopy

NMR spectra were recorded on an AVANCE II 400 NMR spectrometer (Bruker, Billerica, USA). The residual signal of the deuterated solvent was used as a reference. Chemicals shifts are given in ppm. Details on the PRE NMR experimental set-up are given in S.6.

S.3 Pulse EPR data

Echo-Detected Field Sweep (EDFS) at X/Q-band (50 K)

The biggest variation seen between anions in the X-band EDFS spectra of **TEMPOL** dissolved in $[C_2C_1im]^+$ is for the $[FSI]^-$ containing sample. A detailed analysis of this spectrum is beyond the scope of this current work.

3-pulse Electron-Spin Echo Envelope Modulation (ESEEM) at X-band (50 K)

While intense $^{10}B/^{11}B$ signals were observed in the 3p-ESEEM spectra of the $[BF_4]^-$ anion, $^{10}B/^{11}B$ pulsed ENDOR is not routinely applied and suffers from a relatively low γ, thus requiring long, weakly attenuated radiofrequency (RF) pulses to achieve the optimum pulse turning angle, which then often experience amplification distortions. Further, complications in the analysis of $^{10}B/^{11}B$ ENDOR measurements can arise due to the higher nuclear spin states for both ^{10}B (I = 3), and ^{11}B (I = 3/2), where the quadrupolar interaction is non-negligible in both cases.

A

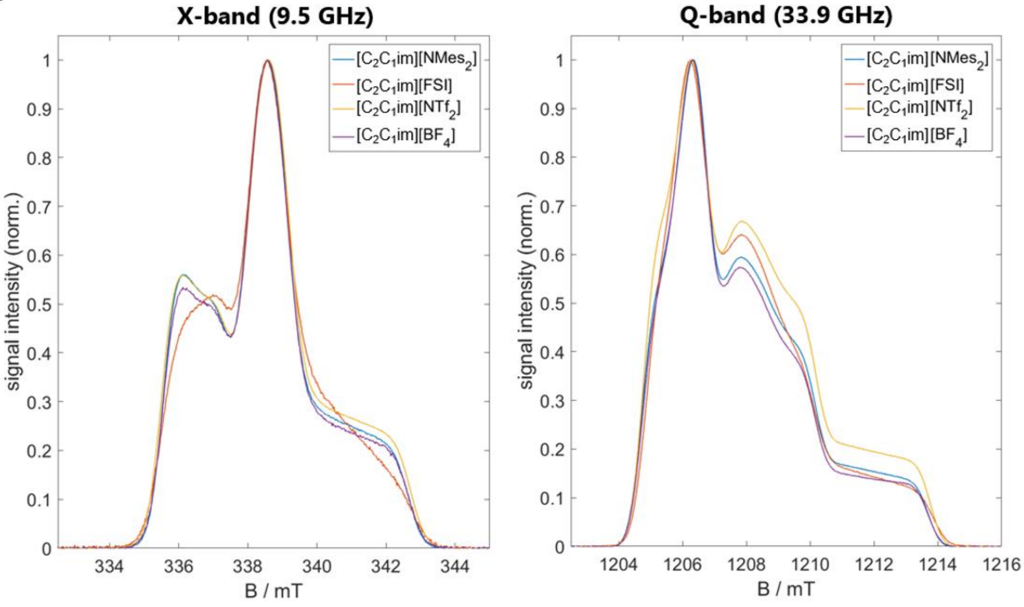

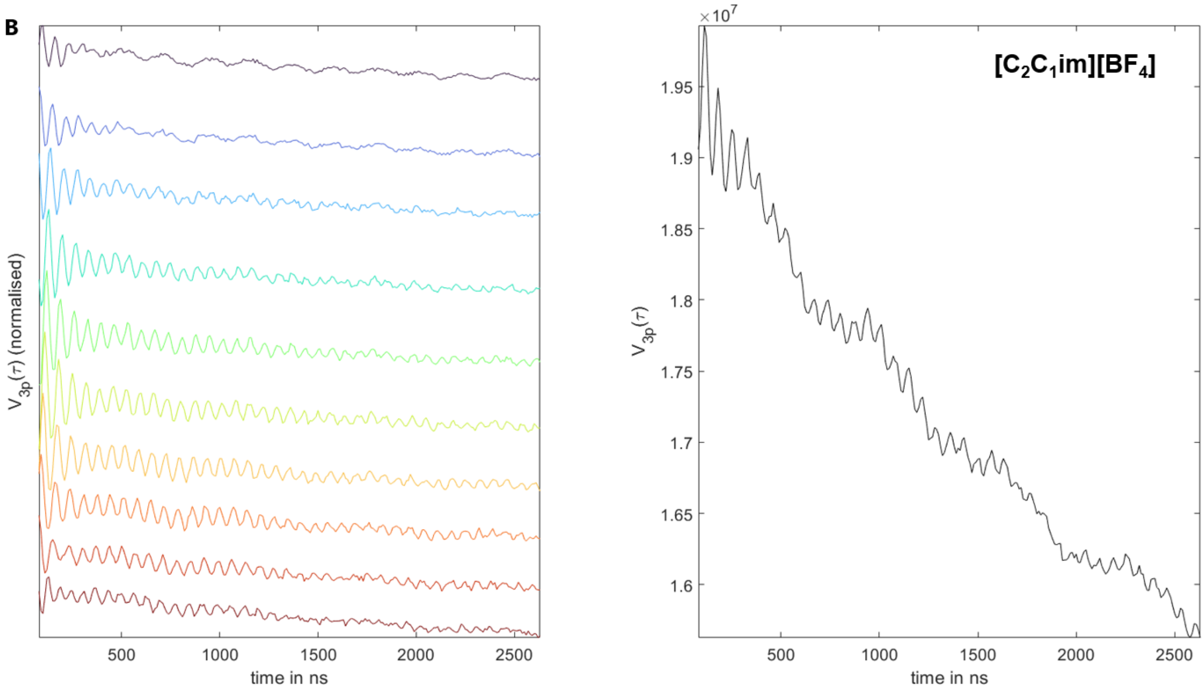
B
V$_{3p}$(τ) (normalised)
time in ns
500
1000
1500
2000
2500
×10^7
[C$_2$C$_1$im][BF$_4$]
V$_{3p}$(τ)
1.95
1.9
1.85
1.8
1.75
1.7
1.65
1.6

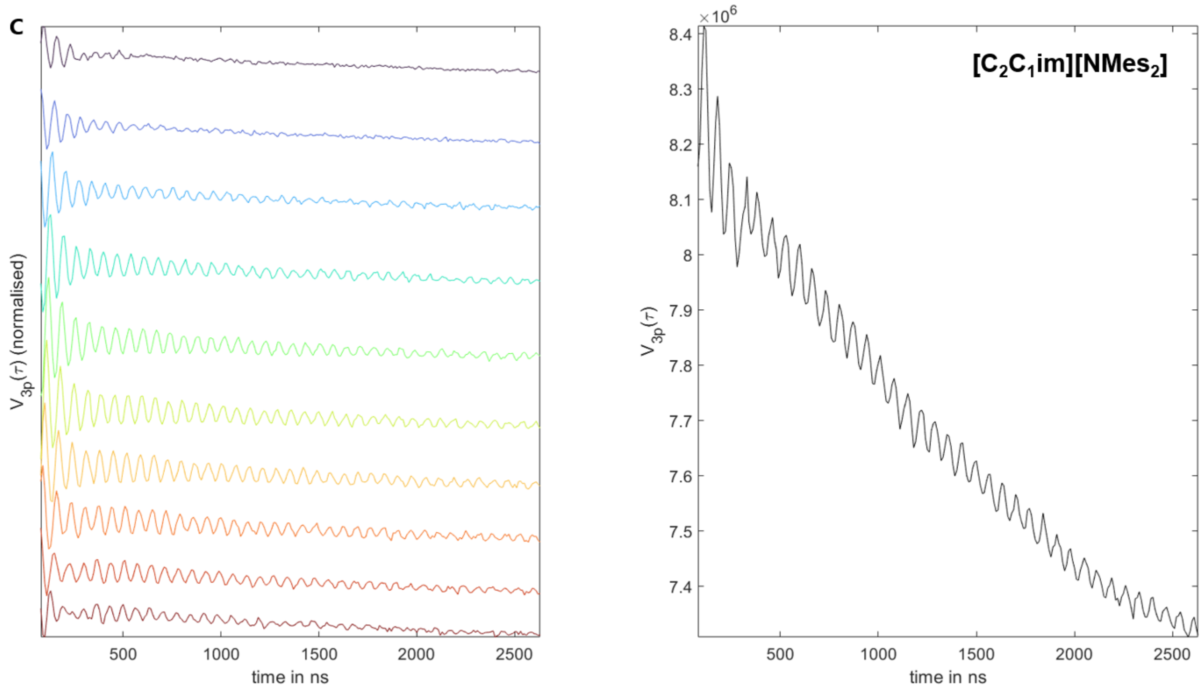
C
V$_{3p}$(τ) (normalised)
time in ns
500
1000
1500
2000
2500
×10^6
[C$_2$C$_1$im][NMes$_2$]
V$_{3p}$(τ)
8.4
8.3
8.2
8.1
8
7.9
7.8
7.7
7.6
7.5
7.4

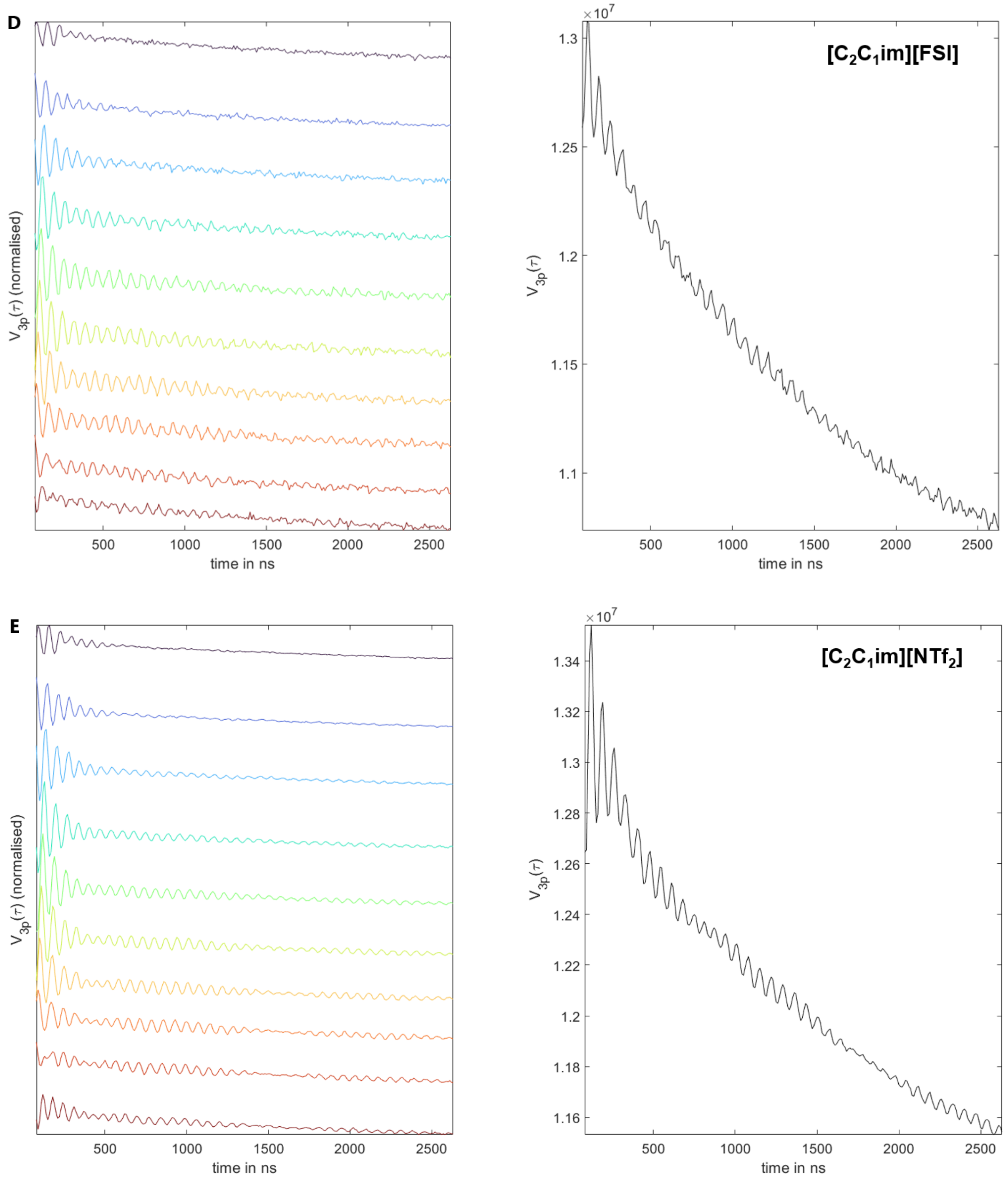

Figure S1. A) EDFS spectra of **TEMPOL** (2mM) in IL solutions measured at 50 K with π/2 = 16 ns, π = 32 ns, and τ = 140 ns (X-band), 200 ns (Q-band). **B-E)** Raw τ-dependent 3P-ESEEM time traces (coloured) as described in the main text and the S.I., and summed time traces (black).

Echo-Detected Field Sweep (EDFS) at X/Q-band (50 K)

Synthesised spin probe

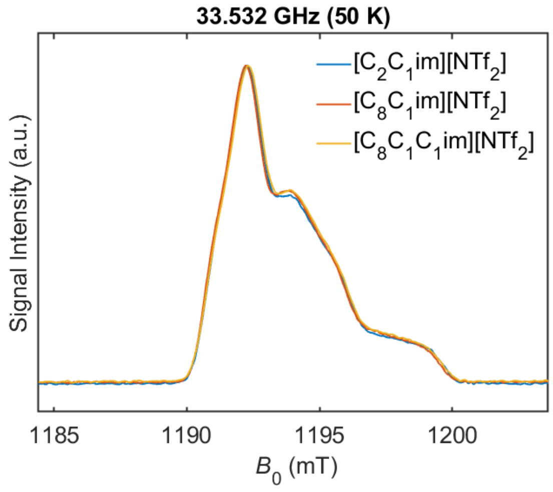

Figure S2. EDFS spectra of **C8-OTEMPO** (200 μM) in IL solutions ($[C_2C_1im][NTf_2]$, $[C_8C_1im][NTf_2]$ and $[C_8C_1C_1im][NTf_2]$) measured at 50 K with π/2 = 12 ns, π = 24 ns, and τ = 320 ns.

Spin-labelled ILs

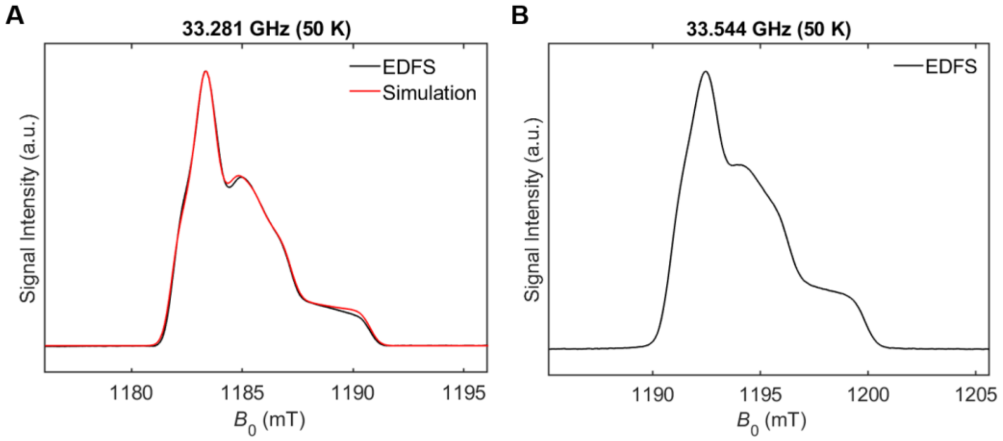

Figure S3. Q-band (50 K) EDFS spectra (black) of **A) [ILC4][NTf₂]** dissolved in CD_2Cl_2/d_8-toluene (black) and corresponding simulation (red) using the *EasySpin* function 'pepper.m' (*g* = [2.0070, 2.0103, 2.0028], *A* = [21.3179 22.1543 99.9272]; linewidth = 0.8014 mT), **B) [ILC8][NTf₂]** dissolved in CD_2Cl_2/d_8-toluene (black).

Phase memory time (T_m) at X/Q-band 50K

Spin probes

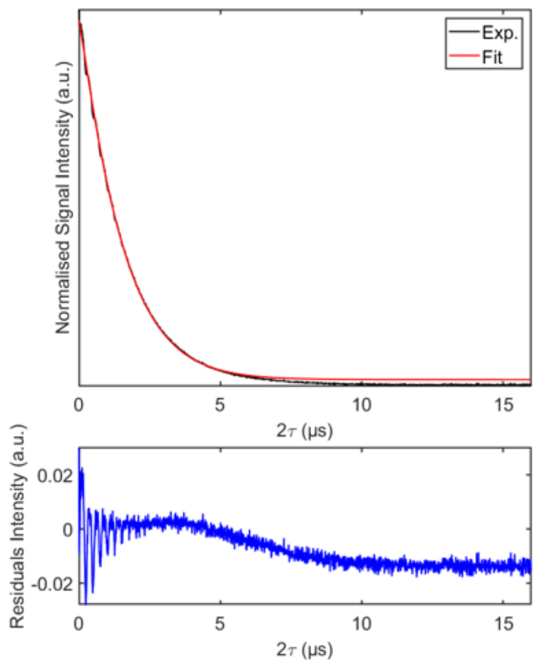

Figure S4. Phase memory time (T_m) measurement of **TEMPOL** (2 mM) at Q-band in [d_3-C_2C_1im][NTf_2] (black) and corresponding stretched exponential fit (*x* = stretched exponent) (red), and residuals (blue). Fitted T_m = 1.57 μs, *x* = 1.11.

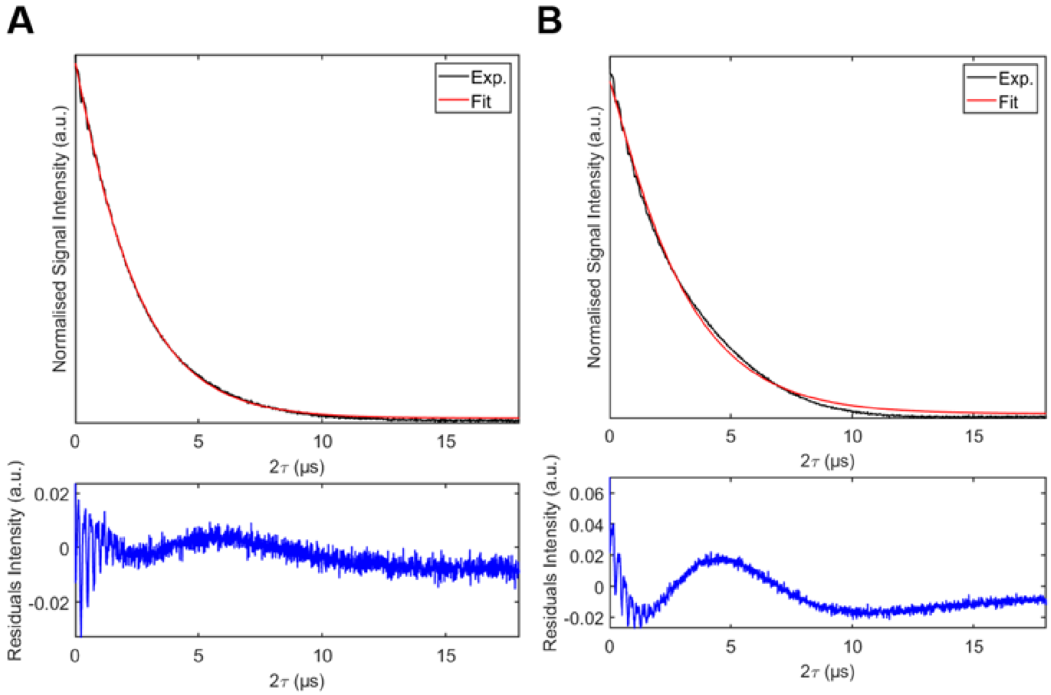

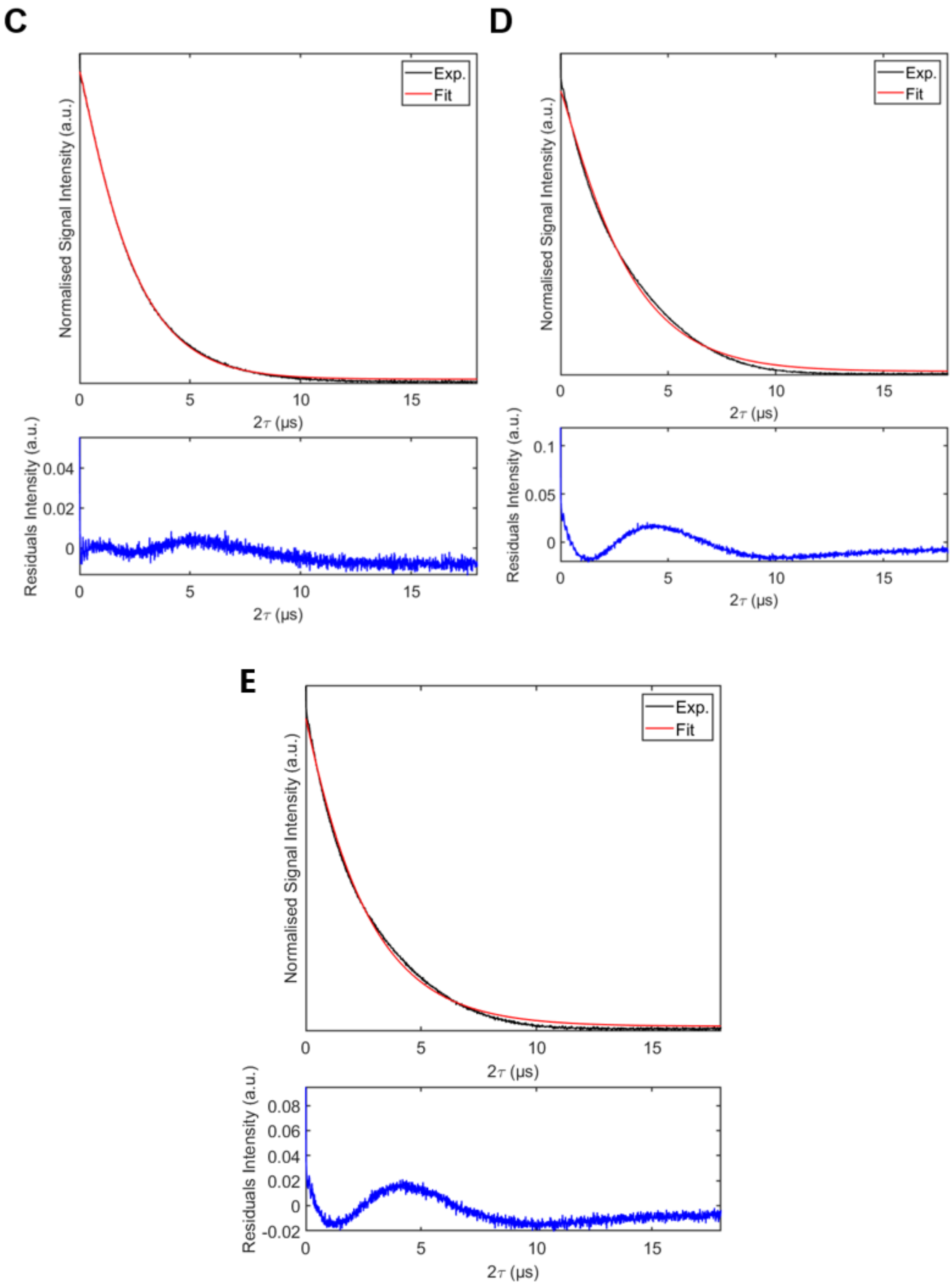

Figure S5. Phase memory time (T_m) measurement of **C8-OTEMPO** (0.2 mM) at Q-band in **A)** [d_3-C_2C_1im][NTf_2] (black), fitted T_m = 2.46 μs, x = 1.08. **B)** [d_3-C_8C_1im][NTf_2] (black), fitted T_m = 3.05 μs, x = 1.10. **C)** [C_2C_1im][NTf_2] (black), fitted T_m = 2.40 μs, x = 1.11. **D)** [C_8C_1im][NTf_2] (black), fitted T_m = 3.07 μs, x = 1.12. **E)** [$C_8C_1C_1$im][NTf_2] (black), fitted T_m = 2.64 μs, x = 1.03. Corresponding stretched exponential fits (x = stretched exponent) (red), and residuals (blue).

Spin-labelled ILs

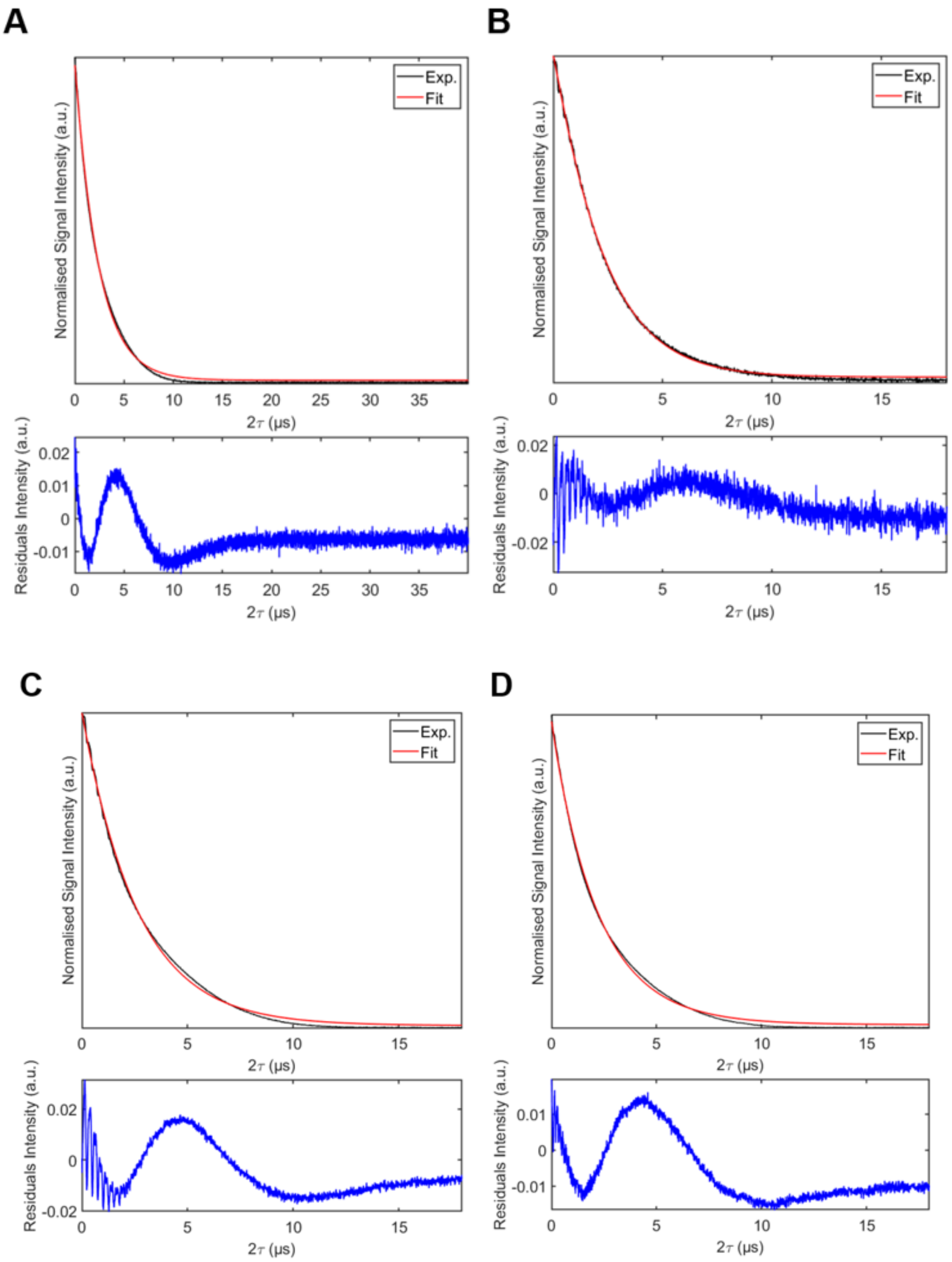

Figure S6. Phase memory time (T_m) measurement of **[ILC4][NTf$_2$]** (0.2 mM) at Q-band in **A)** [C_8C_1im][NTf$_2$] (black), fitted T_m = 2.40 μs, x = 1.02. **B)** [d$_3$-C_2C_1im][NTf$_2$] (black), fitted T_m = 2.36 μs, x = 1.08. **C)** [d$_3$-C_8C_1im][NTf$_2$] (black), fitted T_m = 2.62 μs, x = 1.01. **D)** [$C_8C_1C_1$im][NTf$_2$] (black), fitted T_m = 2.21 μs, x = 0.98. Corresponding stretched exponential fit (x = stretched exponent) (red), and residuals (blue).

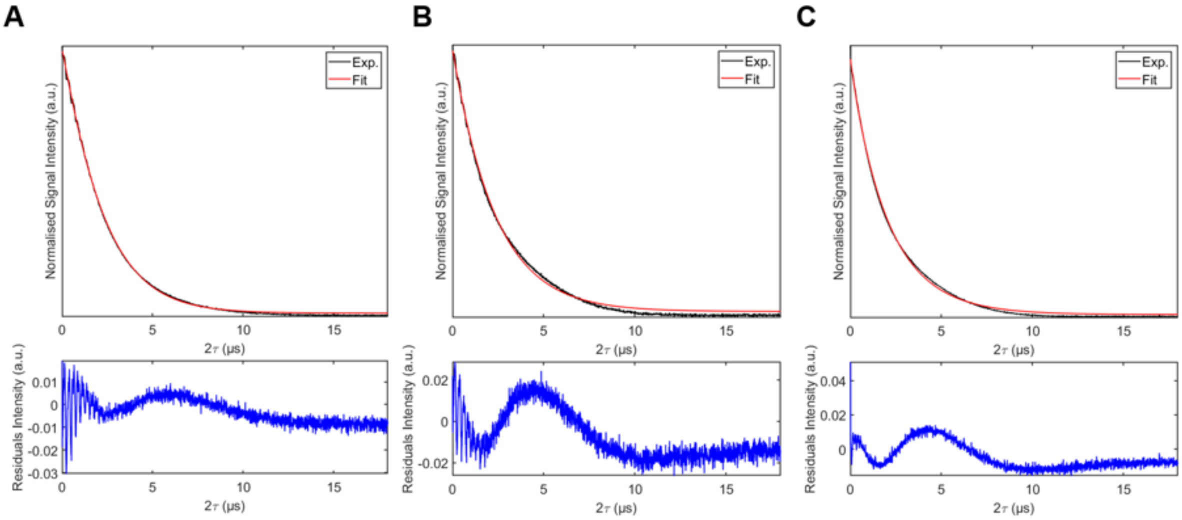

Figure S7. Phase memory time (T_m) measurement of **[ILC8][NTf$_2$]** (0.2 mM) at Q-band in **A)** [d$_3$-C_2C_1im][NTf$_2$] (black), fitted T_m = 2.32 μs, x = 1.09. **B)** [d$_3$-C_8C_1im][NTf$_2$] (black), fitted T_m = 2.35 μs, x = 1.02. **C)** [$C_8C_1C_1$im][NTf$_2$] (black), fitted T_m = 2.05 μs, x = 0.96. Corresponding stretched exponential fit (x = stretched exponent) (red), and residuals (blue).

^{19}F Mims Electron-Nuclear Double Resonance (ENDOR) at Q-band (50 K)

All ^{19}F Mims ENDOR spectra were recorded with π/2 = 6 ns, and RF_{π} = 105-110 µs, with a delay of 200 ns before, and 5 µs after, the RF pulse. The RF pulse was swept within a 1.8 MHz range centred around the corresponding ^{19}F Larmor frequency with a resolution of 3 kHz.

Spin-labelled ILs

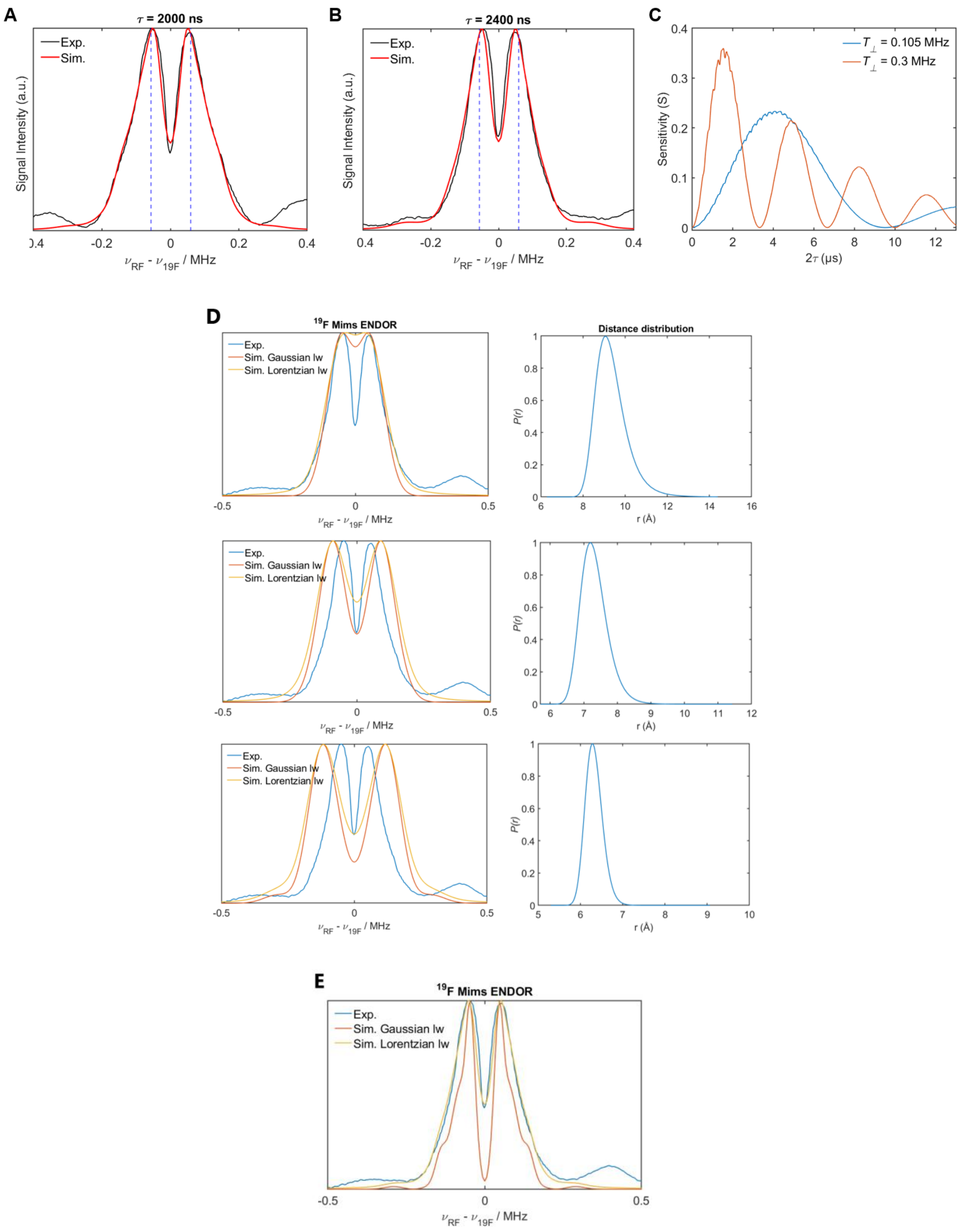

Figure S8. Experimental (black) and simulated (red) Q-band ^{19}F Mims ENDOR of **[ILC4][NTf$_2$]** in CD_2Cl_2/d8-toluene **A)** measured with τ = 2000 ns, **B)** measured with τ = 2400 ns. The summed measurement and simulation is shown in the main text (Figure 4A). **C)** Calculated sensitivity at 50 K of the ENDOR line for both smaller (blue) and large magnitude (orange) mean values of $T_\perp$, as determined by simulation of the experimental data, according to the equation: $S = F\,x\,I$, where *S* is sensitivity, *F* is ENDOR efficiency, and *I* is the echo intensity, where: $F = 0.5\,sin^2\left(\frac{2\pi\tau T_\perp}{2}\right)$ for a spin-1/2 centre. **D)** ^{19}F ENDOR simulations, overlayed on the experimental spectrum as it appears in the main text, Figure 4, A, considering a single distribution of hyperfine coupling tensors, and the corresponding distance distributions, for different mean values of $T_\perp$, the dipolar coupling constant, 0.1 MHz ± 0.02 (top), 0.2 MHz ± 0.03 (middle), 0.3 MHz ± 0.028 (bottom) with Gaussian linewidth broadening (0.1 mT), or this linewidth convoluted to a Lorentzian (Full-Width Half Maximum (FWHM) of a convoluted Lorentzian lineshape, 20 KHz, where the abscissa step was set to 4). The simulated data has been rescaled to the maximum of the experimental data. **E)** ^{19}F ENDOR measurement (blue) and simulation (yellow) as shown in the main text (Figure 4A), showing the Gaussian linewidth broadening of the summed simulated spectra, 0.05 mT (orange).

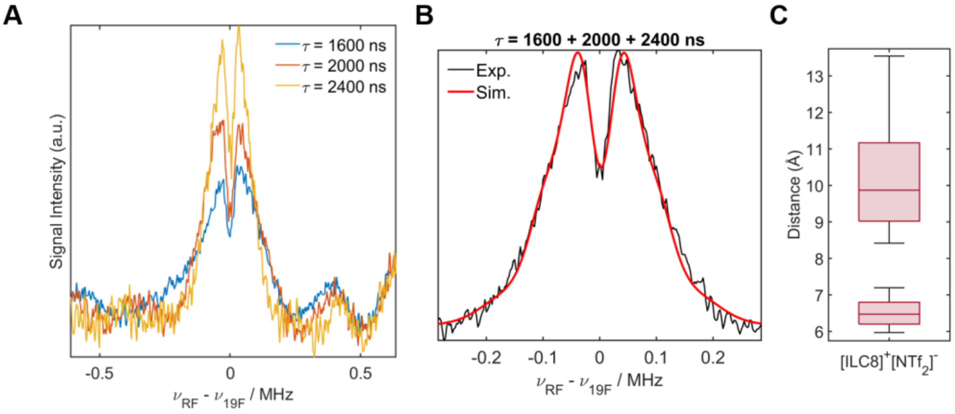

Figure S9. Experimental Q-band ^{19}F Mims ENDOR of **[ILC8][NTf$_2$]** in CD_2Cl_2/d8-toluene **A)** individual τ-value measurements, **B)** experimental τ-summed (black) and simulated (red) ENDOR spectrum, as described previously (details in Table 1, main text). **C)** Box and whisker plot of the distances determined by simulation of the experimental spectrum according to Equation 1 (main text), where the centre line of each box corresponds to the median nitroxide-^{19}F distance, $\tilde{r}$, the edges of the box correspond to the upper and lower quartiles, and the whiskers correspond to the maximum and minimum distances included in the simulation.

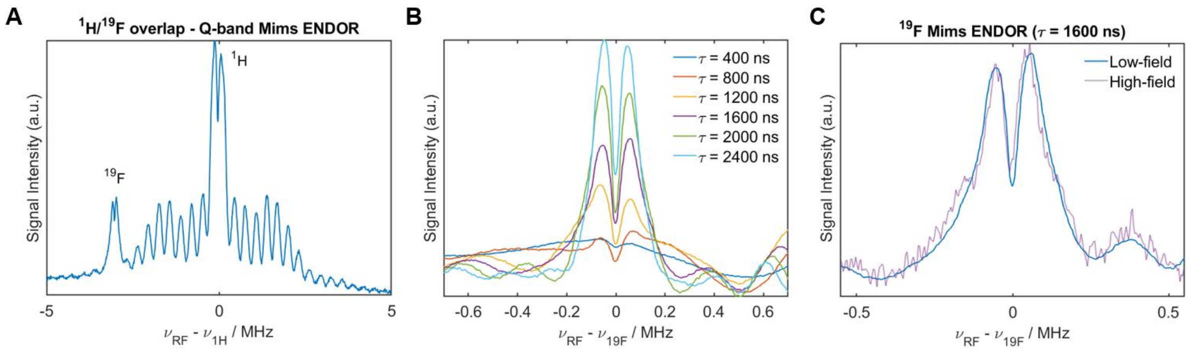

Figure S10. Q-band ^{19}F Mims ENDOR of **[ILC4][NTf$_2$]** in CD_2Cl_2/d8-toluene, **A)** showing the overlap of the ^{1}H and ^{19}F resonances at when detecting at the maximum of the nitroxide EDFS spectrum at Q-band and 1.1896 T, **B)** individual τ-value measurements, **C)** measured at both the maximum of the nitroxide EDFS spectrum (low-field) and at the edge of the high-field line ($m_I = -1$) measured with π/2 = 10 ns and with τ = 1600 ns.

^{19}F Mims ENDOR measurements of **[ILC4][NTf$_2$]** were attempted in CD_3CN, to corroborate the solvent dependent PRE-derived distances, however neat CD_3CN does not form a good glass and is a lossy solvent, making EPR measurements challenging. This was nonetheless attempted, despite clear distortions of the EDFS spectrum and the diminished phase memory time (T_m). The measured variable-τ ^{19}F Mims ENDOR spectrum showed no obvious ^{19}F signal.

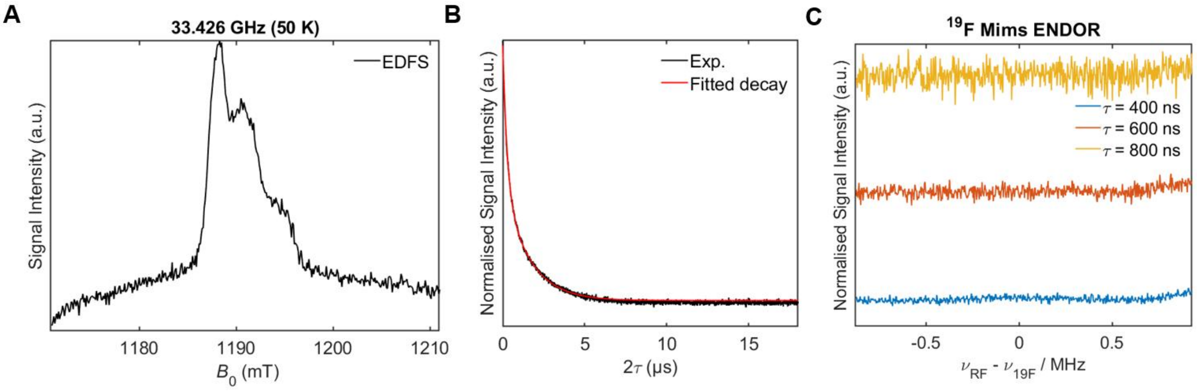

Figure S11. Measurements of **[ILC4][NTf$_2$]** in neat CD_3CN **A)** EDFS (τ = 320 ns), **B)** T_m detecting on the maximum of the nitroxide spectrum fit to a biexponential decay, **C)** Q-band variable-τ ^{19}F Mims ENDOR detecting on the maximum of the nitroxide spectrum.

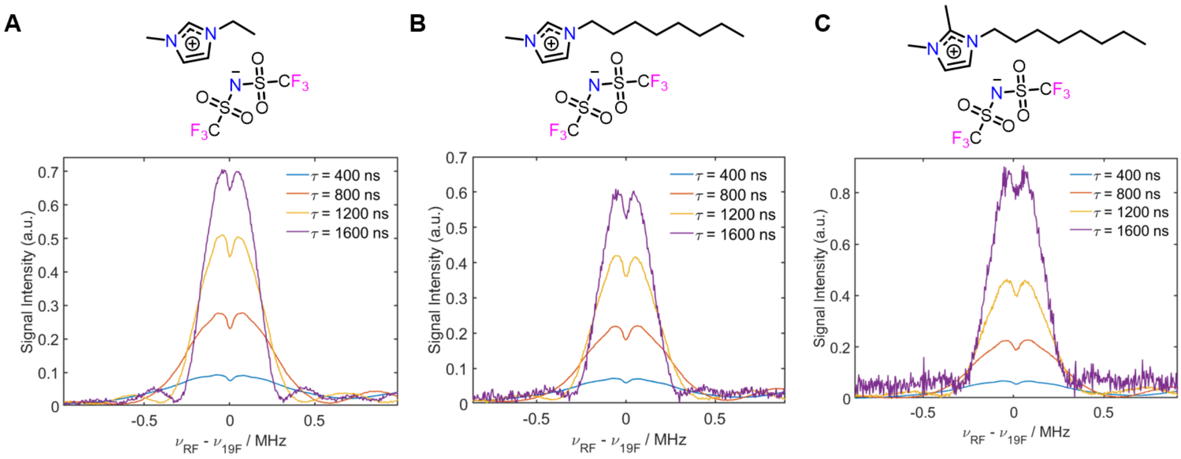

Figure S12. Q-band ^{19}F Mims ENDOR measurements of **[ILC4][NTf$_2$]** in **A)** [C_2C_1im][NTf_2], **B)** [C_8C_1im][NTf_2] and **C)** [$C_8C_1C_1$im][NTf_2]. The τ-summed measurements are discussed in the main text (Figure 5A).

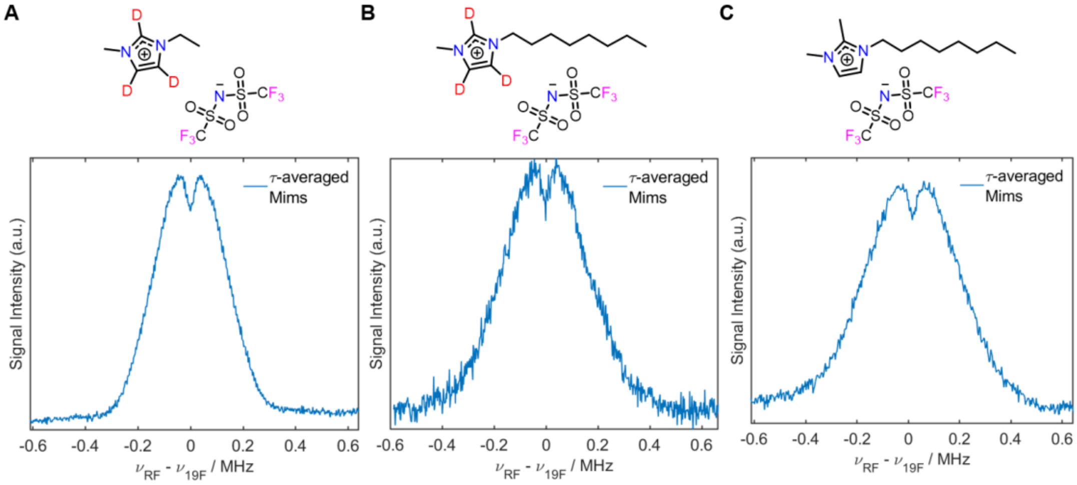

Figure S13. Q-band ^{19}F Mims ENDOR measurements of **[ILC8][NTf2]** in **A)** [d_3-C_2C_1im][NTf_2], **B)** [d_3-C_8C_1im][NTf_2], **C)** [$C_8C_1C_1$im][NTf_2].

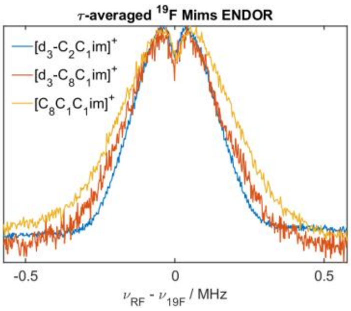

Figure S14. Normalised Q-band ^{19}F Mims ENDOR measurements of **[ILC8][NTf2]** in [d_3-C_2C_1im][NTf_2] (blue), [d_3-C_8C_1im][NTf_2] (orange) and [$C_8C_1C_1$im][NTf_2] (yellow). Marginally stronger interactions are observed as the level of imidazolium ring alkylation is increased, however uncertainties in the normalisation procedure and in the background signals at the edge of the spectra due to unavoidable ^1H overlap makes a definitive analysis difficult.

Spin probes

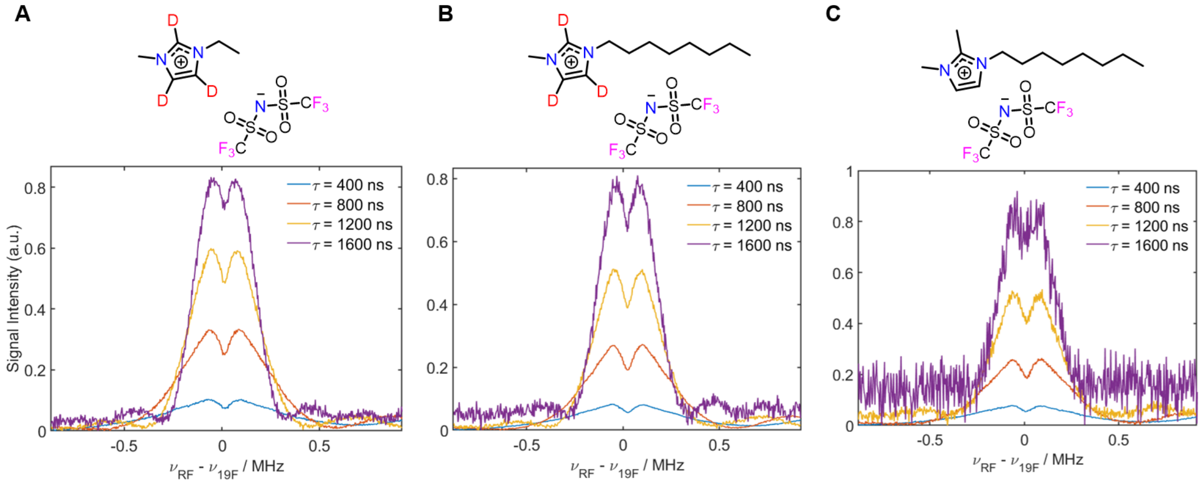

Figure S15. Q-band ^{19}F Mims ENDOR of **C8-OTEMPO** in **A)** [d_3-C_2C_1im][NTf_2], **B)** [d_3-C_8C_1im][NTf_2] and **C)** [$C_8C_1C_1$im][NTf_2]. The τ-summed measurements are discussed in the main text (Figure 5, B).

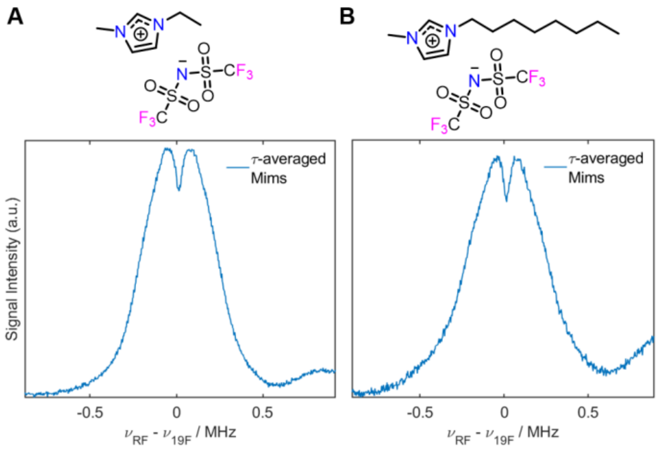

Figure S16. Q-band [19]F Mims ENDOR of a 2mM solution of **TEMPOL** in **A)** [C_2C_1im][NTf_2] and **B)** [C_8C_1im][NTf_2].

^{1}H Mims Electron-Nuclear Double Resonance (ENDOR) at Q-band (50 K)

All ^{1}H Mims ENDOR spectra were recorded with π/2 = 6 ns, and RF_π = 40-50 μs, with a delay of 200 ns before, and 5 μs after, the RF pulse. The RF pulse was swept within a 3-4 MHz range centred around the corresponding ^{1}H Larmor frequency with a resolution of 5 kHz.

Spin-labelled ILs

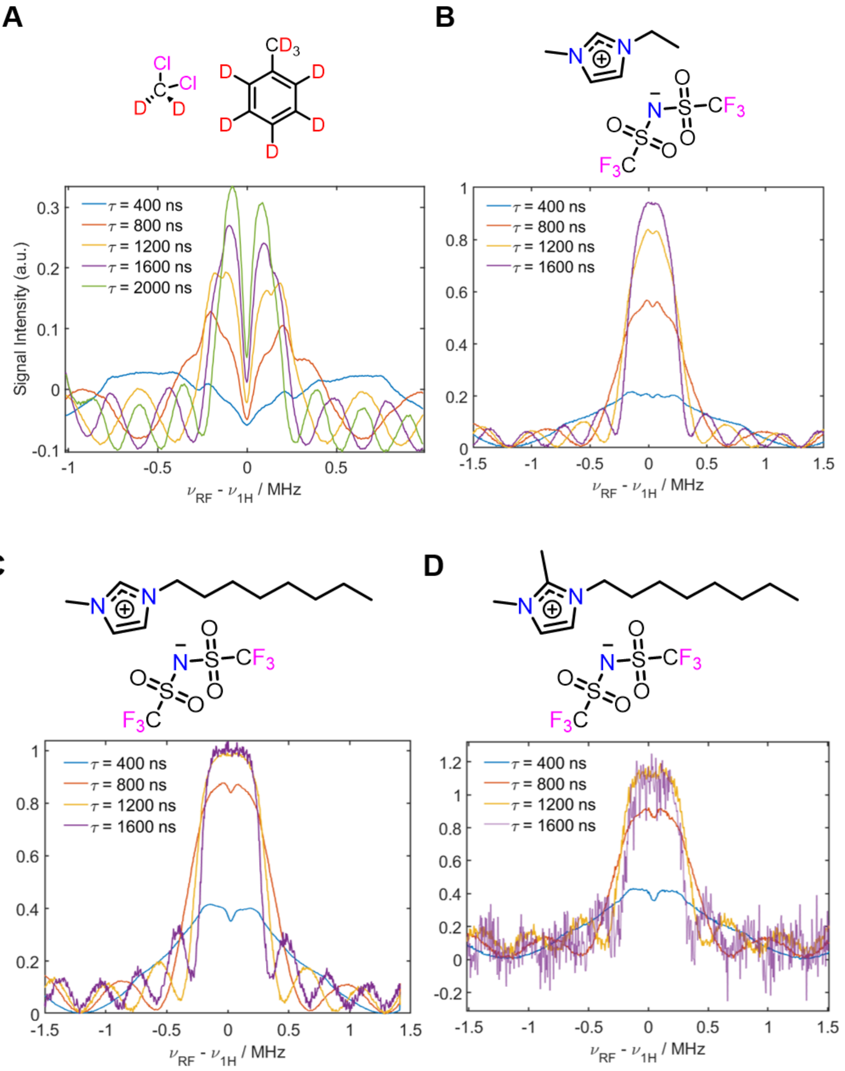

Figure S17. Q-band ^{1}H Mims ENDOR of **[ILC4][NTf2]** dissolved in **A)** CD_2Cl_2/d_8-toluene, **B)** [C_2C_1im][NTf_2], **C)** [C_8C_1im][NTf_2], **D)** [$C_8C_1C_1$im][NTf_2].

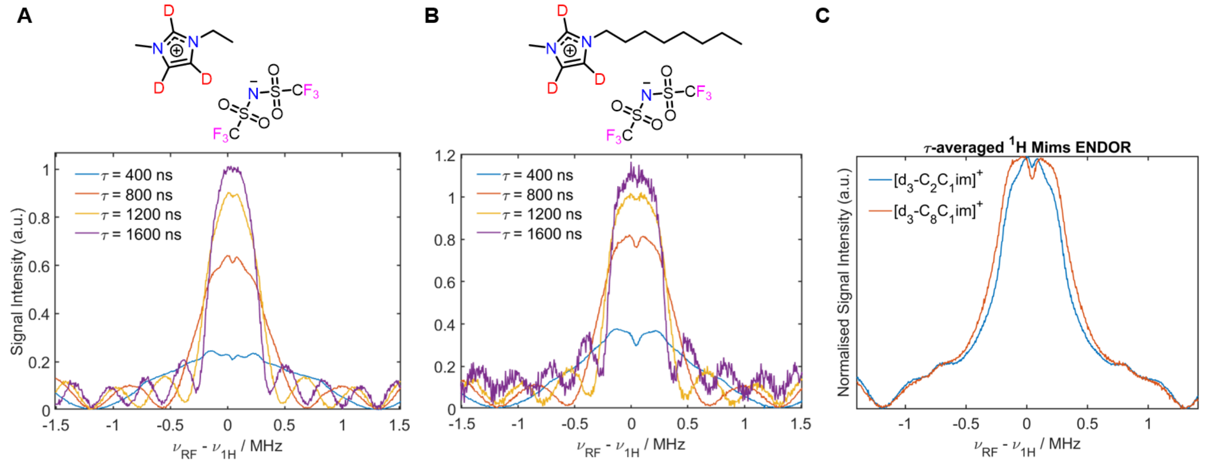

Figure S18. Q-band [1]H Mims ENDOR of **[ILC4][NTf2]** dissolved in **A)** [d_3-C_2C_1im][NTf_2], **B)** [d_3-C_8C_1im][NTf_2]. **C)** Q-band, τ-averaged, [1]H Mims ENDOR of **[ILC4][NTf2]** in perdeuterated ILs.

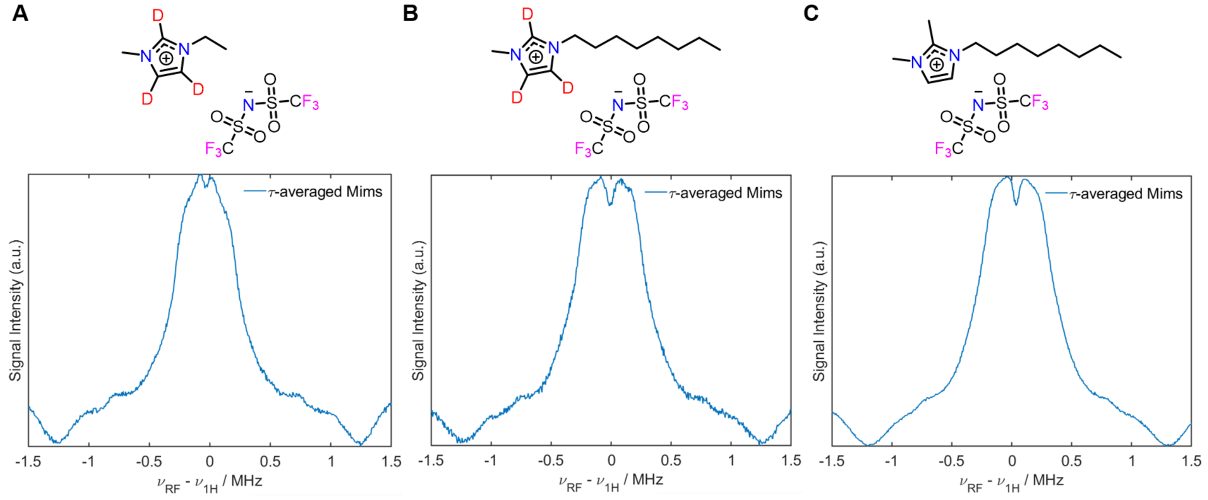

Figure S19. Q-band, τ-averaged, [1]H Mims ENDOR of **[ILC8][NTf2]** dissolved in **A)** [d_3-C_2C_1im][NTf_2], **B)** [d_3-C_8C_1im][NTf_2], **C)** [$C_8C_1C_1$im][NTf_2].

Spin probes

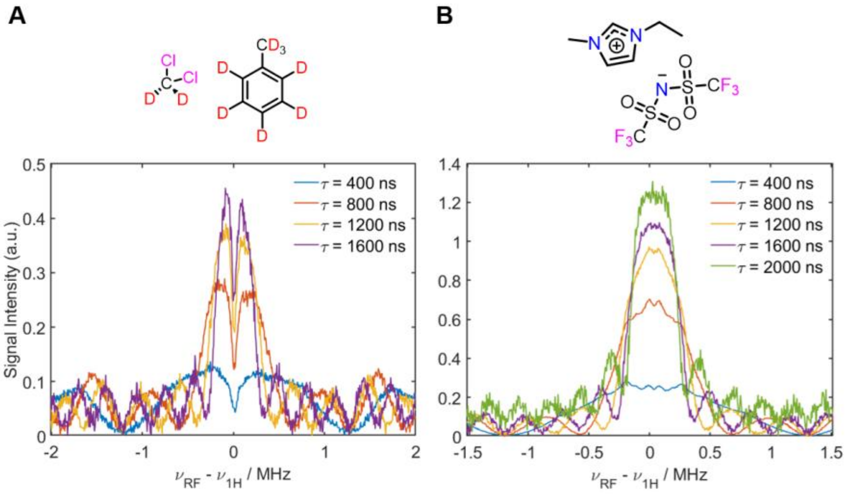

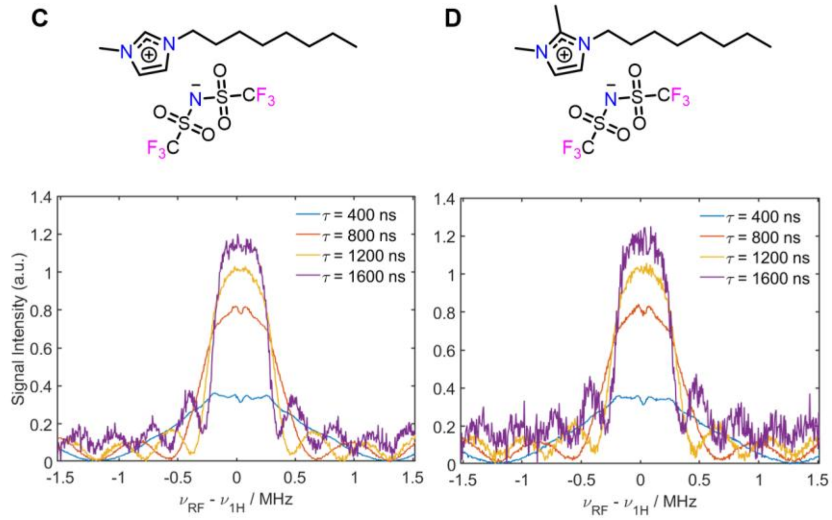

Figure S20. Q-band [1]H Mims ENDOR of **C8-OTEMPO** dissolved in **A)** CD_2Cl_2/d_8-toluene, **B)** $[C_2C_1im][NTf_2]$, **C)** $[C_8C_1im][NTf_2]$, **D)** $[C_8C_1C_1im][NTf_2]$.

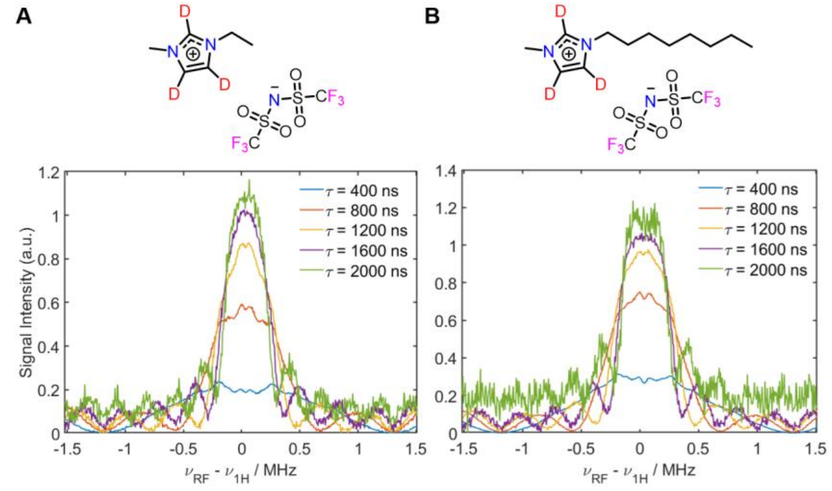

Figure S21. Q-band [1]H Mims ENDOR of **C8-OTEMPO** dissolved in **A)** $[d_3\text{-}C_2C_1im][NTf_2]$, **B)** $[d_3\text{-}C_8C_1im][NTf_2]$. **C)** Q-band, τ-averaged, [1]H Mims ENDOR of **C8-OTEMPO** in perdeuterated ILs.

Hyperfine Sublevel Correlation Spectroscopy (HYSCORE) at X-band (50 K)

All 4-pulse HYSCORE spectra were recorded with π/2 = 8 ns and an inversion pulse, π = 8 ns (amplitude tuned for inversion), with τ = 200 ns, $t_1 = t_2$ = 64 ns, and dx = dy = 8 ns. The time domain data was processed and analysed using Hyscorean,[7] where the signal was background corrected using a 3rd order polynomial, zero-filled, and apodized with a Hamming window before FFT of the spectrum and symmetrisation along the diagonal. The contour levels were set to 15-40% (min-max). 6-pulse HYSCORE spectra were analysed and processed in the same manner and were recorded with π/2 = 8 ns, π = 16 ns, and an inversion pulse, π = 8 ns (amplitude tuned for inversion), with $\tau_1 = \tau_2$ = 200 ns or with $\tau_1 = \tau_2$ = 140 ns, and $t_1 = t_2$ = 64 ns, and dx = dy = 8 ns.

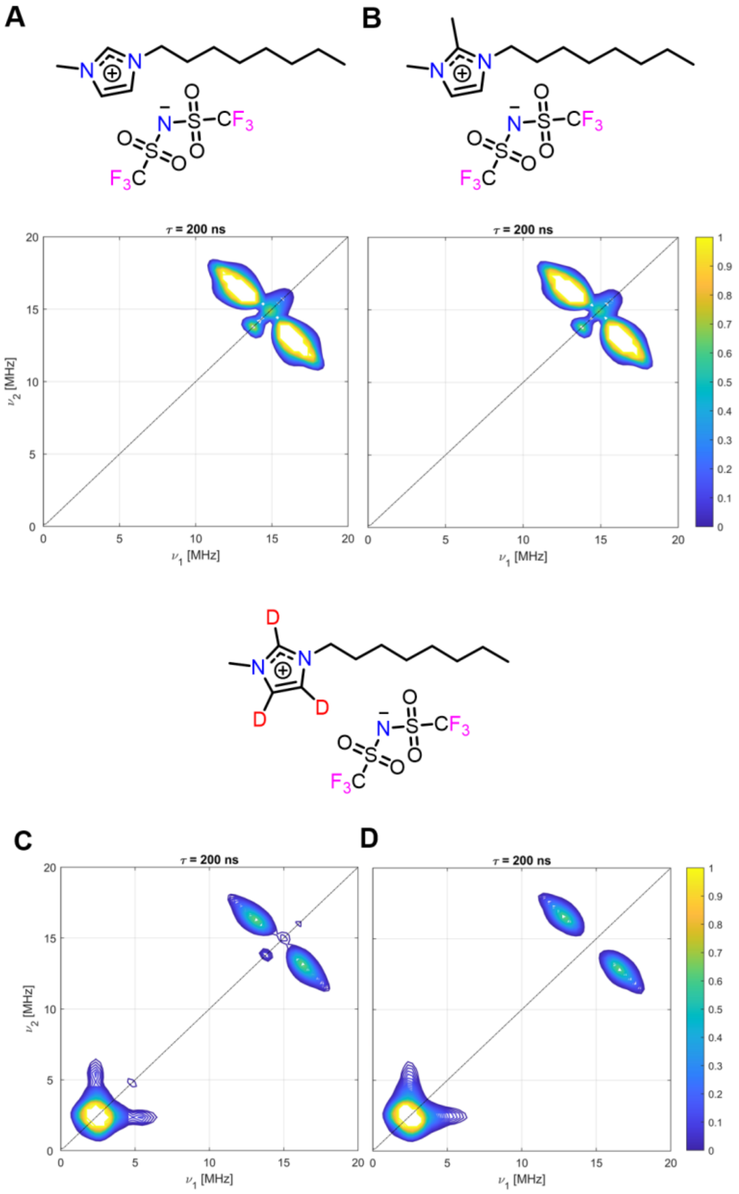

Figure S22. X-band 4-pulse HYSCORE spectrum showing the (+,+) quadrant of **[ILC4][NTf$_2$]** dissolved in **(A)** [C_8C_1im][NTf_2], **B)** [$C_8C_1C_1$im][NTf_2], **C)** [d_3-C_8C_1im][NTf_2]. **D)** X-band 6-pulse HYSCORE spectrum showing the (+,+) quadrant of **[ILC4][NTf$_2$]** dissolved in [d_3-C_8C_1im][NTf_2].

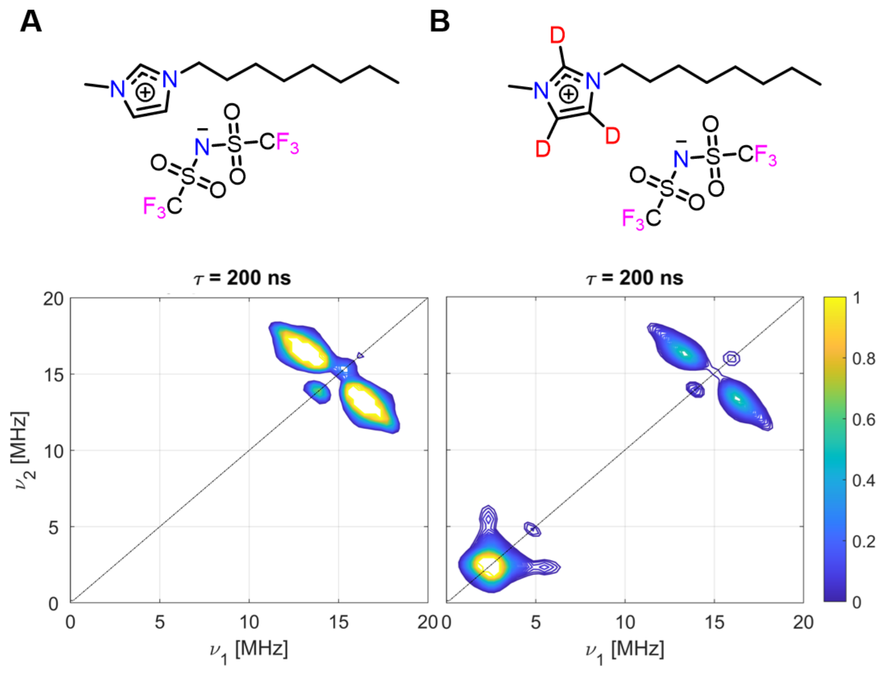

Figure S23. X-band 4-pulse HYSCORE spectrum showing the (+,+) quadrant of **C8-OTEMPO** dissolved in **(A)** [C_8C_1im][NTf_2], **B)** [d_3-C_8C_1im][NTf_2].

^{2}H Mims Electron-Nuclear Double Resonance (ENDOR) at Q-band (50 K)

All ^{2}H Mims ENDOR spectra were recorded with π/2 = 6 ns, and RF_π = 160 μs, with a delay of 200 ns before, and 5 μs after, the RF pulse. The RF pulse was swept within a 2.5 MHz range centred around the corresponding ^{2}H Larmor frequency with a resolution of 6 kHz or within a 1.8 MHz range with a resolution of 4 KHz.

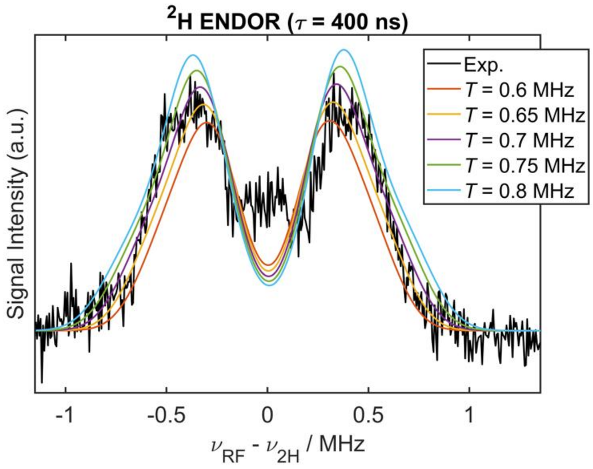

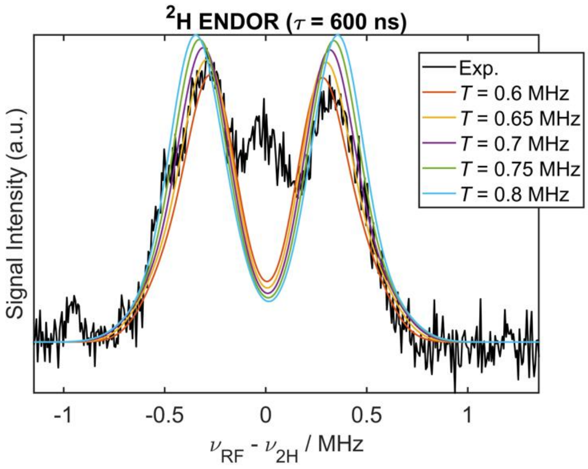

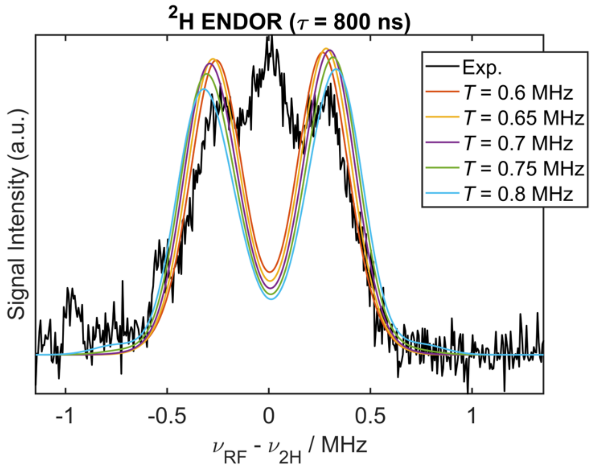

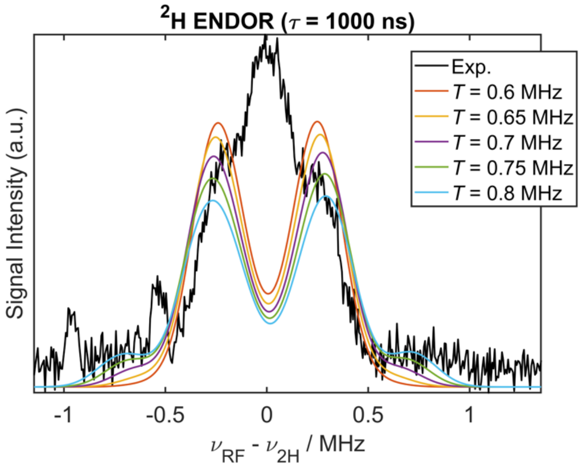

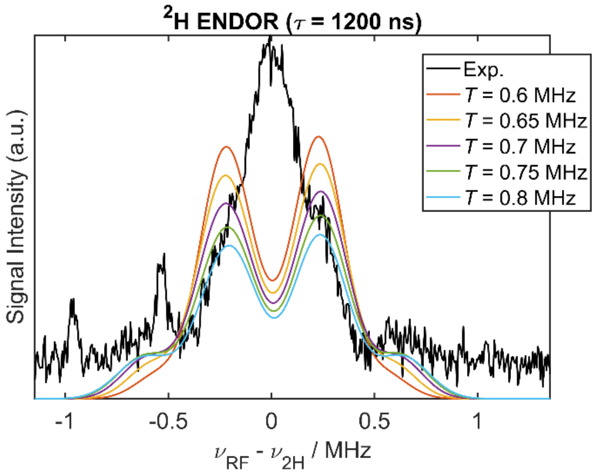

Figure S24. Q-band (50 K) τ-dependent ^{2}H ENDOR data **[ILC4][NTf$_2$]** (0.2 mM) in [d$_3$-C$_2$C$_1$im][NTf$_2$] as presented in the main text (black) and corresponding 'saffron' simulations assuming a purely dipolar interaction (*i.e.* A = [-T, -T, 2T]), with the ENDOR linewidth set to 0.2 mT, for different values of T leading to reasonable fits of the experimental spectra. The extra signals appearing at 0.55 and 0.95 MHz are likely due to distortions of the RF amplification due to the necessity to use high powered and long pulses to achieve the optimum flip angle for the Mims experiment. In the main text (Fig. 8,a), simulations considering T = 0.6, 0.7 and 0.8 MHz were summed and weighted in a 1:1:2 ratio to give the final simulated spectra.

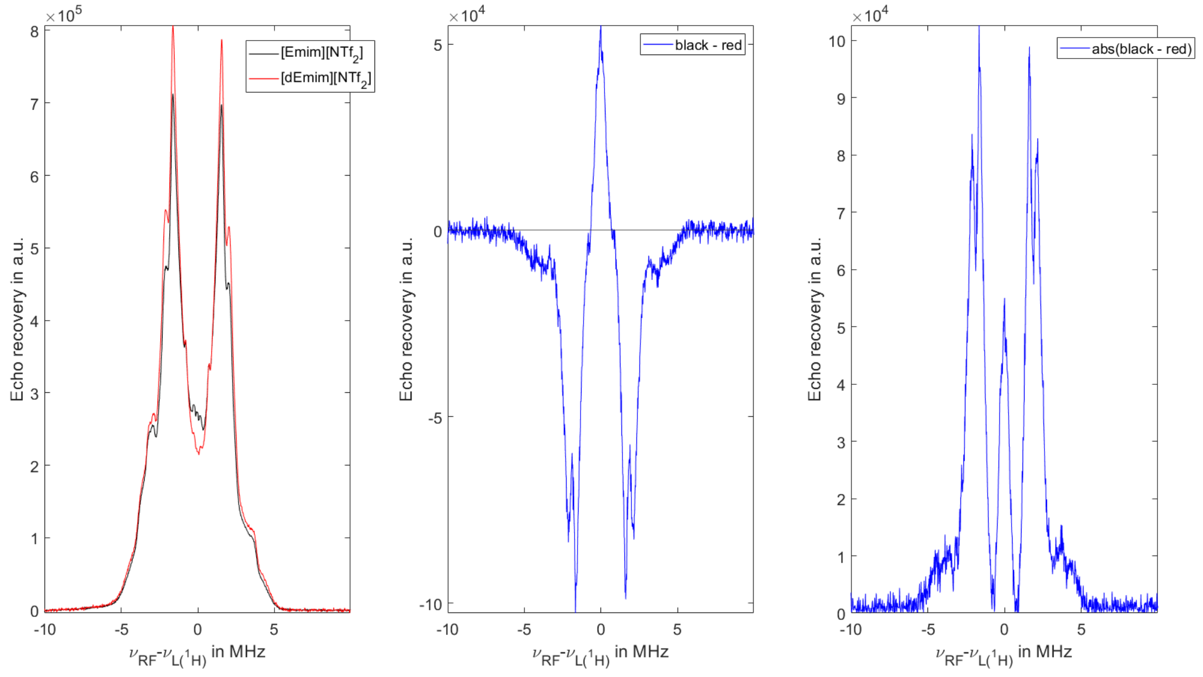

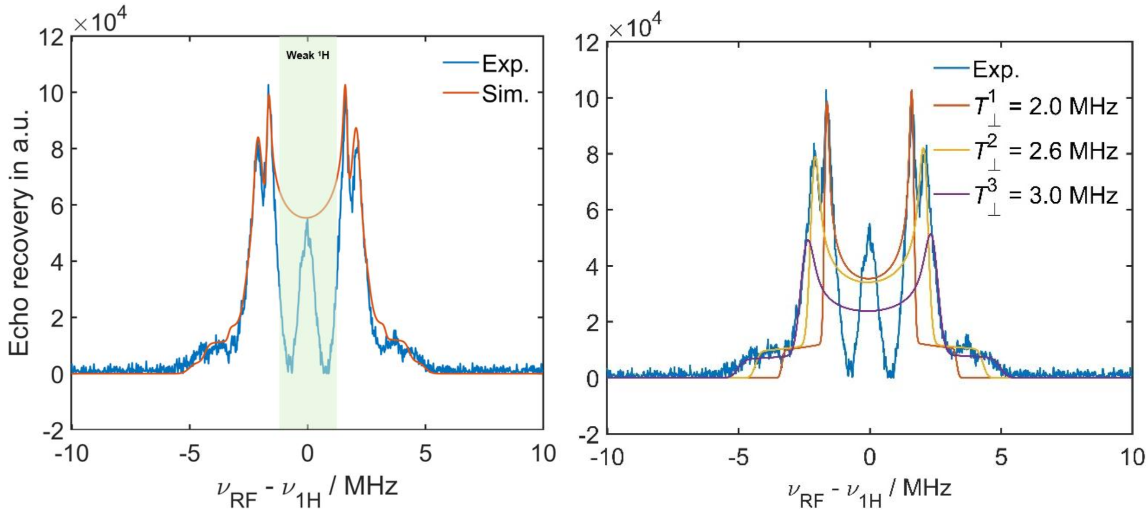

Figure S25. Q-band ^{1}H Davies ENDOR difference spectra of protonated vs deuterated samples.

(**Top**) (*Left*) Q-band Davies ENDOR measurements of **TEMPOL** (2 mM) at 50 K in protonated (black), and deuterated (red), [C$_2$C$_1$im][NTf$_2$], scaled by ENDOR efficiencies of the Mims ENDOR echo with protonated intensity (in a.u.) of RF$_{on}$ = 240,000, RF$_{off}$ = 300,000 and deuterated intensity (in a.u.) of RF$_{on}$ = 400,000, RF$_{off}$ = 460,000. (*Middle*) ^{1}H Davies ENDOR difference spectra (black trace subtracted from the red trace), (*Right*) ^{1}H Davies ENDOR difference spectra (absolute intensity of black trace subtracted from the red trace).

(**Bottom**) (*Left*) The ^{1}H Davies ENDOR difference spectrum (blue) as shown in (top, right) and summed simulation (orange) using the EasySpin function *salt.m* considering three purely dipolar (*i.e.* a_{iso} = 0 and $\boldsymbol{A} = [-T_\perp, -T_\perp, 2T_\perp]$ in MHz) hyperfine coupling interactions of ^{1}H with $T_\perp^{(1)}$ = 2.0, $T_\perp^{(2)}$ = 2.6 and $T_\perp^{(3)}$ = 3.0 MHz, with a relative weighting of 1.0:1.0:0.6, an ENDOR linewidth of 0.1, 0.2 and 0.3 mT respectively, and a GridSize of 91. (*Right*) Individual simulations plotted against the experimental difference spectrum.

Comments on the simulation of the Davies ENDOR difference spectrum (Figure S.3.29 bottom) and its interpretation

The starting values for the simulation were chosen based on the distributed values of $T_\perp$ obtained from the simulations of the ^{2}H Mims ENDOR experiments with their magnitudes scaled by the gyromagnetic ratio of the ^{1}H nucleus. A fit to a single value of $T_\perp$ could not reproduce the two sharp spectral peaks in the experimental data (blue). Moreover, the broadened ENDOR line width could not be fully accounted for using an inhomogeneous line broadening and was better reproduced by the addition of a third value of $T_\perp$. In the simulation shown (left, red trace) the contribution from $T_\perp^{(3)}$ = 3.0 MHz was scaled by 60% relative to those from $T_\perp^{(1)}$ = 2.0 and $T_\perp^{(2)}$ = 2.6 MHz, as this gave the best fit to the difference spectra upon calculation of the sum of squared residuals. The green shaded area designates the area of the spectrum that shows weak ^{1}H couplings and no attempt was made to simulate these; the differences here likely originate from the different ratios of ^{1}H and ^{2}H nuclei present in each sample beyond the first solvation sphere.

Simulation of the ^{1}H Davies ENDOR difference spectra suggests hydrogen bonding distances, under a point dipole approximation, of $T_\perp^{(1)}$: 2.7 Å, $T_\perp^{(2)}$ = 2.5 Å and $T_\perp^{(3)}$ = 2.3 Å (Fig. S.3.30). These distances are broadly in agreement with our ^{2}H Mims ENDOR analysis, when each of the three values of $T_\perp$ is scaled by the gyromagnetic ratio of the ^{2}H nuclear spin, which resulted in calculated hydrogen bonding distances of 2.2, 2.1 and 2.0 Å. The small discrepancy (in the worst case scenario) of 0.7 Å between the ^{1}H and ^{2}H analyses is likely a result of a weak isotope effect (generally a lighter isotope (^{1}H) will result in longer intermolecular hydrogen bonding lengths, while a heavier isotope (^{2}H) will result in shorter intermolecular deuterium bonding lengths),[23] and due to the assumption that the electronic spin density (ρ = 0.5) remains constant between the protonated and deuterated systems, as shown in the main text (Fig. 8b).

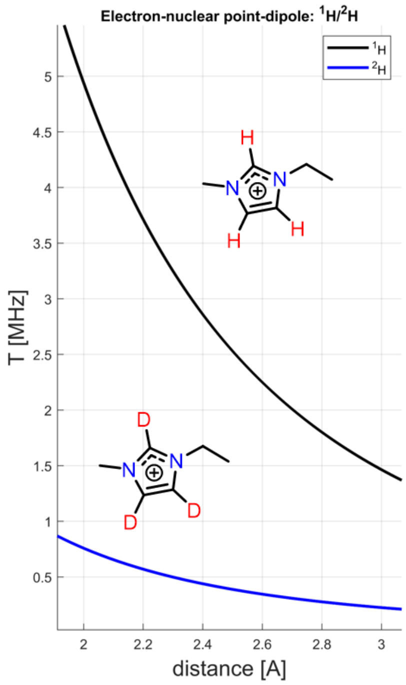

Figure S26. Calculated electron-^{2}H and electron-^{1}H distances, for a range of calculated values of *T* (MHz) under a point dipole model, for spin density ρ = 0.5, and corresponding imidazolium rings showing the ^{1}H and ^{2}H nuclear spins of interest.

We expect the **TEMPOL** to interact with three imidazolium ^{1}H nuclei, and this is consistent with the simulations discussed above (Fig. S25 bottom). However, in this soft-matter system we feel that it would be an over-interpretation to make a definitive assignment to nuclear centres based on the above-derived distances. This is because the simulation and therefore interpretation of ^{1}H Davies ENDOR difference spectra rests on how the subtraction of data is done. Firstly, it is noteworthy that assignment of *all* the proton couplings (*i.e.* without using subtraction) is not feasible due to the numerous ^{1}H nuclei present; indeed, there is no obvious additional ^{1}H coupling present in the protonated vs the deuterated sample (black vs red trace in Figure S.3.29, top, left). To obtain the best-possible difference spectrum (Fig. S25 bottom, blue trace), care was taken in recording undistorted ENDOR spectra (by performing a stochastic radiofrequency sweep rather than a progressive sweep and carefully calibrating the microwave and radiofrequency inversion pulses prior to acquisition), such that spectra could be scaled by ENDOR efficiencies. However, extracting ENDOR efficiencies from the experimental traces is problematic due to the effects of differing spin-lattice (T_1) relaxation times between samples. Previously these issues have been addressed by introducing an empirical and entirely subjective scaling factor (k).[24] We trialled this approach on our difference spectra, and while the peak positions remained approximately in the same position, this showed that the ^{1}H Davies ENDOR difference spectra cannot be interpreted unambiguously (Figure S27). Notably, because the electron-^{19}F interaction is not affected by sample deuteration, we would expect the ^{19}F peak to vanish following the subtraction. This situation results in *negative* ^{1}H peaks and hence a contradiction: the number of ^{1}H nuclei would be larger in a deuterated sample compared to a natural-abundance sample. Consequently, although the ^{1}H Davies ENDOR

difference spectra overall support interactions of the **TEMPOL** with three imidazolium ring protons, further structural interpretation is unwarranted.

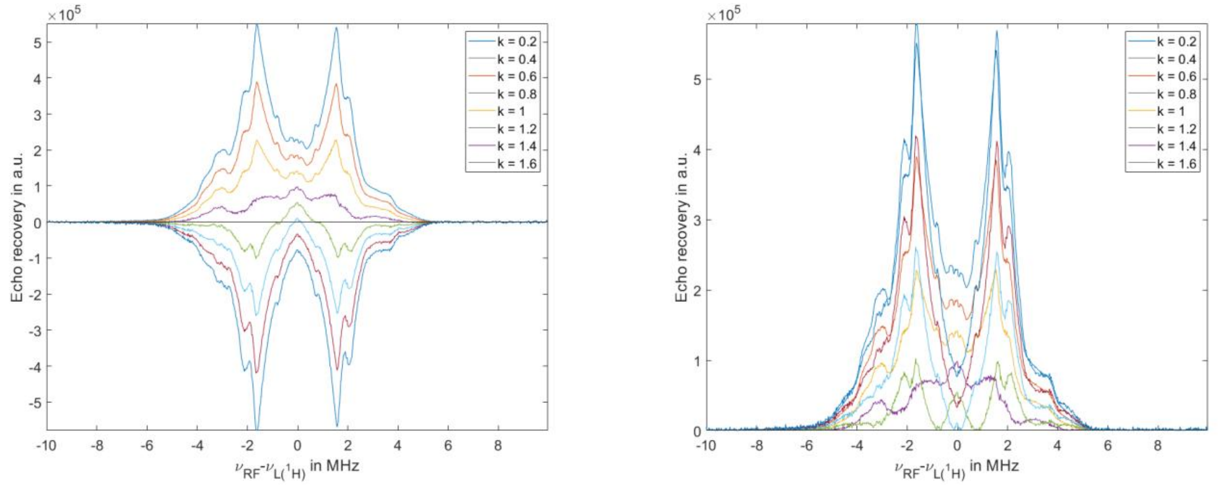

Figure S27. Davies ENDOR difference spectrum of the protonated and deuterated [C_2C_1im][NTf_2] ILs, as shown in Figure S.3.29 (Top), with (*left*) the protonated spectrum subtracted from the deuterated spectrum, and (*right*) the absolute intensity of the protonated spectrum subtracted from the deuterated spectrum, now scaled according to Z = X-Y*k,[23] where X = protonated sample, Y = deuterated sample, k = scaling factor (coloured).

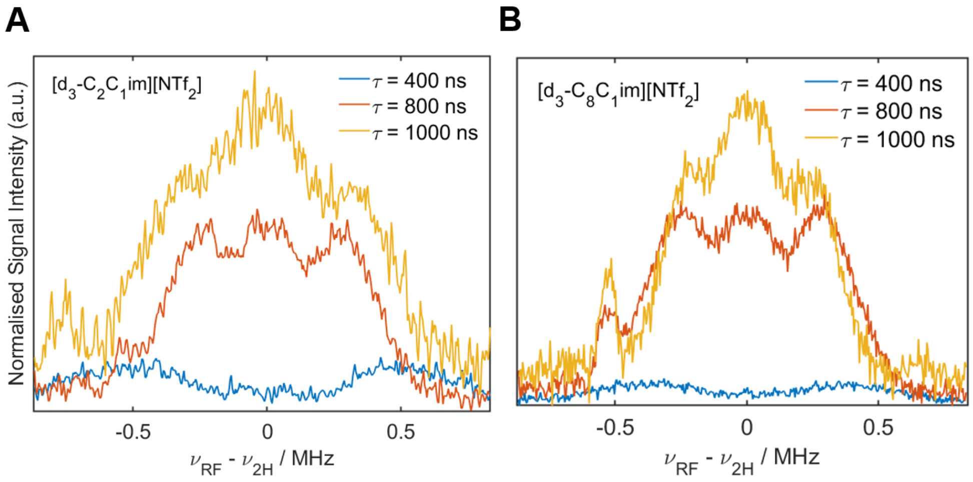

Figure S28. Q-band ^{2}H ENDOR measurements of **C8-OTEMPO** at different values of τ in **A)** [d_3-C_2C_1im][NTf_2], **B)** [d_3-C_8C_1im][NTf_2].

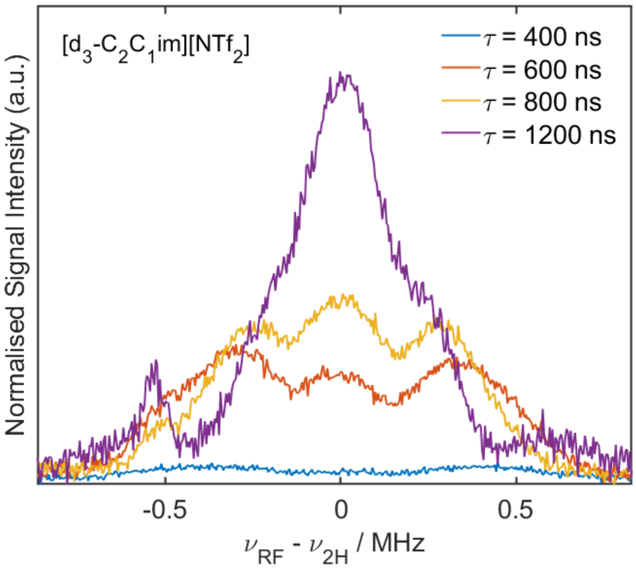

Figure S29. Q-band ^{2}H ENDOR measurements of **TEMPOL** (2 mM) at different values of τ in [d_3-C_2C_1im][NTf_2].

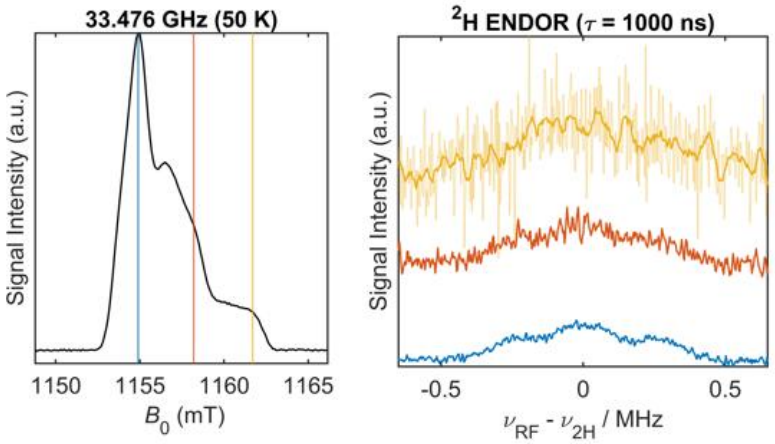

Figure S30. Q-band ^{2}H ENDOR measurements of **[ILC4][NTf$_2$]** (0.2 mM) at τ =1000 ns in [d_3-C_2C_1im][NTf_2] detecting at different field positions (coloured) of the EDFS spectrum, measured at 33.476 GHz (50 K).

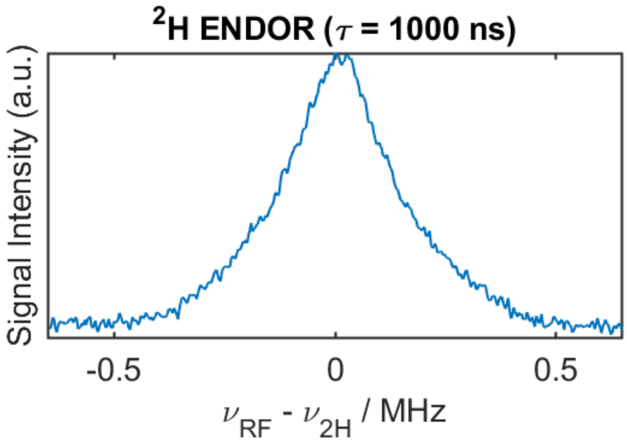

Figure S31. Q-band ^{2}H ENDOR measurements of **[ILC8][NTf$_2$]** (0.2 mM) at τ =1000 ns in CD_2Cl_2/d_8-toluene, measured at the maximum of the EDFS spectrum.

S.4 Computational methods

Density functional theory (DFT)

DFT calculations of **[ILC4][NTf$_2$]** and **[ILC8][NTf$_2$]** were carried out in ORCA V5.0.3,[8] taking the previously optimised structure of [C_4C_1im][NTf$_2$] as a starting point (Figure S.4.1). The [C_4C_1im][NTf$_2$] structure was optimised using Gaussian16.[9] The B3LYP functional was employed with 6-311+G(d,p) basis sets on all atoms. Grimmes D3 dispersion correction and Becke-Johnson dampening (GD3BJ)[10] was added. The starting structures were selected according to typical IL cation/anion orientations and the structure was then fully optimised. Frequency analysis was performed on the calculated minima to confirm the presence of no imaginary frequencies. To the optimised structure of [C_4C_1im][NTf$_2$] a nitroxide-based fragment was added, using Chemcraft (V.1.8).[11] The cation-anion bonding geometries were then constrained based off the starting structure, *i.e.* a top-interaction of the nitrogen atom of the anion with the 'C^2' proton of the cationic imidazolium ring, and the remaining unconstrained structure was then optimised. The optimisation calculations used unrestricted Kohn-Sham wavefunctions, the B3LYP functional, with 6-311+G(d,p) basis sets on all atoms, an RIJCOSX approximation, and a D3BJ dispersion correction. The convergence criteria was set to 'TIGHT', and the integration grid set to the default, 'defgrid2'. Geometry optimisations were performed considering explicit solvation using the conductor-like polarizable continuum model (CPCM), assuming the value of the dielectric constant of dichloromethane (DCM). The resulting optimised structures represent one of many possible lowest energy conformers due to the weakly-coordinating nature of the [NTf$_2$]$^-$ anion and the bias in the chosen starting structure cation-anion interaction. Molecules were visualised in Chimera.[12]

DFT optimised conformer of [C_2C_1im][NTf$_2$]

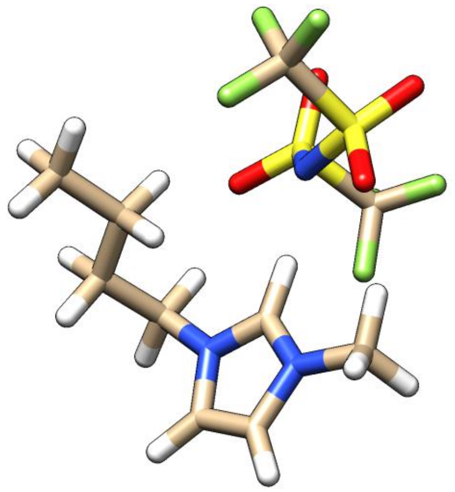

Figure S32. Geometry optimised structure of the 'top' conformer [C_4C_1im][NTf$_2$] used as the starting point for modelling and optimising the spin-labelled ILs. (Red – O, Green – F, Blue – N, Yellow – S, Beige – C, White – H).

DFT optimised conformer of ***[ILC8][NTf$_2$]***

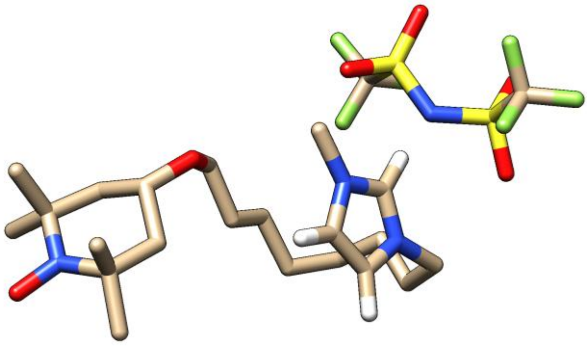

Figure S33. Geometry optimised structure of the 'top' conformer of **[ILC8][NTf$_2$]**. (Red – O, Green – F, Blue – N, Yellow – S, Beige – C, White – H). Non-imidazolium ring protons have been removed for clarity.

Molecular Dynamics (MD) simulations

To conduct MD simulations, polarizable MD was used employing the CL&Pol forcefield[13] for the ILs components, and parameters from a study by Salanne *et al.*[14] for the nitroxide radical components of the system. The LAMMPS software package[15] was used for the simulation with Packmol[16] used to prepare the starting box size based on the expected density of the neat [C_8C_1im][NTf_2] IL in all cases. Systems tested involved the 500 ion pairs of the pure spin-labelled cation, [ILC4]$^+$, with the [NTf_2]$^-$ counter-anion, and a 10% molar volume [ILC4]$^+$ in a [C_8C_1im][NTf_2] solution with a total of 500 ion pairs. In addition, a solution of 50 TEMPOL radicals with 500 ion pairs of [C_2C_1im][NTf_2], [C_2C_1im][BF_4] and [C_2C_1im][FSI] were also tested.

Simulations were run for 5 ns at 300 K using an NpT ensemble to equilibrate the system, while a production run of the equilibrated system was run for 10 ns also at 300 K using an NVT ensemble was used for data analysis. The Nosé–Hoover thermostat was used in all cases with a dump frequency of 1 ps, and a timestep of 1 fs during both equilibration and production. This is in line with prior simulations on ILs as well as nitroxide radicals and has been shown to yield adequate statistical sampling.[17,18]

Analyses presented were conducted using the TRAVIS software program[19] on the full production run with visualization presented using the VMD software package.[20] Radial distribution functions (RDFs) were calculated using a bin frequency of 0.33 pm with the center of mass (CoM) defined internally by TRAVIS. As per prior studies,[21] a script was used to find the first minima to use the integral before this point as the coordination number. Spatial distribution function (SDF) analysis was also performed with TRAVIS, with the reference being the 2 nitrogen atoms and the linking carbon in the case of imidazolium, the ortho and para position to the nitroxide group in the nitroxide ring, and the nitrogen and sulphur atoms in [NTf_2]$^-$. The isovalues for SDFs were adjusted to show the most prominent interaction site with these values listed below all images. The C_r hydrogen is labelled C_2, and the C_w hydrogen labelled C_4/C_5, in the main text. Probe imidazolium refers to the imidazolium ring of the spin-labelled IL, TEMPO refers to the nitroxide moiety of the spin-labelled IL.

System 1: *50 [ILC4]$^+$, 450 [C_8C_1im], 500 [NTf_2].*

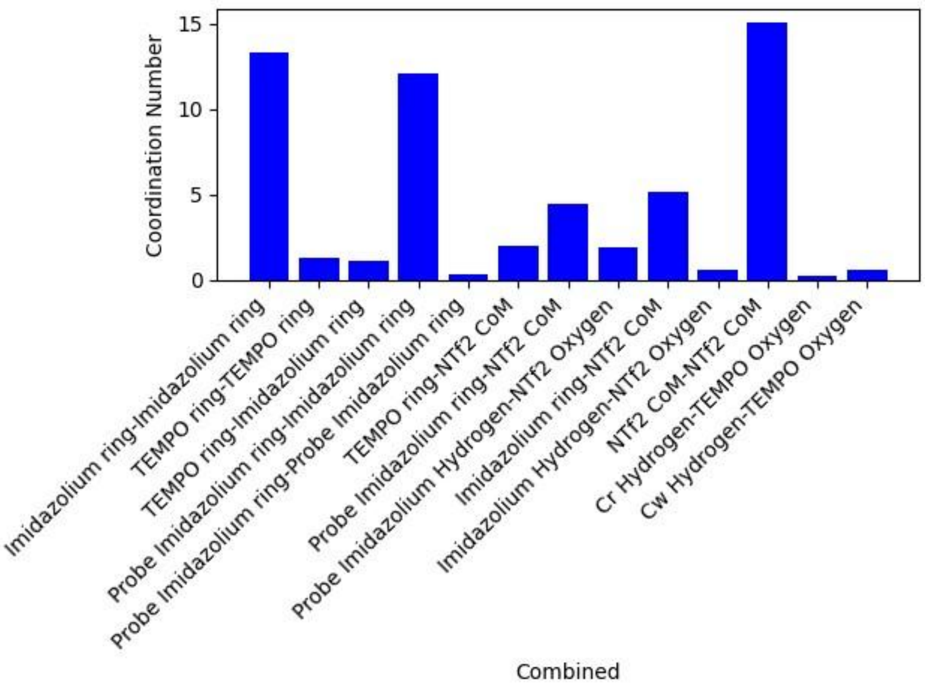

Figure S34. Coordination numbers determined from the Radial Distribution Functions (RDFs) of the MD simulation runs.

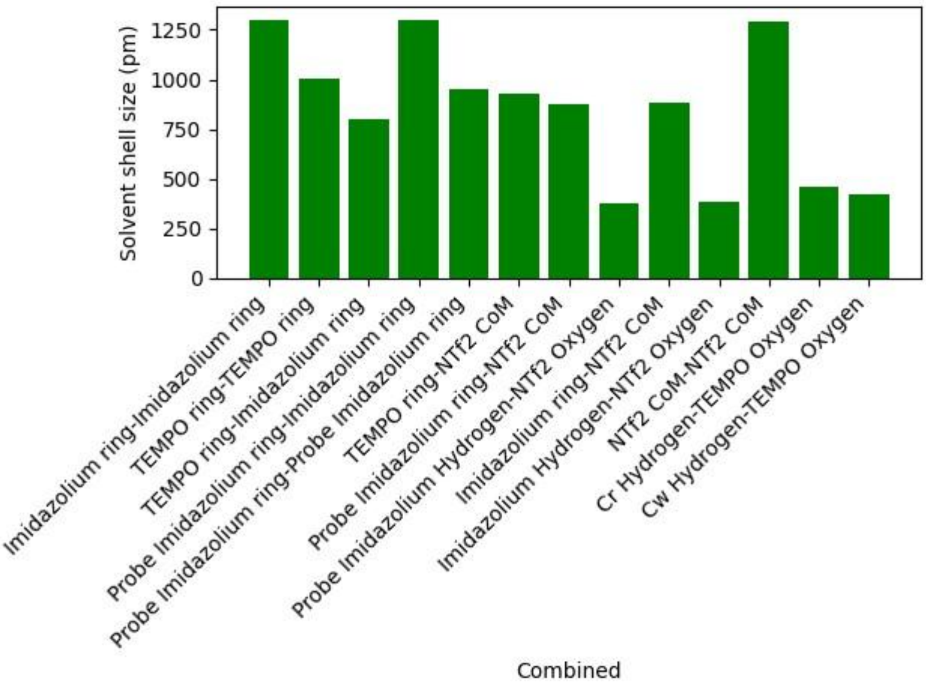

Figure S35. Solvent shell size determined from the Radial Distribution Functions (RDFs) of the MD simulation runs.

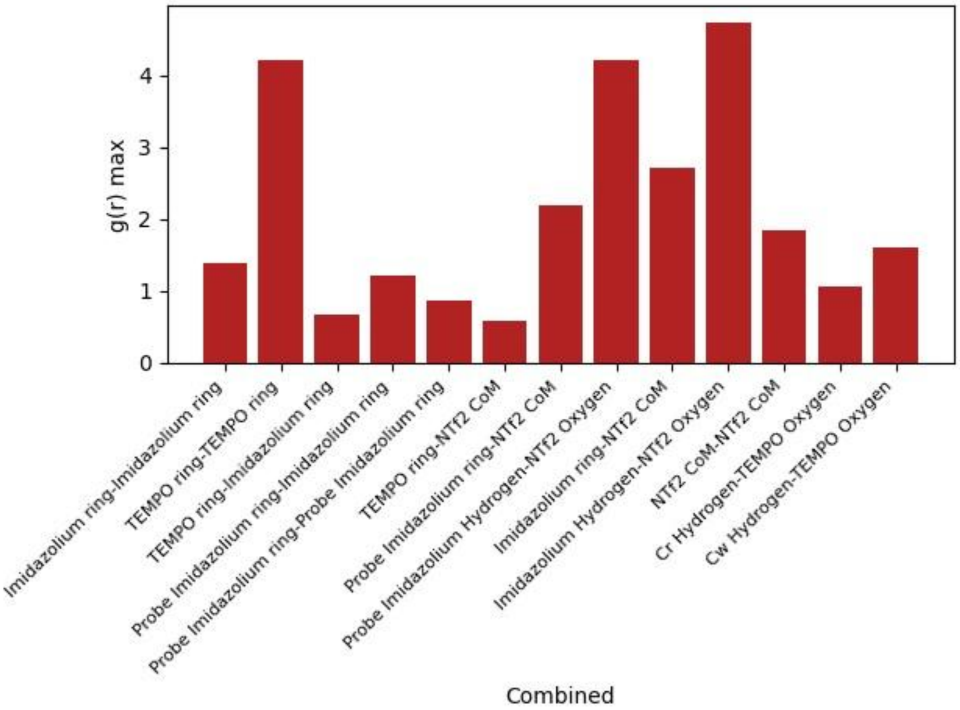

Figure S36. Specific interaction intensity, *g*(r), determined from the Radial Distribution Functions (RDFs) of the MD simulation runs.

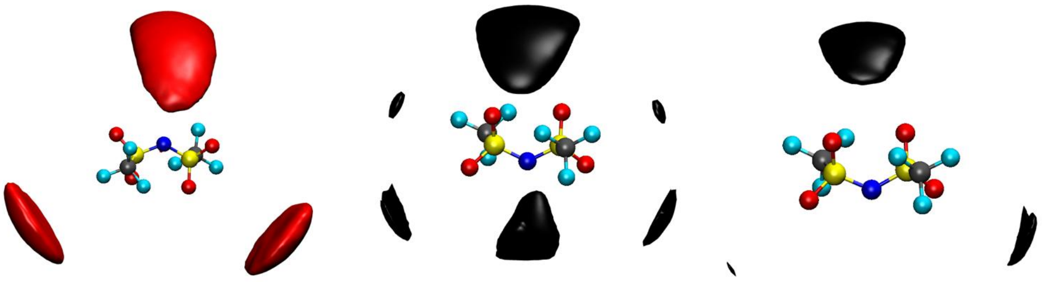

$[NTf_2]^-$-$[NTf_2]^-$ isovalue: 4

$[NTf_2]^-$-Imidazolium ring isovalue: 6

$[NTf_2]^-$-probe imidazolium ring isovalue: 0.7

Figure S37. Spatial Distribution Functions (SDFs): $[NTf_2]^-$ plots.

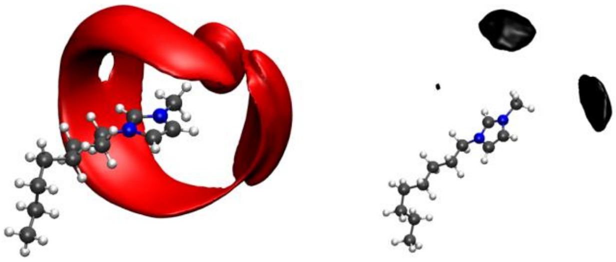

Imidazolium-$[NTf_2]^-$ isovalue: 7

Imidazolium-Imidazolium isovalue: 3

Figure S38. Spatial Distribution Functions (SDFs): Imidazolium plots.

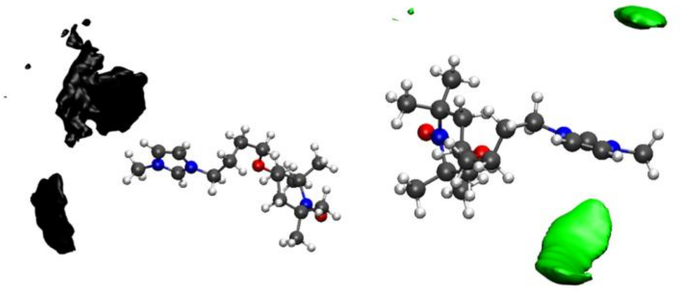

Probe Imidazolium – imidazolium isovalue: 2.75 **Probe Imidazolium–TEMPO** isovalue: 0.7

Figure S39. Spatial Distribution Functions (SDFs): Probe imidazolium plots.

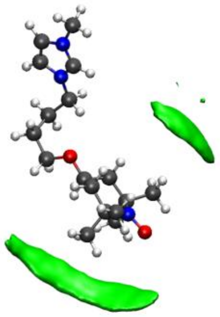

TEMPO– TEMPO
isovalue: 0.7

Figure S40. Spatial Distribution Functions (SDFs): TEMPO plots.

System 2: *500 [ILC4]$^+$, 500 [NTf$_2$].*

MD simulations of the all-spin labelled IL system show a favourable TEMPO-TEMPO interaction that is surprisingly high for a π-π interaction (g(r) = 2.46).

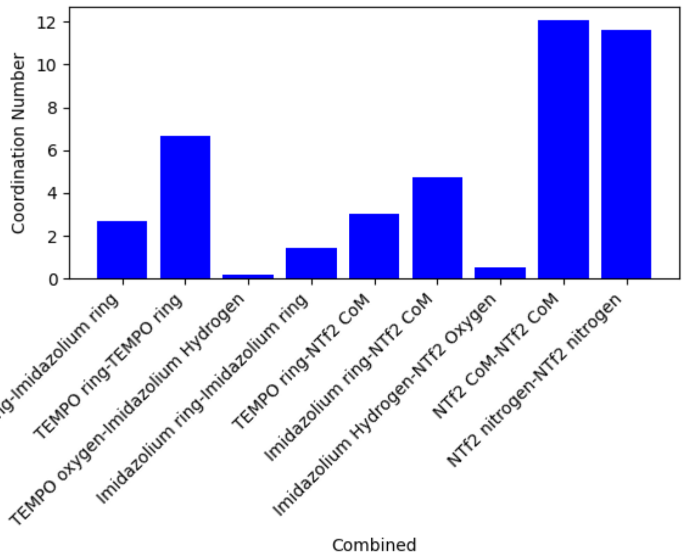

Figure S41. Coordination numbers determined from the Radial Distribution Functions (RDFs) of the MD simulation runs.

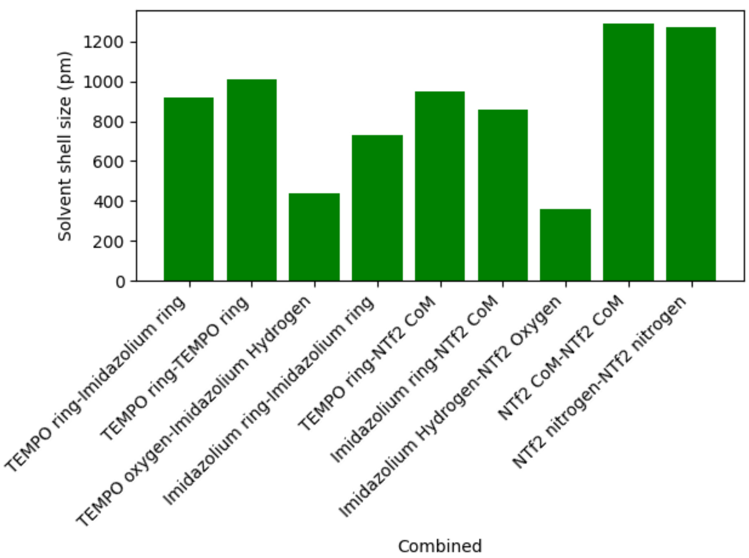

Figure S42. Solvent shell size determined from the Radial Distribution Functions (RDFs) of the MD simulation runs.

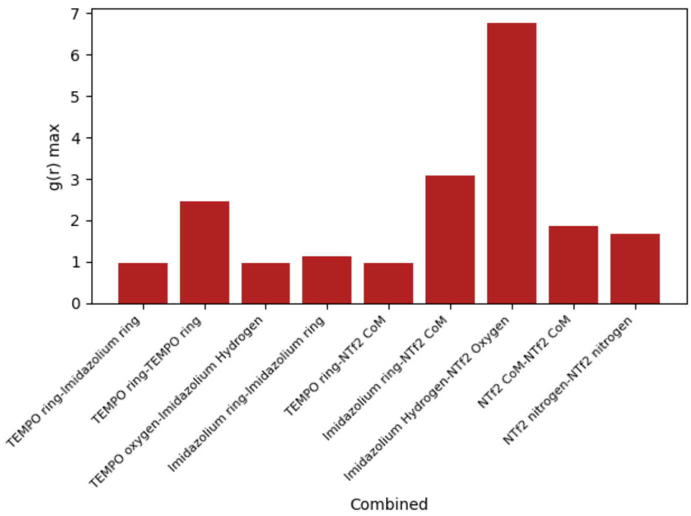

Figure S43. Specific interaction intensity, *g*(r), determined from the Radial Distribution Functions (RDFs) of the MD simulation runs.

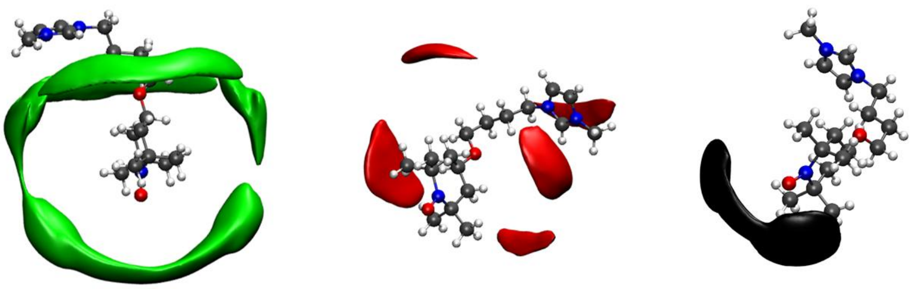

**TEMPO-TEMPO**	**TEMPO-[NTf$_2$]$^-$**	**TEMPO-Imidazolium**
isovalue: 5	isovalue: 3	isovalue: 4

Figure S44. Spatial Distribution Functions (SDFs): TEMPO plots.

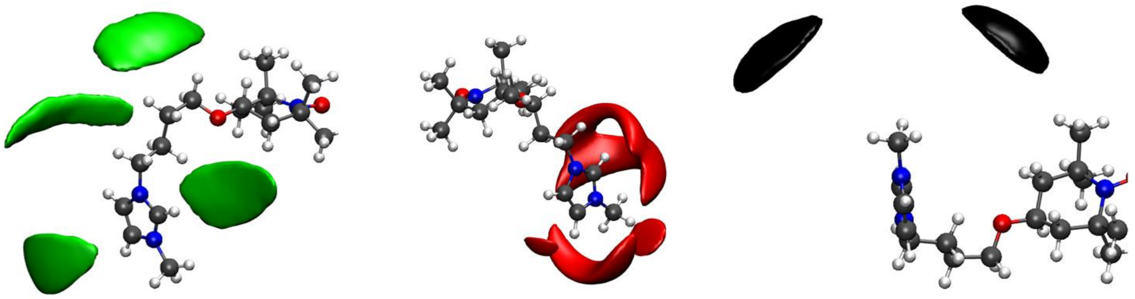

Imidazolium-TEMPO isovalue: 3 | **Imidazolium-[NTf$_2$]$^-$** isovalue: 8 | **Imidazolium- Imidazolium** isovalue: 3

Figure S45. Spatial Distribution Functions (SDFs): Imidazolium plots.

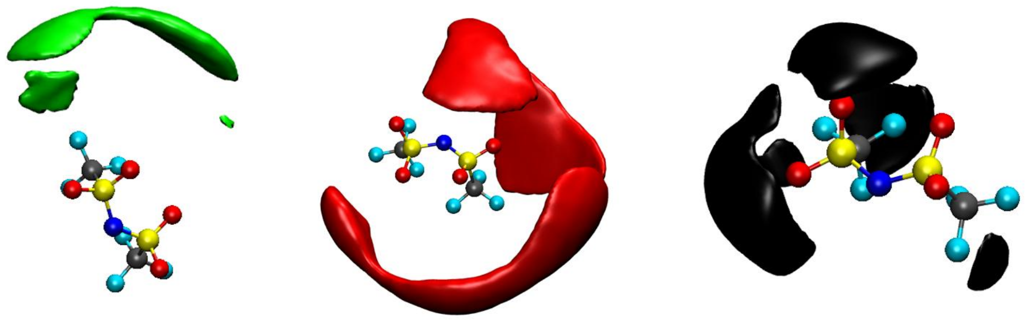

[NTf$_2$]$^-$-TEMPO isovalue: 1.7 | **[NTf$_2$]$^-$- [NTf$_2$]$^-$ (CoM)** isovalue: 3 | **[NTf$_2$]$^-$-imidazolium** isovalue: 7

Figure S46. Spatial Distribution Functions (SDFs): [NTf$_2$]$^-$ plots.

MATLAB simulations

All EPR spectral simulations were performed in MATLAB R2023b, using *EasySpin* 5.2.36[22] simulation software and/or home-written functions (the analysis and simulation programs are available from the Imperial Research Data repository: DOI: 10.14469/hpc/14504). Briefly, for the ^{19}F ENDOR simulations the simulation code used the EasySpin function “saffron.m”, using the spin system parameters determined from the simulation of the EDFS spectrum as shown in Figure S.3.3, with the number of orientations (knots) for which the spectra are explicitly calculated set to 91. A skewed normal distribution of hyperfine coupling tensors was then defined using the MATLAB function “normpdf” with a mean, μ, and standard deviation, σ, evaluated for a range of hyperfine couplings. The ENDOR spectrum was then calculated for each hyperfine coupling tensors (considering a purely dipolar interaction, [-*T* -*T* 2*T*]), and were weighted according to the defined probability density function. The final spectrum was then convoluted by a Lorentzian linewidth as defined in the text. The ^{2}H ENDOR simulations were also simulated using the EasySpin function “saffron.m” assuming a purely dipolar interaction as described in the main text and S.I.

S.5 Room temperature continuous wave EPR spectra

Spin-labelled ILs

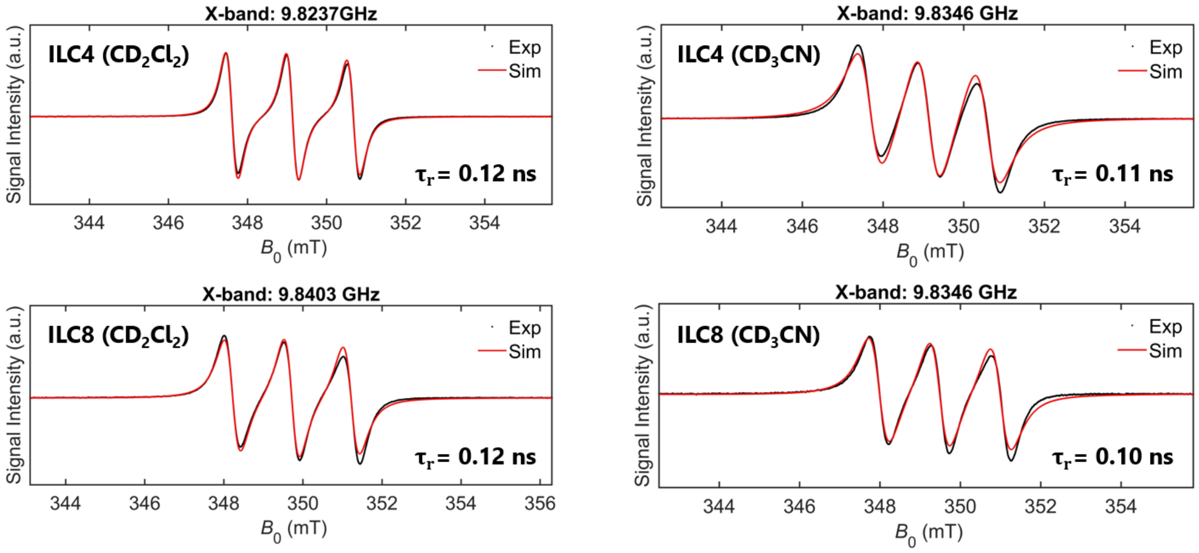

Figure S47. Experimental room temperature continuous wave EPR spectra at X-band frequencies (black) of the spin-labelled ILs, **[ILC4][NTf$_2$]** and **[ILC8][NTf$_2$]** in CD_2Cl_2 (left) and CD_3CN (right), and the corresponding best fitting spectral simulations using the *EasySpin* function 'garlic.m', with the calculated rotational correlation time, τ_r, shown. Representative simulation parameters: g = [1.9734, 2.0479, 2.0096]; A = [21.25, 17.84, 90.48] MHz.

The differing spectral lineshape observed in CD_3CN compared to CD_2Cl_2 is likely due to the fact that CD_3CN is a lossy solvent with a high dielectric constant, which can affect the phase of the microwave signal, with potentially some admixture from the dispersion component, which is broader than the absorption component. Further, small differences in the viscosity of these solvents will result in different tumbling rates of the spin-labelled ILs in solution, which then evolves as a broadening of the spectral linewidth.

S.6 ^{19}F paramagnetic relaxation enhancement (PRE) measurements

All ^{19}F NMR spectra were recorded at 298 K on a Bruker AVANCE 500 MHz spectrometer, equipped with a CPP1.1 BBO 500S2 BB-H&F-D-S Z XT probe operating at 470.554 MHz with a 20 Hz spin during acquisition. ^{19}F relaxation measurements were collected as 1D spectra with variable relaxation delays, using an 18 μs radiofrequency (RF) pulse at 11 W power, with a single scan per delay time, employing a Carr-Purcell-Meiboom-Gill (CPMG) sequence and an 8-step phase cycle. The FT NMR spectra were baseline corrected and phased using MestReNova software, from which the chemical shifts and spectral intensities were determined. The exponential fits to the relaxation data were processed using Bruker Dynamic Centre software. However, Γ_2 rates were determined using a two-time point measurement, allowing for the direct determination of Γ_2 without making use of any fitting procedures, nor requiring identical concentrations for the paramagnetic and diamagnetic samples. Here, the Γ_2 rates were determined from two-time points along the spin-spin relaxation curve (T_a = 0.008 and T_b = 0.073) according to:

$$\Gamma_2 = \frac{1}{T_b - T_a} \ln\left(\frac{I_{dia}(T_b) I_{para}(T_a)}{I_{para}(T_b) I_{dia}(T_a)}\right)$$

where I_{dia} and I_{para} are the peak intensities for the diamagnetic and paramagnetic states, respectively.

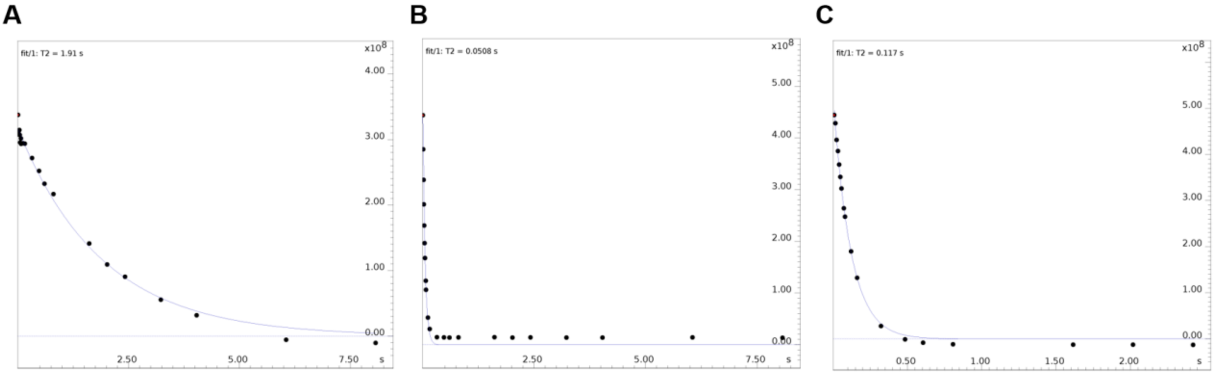

Figure S48. ^{19}F CPMG relaxation curves of **A)** [C_8C_1im][NTf_2] **B)** [ILC4][NTf_2] **C)** [ILC8][NTf_2] in CD_2Cl_2 fitted to a monoexponential decay of the form, $f(t) = a_0 . e^{\left(-\frac{t}{T}\right)}$.

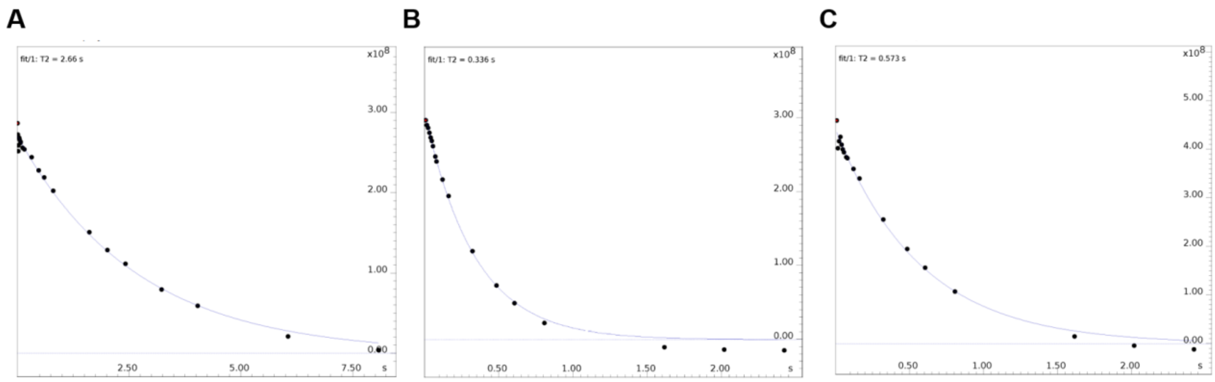

Figure S49. [19]F CPMG relaxation curves of **A)** [C_8C_1im][NTf_2] **B)** [ILC4][NTf_2] **C)** [ILC8][NTf_2] in CD_3CN fitted to a monoexponential decay of the form, $f(t) = a_0 . e^{\left(-\frac{t}{T}\right)}$.

S.7 Dynamic Light Scattering (DLS)

Size distributions measurements were attempted using a Zetasizer Ultra instrument (Malvern Instruments Ltd) at 25 °C and a small-volume quartz cuvette (Hellma) containing a 50 μL solution of **[ILC4][NTf$_2$]** dissolved in [d_3-C_8C_1im][NTf$_2$] (0.2 mM). All measurements were performed using Multi-Angle Dynamic Light Scattering (MADLS). The intensity-weighted distributions were calculated with the instrument-specific software ZS Xplorer (Version 1.3). The setting for the sample viscosity was set to η = 120 mPa s, and the refractive index set to n = 1.4.

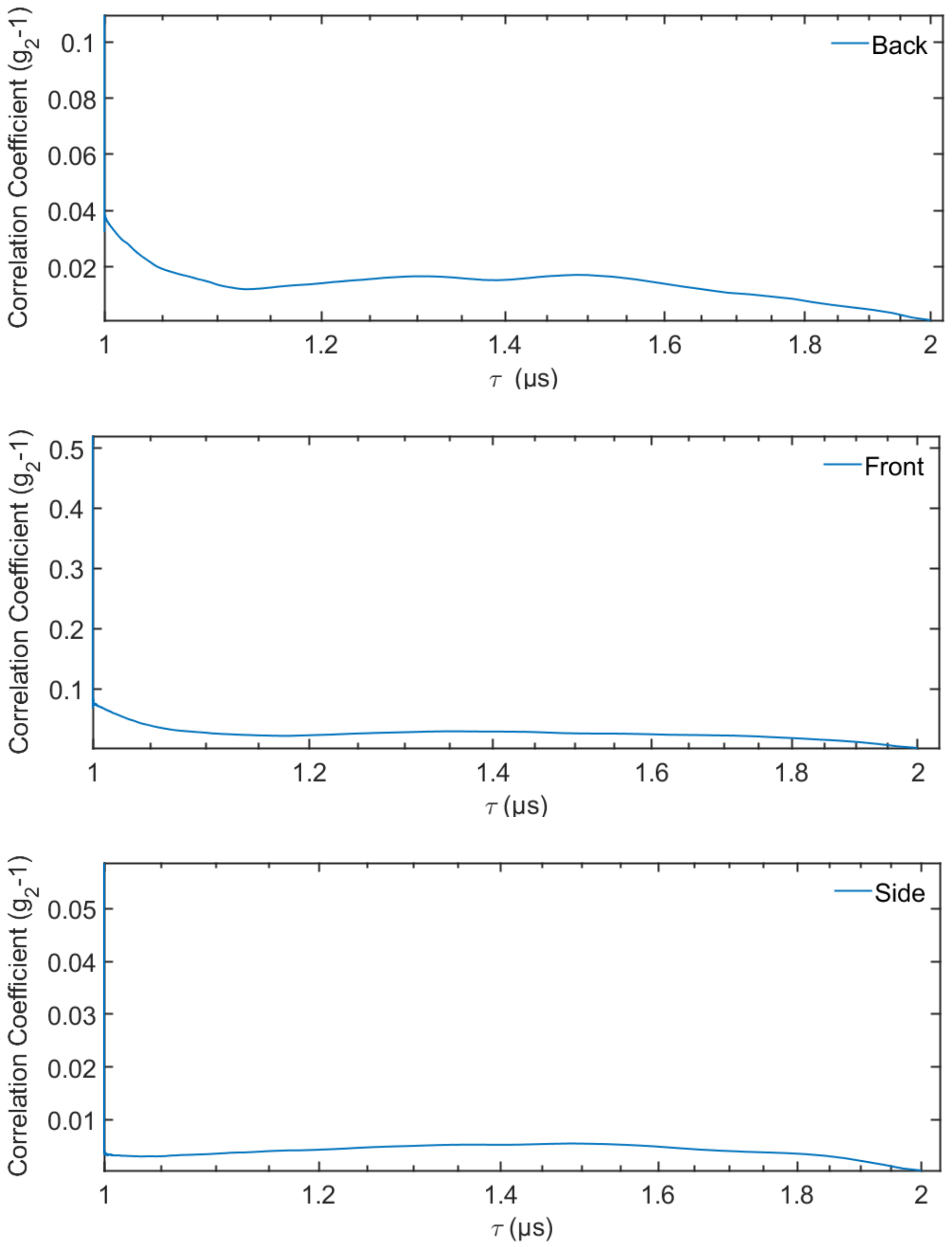

Figure S50. DLS MADLS results showing the intensity of the correlation coefficient *vs* τ in microseconds plotted on a logarithmic scale, for **[ILC4][NTf$_2$]** dissolved in [d_3-C_8C_1im][NTf$_2$] (0.2 mM).

S.8 Optimised DFT coordinates

Atomic coordinates of the DFT optimised structure of [ILC4][NTf2]

N	-3.26081141156567	0.42926029709427	-0.04205573550097
C	-2.27529999271615	-0.37970000016761	-0.44749999500082
N	-2.82530000305014	-1.45710000001186	-1.01979999956864
C	-4.20340000001600	-1.33410000276559	-0.97629999761715
C	-4.47569999430869	-0.15309999272821	-0.35680000642750
C	-3.06420014950872	1.68789992325406	0.70420006649091
C	-3.09869975536210	1.47300009258924	2.21739986265427
C	-2.07627159514504	-2.56604615839682	-1.62374688914580
H	-1.21189999921079	-0.18770000806994	-0.30079999812821
H	-4.85600000185774	-2.08370000319310	-1.38789999767593
H	-5.41219999839623	0.31999999993530	-0.11960000325355
H	-2.09939998898320	2.09430000341898	0.40080000135008
H	-3.84949997117025	2.37090001778307	0.37679998542378
H	-2.99660009356491	2.46419999247125	2.67170006454462
H	-4.07460086120975	1.07113073910441	2.50164402664224
H	-2.14827823285108	-2.50281358257535	-2.70969068938020
H	-2.49989999885906	-3.50739999907390	-1.27729999862664
H	-1.03800000145520	-2.49480000044270	-1.30200000129048
N	0.58700000199082	0.29450000552134	0.17799999644663
S	1.53630000456993	-1.01640000247601	0.21089999435155
S	1.07700000226756	1.81959999763242	-0.06009999845977
C	1.87749995699303	-1.21409998700442	2.05550002758951
O	0.65669999882677	-2.14939999781109	-0.10440000239505
O	2.83610000173654	-0.90910000045362	-0.42800000434816
F	2.65110000757894	-2.28339999990216	2.25440000044470
F	2.48940001136664	-0.13310000583679	2.53549999373580
F	0.72670002478020	-1.38810000860004	2.72329998117577
C	0.96589999857647	1.96909999992767	-1.93870000186691
O	-0.01719999784018	2.67319999689092	0.41059999421266
O	2.45049999886462	2.13080000198845	0.29010000024384
F	1.22700000216855	3.22149999980514	-2.31429999914606

F	1.81889999714578	1.14240000242196	-2.53830000209120
F	-0.28369999783718	1.65450000106246	-2.34259999929483
C	-1.98500006456930	0.55889997500205	2.73350003269138
H	-1.79886880429610	-0.25646131164914	2.03573683435345
H	-1.04608276923351	1.11537244916722	2.81105739825150
C	-2.31905557838745	-0.04193105742637	4.09761140172607
O	-3.52427405232531	-0.82257937439113	4.05220519804938
C	-3.40915456676367	-2.24934361636836	3.93390190588101
C	-2.90708289317288	-2.89215050830803	5.22085059077703
C	-2.98029116862228	-4.42761535557913	5.19119989919659
N	-2.40430443591344	-4.94036268230955	3.90227157339354
C	-2.52619102419474	-4.23446689112482	2.58122812960214
C	-2.52529934185547	-2.70899962040813	2.78104393874387
H	-4.44070534628239	-2.55675372350374	3.75090043783649
H	-3.49722353580638	-2.53960478724080	6.07044346870116
H	-1.86843214294418	-2.59310774108198	5.39119601521691
H	-2.85115374314171	-2.25574544511012	1.84264708813515
H	-1.49952445710210	-2.38485417236585	2.96615950192516
C	-2.13581159875035	-4.98968607865756	6.34100322468686
H	-2.21529643259289	-6.07507622832677	6.39541915913524
H	-1.08495689538335	-4.71832354475150	6.21486416177056
H	-2.49443181588686	-4.56441922112716	7.28142002337481
C	-4.43114257267429	-4.92664401605867	5.32909248795217
H	-5.09426300298939	-4.48819348312106	4.58268959611115
H	-4.46145545091221	-6.01296193392722	5.22629364184500
H	-4.80942479278930	-4.65898969651211	6.31872981418618
C	-1.30283761766466	-4.61982102909655	1.73998877639052
H	-1.32391532608182	-5.67672905829062	1.47482071494201
H	-1.29542688129721	-4.02532153549510	0.82563177782659
H	-0.38195423951714	-4.41085896370955	2.28905019272327
C	-3.81086533735536	-4.70028800604243	1.87114951277867
H	-3.81820642210659	-4.30158306013146	0.85348055545297
H	-3.83465421202760	-5.79019814745769	1.81963381150308
H	-4.71293358750536	-4.35444541554977	2.37737421077195

O	-2.15866557157159	-6.19803831210643	3.84910998088286
H	-1.48024055364714	-0.63410928474825	4.47147605228605
H	-2.50603094967121	0.75516441821702	4.82350732112729

Atomic coordinates of the DFT optimised structure of [ILC8][NTf_2]

N	-3.26094612613289	0.42921928033216	-0.04123079789075
C	-2.27529999415068	-0.37970000101565	-0.44749998980571
N	-2.82529999988202	-1.45710000016595	-1.01979999653720
C	-4.20340000198431	-1.33410000531299	-0.97629999400893
C	-4.47569999226651	-0.15309997667298	-0.35680003961921
C	-3.06420024513512	1.68789987600963	0.70420008093554
C	-3.09869949546476	1.47300033418305	2.21739986651111
C	-2.07627159302392	-2.56604616182157	-1.62374688755154
H	-1.21189999703767	-0.18770001535995	-0.30079999554095
H	-4.85599999931054	-2.08370000278263	-1.38789999435330
H	-5.41219999860419	0.31999999357702	-0.11959999143900
H	-2.09939997242929	2.09429994429804	0.40079997913197
H	-3.84949996543675	2.37090002765204	0.37679998896867
H	-2.99660016831617	2.46419995050059	2.67170006987232
H	-4.07460089770930	1.07113068946952	2.50164403725071
H	-2.14827823349650	-2.50281358569353	-2.70969068960177
H	-2.49989999865893	-3.50739999874025	-1.27729999945798
H	-1.03800000125462	-2.49479999689869	-1.30200000432553
N	0.58699999923579	0.29450001046394	0.17799998937238
S	1.53630000708151	-1.01640000515925	0.21089999289943
S	1.07700000158437	1.81960000124190	-0.06010000219364
C	1.87749995628260	-1.21409998331809	2.05550002879004
O	0.65669999804309	-2.14939999949429	-0.10439999955244
O	2.83610000052762	-0.90910000066800	-0.42800000221615
F	2.65110000933711	-2.28339999802912	2.25440000198179
F	2.48940001246479	-0.13310000638785	2.53549999317064
F	0.72670003122592	-1.38810000957296	2.72329997408697
C	0.96589999856690	1.96909999956445	-1.93870000266386
O	-0.01720000133092	2.67320001956014	0.41060000908930

O	2.45049999901904	2.13080000124411	0.29009999916445
F	1.22700000179396	3.22149999967552	-2.31429999888165
F	1.81889999748323	1.14240000255469	-2.53830000226680
F	-0.28369999773632	1.65450000061308	-2.34259999931661
C	-1.98500019948404	0.55889987110925	2.73350000777261
H	-1.79886875119173	-0.25646128478867	2.03573684329546
H	-1.05190853749252	1.12248963608466	2.80411328799269
C	-2.33684651795026	-0.10769523194258	4.07589904958234
H	-1.41896967098730	-0.42096842576599	4.58519852720404
H	-2.83017496647765	0.61395056556490	4.73691355282686
C	-3.23750806046658	-1.33748755678673	3.86141004985149
H	-3.67322704875973	-1.65892790042131	4.81272162675225
H	-4.08265733040179	-1.04830373217984	3.22452219541247
C	-2.48706401802593	-2.51372458166536	3.20589405153852
H	-1.64360500212809	-2.13328245712853	2.62373162765228
H	-2.04919982503109	-3.14911569675092	3.98466239087887
C	-3.35561299756128	-3.35582995684424	2.26810665055222
H	-4.19957361516008	-3.77959280354661	2.82118334129861
H	-3.77493799000029	-2.70849100306262	1.49075316475064
C	-2.53259495262433	-4.45701206053604	1.59921657515962
H	-1.59965325281271	-4.02505022477329	1.22799381917598
H	-2.26397140634973	-5.22934559791767	2.32826080000135
C	-4.26322872875932	-5.90956004775859	0.56068774955266
C	-5.57523908748188	-5.13264260162918	0.52417845721318
C	-6.80718128557691	-6.04716869819655	0.42451646722292
N	-6.69235730970720	-7.16277079460251	1.42621100950114
C	-5.39631779797000	-7.82511341223050	1.80283470616221
C	-4.25187905555438	-6.79520793203473	1.79814050397301
H	-4.19471815952036	-6.53516769318469	-0.33199651020110
H	-5.58363391612739	-4.45230387642118	-0.33126248050788
H	-5.66310407142262	-4.52607050676598	1.42759926579183
H	-3.30967047511270	-7.34419956737313	1.87412428814119
H	-4.34439178402912	-6.17178461148146	2.69208841538290
C	-8.05901313449335	-5.23298854081687	0.77380295844511

H	-8.96331816500323	-5.82888256008132	0.65338073185915
H	-8.00801770597314	-4.87289073487664	1.80410700725804
H	-8.11602742965002	-4.37031507086513	0.10556072622429
C	-6.96413463897655	-6.64581005185157	-0.98621518061995
H	-6.06149811378266	-7.15245113960207	-1.32828178366085
H	-7.78811513443436	-7.36178210261637	-0.99455160565780
H	-7.19201050786471	-5.84409678737184	-1.69311961188811
C	-5.55272996022568	-8.37975109546975	3.22499898066159
H	-6.30641936902253	-9.16590728751205	3.25952095079754
H	-4.59576683672661	-8.79651026068655	3.54811111148698
H	-5.83882684104425	-7.58532121887828	3.91834890798677
C	-5.11850047800232	-8.98756236652935	0.83067249029081
H	-4.25500703440312	-9.55430053923656	1.18747052731611
H	-5.98208555878859	-9.65420237855948	0.79544583474285
H	-4.90312316185806	-8.64417594456640	-0.18149434034957
O	-7.75861340569326	-7.83924633929052	1.64984703937993
O	-3.11625703882596	-5.05187060551100	0.43330629706438

References

[1] S. Koutsoukos, J. Becker, A. Dobre, Z. Fan, F. Othman, F. Philippi, G. J. Smith, T. Welton, *Nat. Rev. Methods Primers* **2022**, *2*, 1–18.

[2] S. Koutsoukos, J. Avila, N. J. Brooks, M. C. Gomes, T. Welton, *Phys. Chem. Chem. Phys.* **2023**, *25*, 6316–6325.

[3] F. Philippi, D. Rauber, O. Palumbo, K. Goloviznina, J. McDaniel, D. Pugh, S. Suarez, C. C. Fraenza, A. Padua, C. W. M. Kay, T. Welton, *Chem. Sci.* **2022**, *13*, 9176–9190.

[4] R. Giernoth, D. Bankmann, *Tetrahedron Lett.* **2006**, *47*, 4293–4296.

[5] S. H. Bossmann, N. D. Ghatlia, M. F. Ottaviani, C. Turro, H. Dürr, N. J. Turro, *Synthesis* **2000**, *1996*, 1313–1319.

[6] M. Yu. Ivanov, O. D. Bakulina, Y. F. Polienko, I. A. Kirilyuk, S. A. Prikhod'ko, N. Yu. Adonin, M. V. Fedin, *J. Mol. Liq.* **2023**, *381*, 121830.

[7] L. Fábregas Ibáñez, J. Soetbeer, D. Klose, M. Tinzl, D. Hilvert, G. Jeschke, *J. Magn. Reson.* **2019**, *307*, 106576.

[8] F. Neese, *Wiley Interdiscip. Rev.: Comput. Mol. Sci.* **2022**, *12*, e1606.

[9] M. J. Frisch *et al.*, Gaussian 16, Revision C.01 , Gaussian, Inc., Wallingford CT, **2016**.

[10] S. Grimme, S. Ehrlich, L. Goerigk, *J. Comp. Chem.* **2011**, *32*, 1456–1465.

[11] G. A. Zhurko, D. A. Zhurko, Chemcraft - graphical software for visualization of quantum chemistry computations, Version 1.8, build 682. https://www.chemcraftprog.com

[12] E. F. Pettersen, T. D. Goddard, C. C. Huang, G. S. Couch, D. M. Greenblatt, E. C. Meng, T. E. Ferrin, *J. Comput. Chem.* **2004**, *25*, 1605–1612.

[13] K. Goloviznina, J. N. Canongia Lopes, M. Costa Gomes, A. A. H. Pádua, *J. Chem. Theory Comput.* **2019**, *15*, 5858–5871.

[14] R. Berthin, A. Serva, K. G. Reeves, E. Heid, C. Schröder, M. Salanne, *J. Chem. Phys.* **2021**, *155*, 074504.

[15] A. P. Thompson, H. M. Aktulga, R. Berger, D. S. Bolintineanu, W. M. Brown, P. S. Crozier, P. J. in 't Veld, A. Kohlmeyer, S. G. Moore, T. D. Nguyen, R. Shan, M. J. Stevens, J. Tranchida, C. Trott, S. J. Plimpton, *Comput. Phys. Commun.* **2022**, *271*, 108171.

[16] L. Martínez, R. Andrade, E. G. Birgin, J. M. Martínez, *J. Comp. Chem.* **2009**, *30*, 2157–2164.

[17] L. Wylie, G. Perli, J. Avila, S. Livi, J. Duchet-Rumeau, M. Costa Gomes, A. Padua, *J. Phys. Chem. B* **2022**, *126*, 9901–9910.

[18] K. Goloviznina, M. Salanne, *J. Phys. Chem. B* **2023**, *127*, 742–756.

[19] M. Brehm, M. Thomas, S. Gehrke, B. Kirchner, *J. Chem. Phys.* **2020**, *152*, 164105.

[20] W. Humphrey, A. Dalke, K. Schulten, *J. Mol. Graphics* **1996**, *14*, 33–38.

[21] L. Wylie, J. P. Barham, B. Kirchner, *ChemPhysChem* **2023**, *24*, e202300470.

[22] S. Stoll, A. Schweiger, *J. Magn. Reson.* **2006**, *178*, 42–55.

[23] Chen, C.H. Deuterium Bonding Versus Hydrogen Bonding. In *Deuterium Oxide and Deuteration in Biosciences*; Chen, C.-H., Ed.; Springer International Publishing, 2022; pp 29-42.

[24] A. Zoleo, R. G. Lawler, X. Lei, Y. Li, Y. Murata, K. Komatsu, M. Di Valentin, M. Ruzzi, N. J. Turro, *J. Am. Chem. Soc.* **2012**, *134*, 31, 12881–12884.
